# A fluctuation test for gastruloid heterogeneity

**DOI:** 10.1016/j.cell.2026.09.057

**Published:** 2026-10-08

**Authors:** Samuel G. Regalado, Chengxiang Qiu, Sanjay Kottapalli, Chau Huynh, Riza M. Daza, Aidan Keith, Jihye Park, Beth K. Martin, Wei Chen, Hanna Liao, Haedong Kim, Xiaoyi Li, Jean-Benoît Lalanne, Nobuhiko Hamazaki, Silvia Domcke, Junhong Choi, Jay Shendure

**Affiliations:** 1. Department of Genome Sciences, University of Washington, Seattle, WA 98195, United States; 2. Medical Scientist Training Program, University of Washington, Seattle, WA 98195, United States; 3. Current address: Department of Medicine, University of California, San Francisco, San Francisco, CA 94143, United States; 4. Seattle Hub for Synthetic Biology, Seattle, WA 98109, United States; 5. Department of Molecular and Systems Biology, Dartmouth College, Hanover, NH 03755, United States; 6. Developmental Biology Program, Memorial Sloan Kettering Cancer Center, New York, NY 10065, United States; 7. Institute for Protein Design, University of Washington, Seattle, WA 98195, United States; 8. Molecular and Cellular Biology Program, University of Washington, Seattle, WA 98195, United States; 9. Howard Hughes Medical Institute, Seattle, WA 98195, United States; 10. Current address: Department of Biochemistry and Molecular Medicine & Courtois Institute for Biomedical Innovation, Université de Montréal, Montréal, QC H3T 1J4, Canada; 11. Institute of Stem Cell and Regenerative Medicine, University of Washington, Seattle, WA 98109, United States; 12. Brotman Baty Institute for Precision Medicine, Seattle, WA 98195, United States; 13. Department of Obstetrics & Gynecology, University of Washington, Seattle, WA 98195, United States; 14. Current address: Department of Molecular Life Sciences, University of Zurich, CH-8057 Zurich, Switzerland; 15. Allen Discovery Center for Cell Lineage Tracing, Seattle, WA 98109, United States; 16. AI BioDesign, Seattle, WA 98109, United States; 17. Allen Institute, Seattle, WA 98109, United States; 18. These authors contributed equally; 19. Lead contact

## Abstract

Stem cells self-organize into models of early development, but even under carefully controlled conditions, the resulting organoids exhibit substantial “inter-individual” heterogeneity. To explore its origins, we generated monoclonal gastruloids, each derived from a single mouse embryonic stem cell (mESC). Molecular profiling revealed extensive heterogeneity: some failed to develop, others were biased toward mesodermal or neural fates, and yet others contained cell types atypical of conventional polyclonal gastruloids. Applying DNA Typewriter, a cell lineage recorder, we identified heritable fate biases arising prior to gastruloid induction, and found that more closely related founder mESCs give rise to more phenotypically similar gastruloids. Linking mESC subclones to their descendants’ fates revealed cryptic, fate-predictive differences in gene expression and chromatin accessibility, including at imprinted, methylation-sensitive loci linked to developmental potency. Intrinsic, heritable fluctuations in mESC state are therefore an important source of organoid heterogeneity, and DNA Typewriter enables high-resolution lineage trees in stem cell models.

## IN BRIEF

Organoids grown side by side under identical conditions vary unpredictably, a longstanding obstacle to their use. Adapting the logic of a classic fluctuation test to gastruloids grown from single stem cells shows that this variability is largely inherited rather than induced, traceable to fluctuations in the state of the founding cell.

## Graphical Abstract

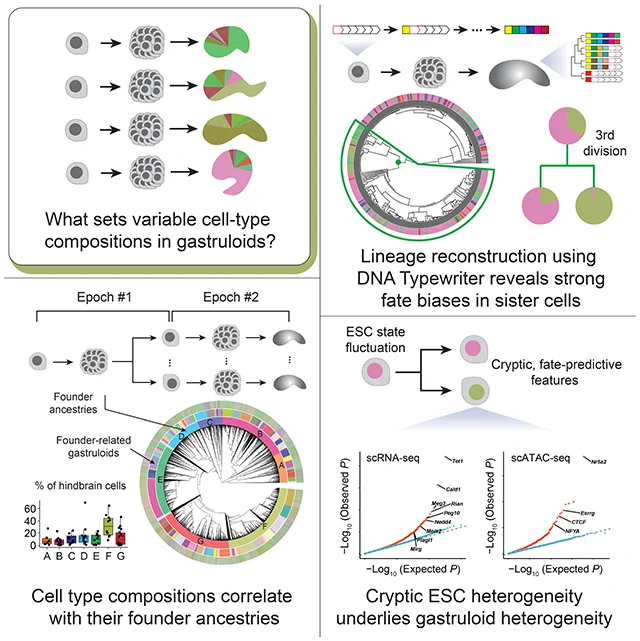

## INTRODUCTION

Stem cell-derived models are powerful tools for studying cell fate decisions in early mammalian development^1,2^. Conventionally, a polyclonal aggregate of ESCs or induced pluripotent stem cells^3,4^ is subjected to a specific regimen of external stimuli that mimics *in vivo* lineage specification, resulting in models of the developing brain^5,6^, gut^7^, retina^8^, kidney^9^, heart^10^ and other organs, as well as early structures such as the blastula^11^, gastrula^12–15^ and post-gastrulation embryo^16,17^ (note: here, “organoid”: all stem cell-derived *in vitro* models; “gastruloid”: the specific mouse gastruloid model used here^12^).

Inter-organoid heterogeneity is a longstanding challenge in this field^18–20^, pervasive even in batches of organoids derived under carefully controlled conditions within a single experiment. Although generally treated as a nuisance, what underlies this heterogeneity is itself of considerable interest. One possibility is that fluctuating internal states within seemingly uniform stem cell populations produce different responses to identical inductive cues. This is consistent with transcriptional heterogeneity observed in stem cell lines^21,22^, and with evidence that state fluctuations contribute to core-periphery differences in stem cell spheroids prior to induction^23^. Recent reports also show mESC culture conditions prior to aggregation can bias lineage composition and developmental trajectories in gastruloids^24,25^.

However, because conventional protocols aggregate hundreds to thousands of stem cells, each aggregate would be expected to sample any such heterogeneity similarly. Intra-batch heterogeneity might therefore arise peri- or post-induction instead, from cell-extrinsic factors such as spheroid size or differential morphogen exposure^26^, or from stochastic events within or among the aggregated cells. Distinguishing these possibilities is difficult because conventional aggregates comprise stem cells with potentially diverse internal states and unknown lineage relationships, making it hard to ask whether heterogeneity is determined before, during or after induction.

To simplify the question, we developed a protocol for producing monoclonal stem cell aggregates^27^, wherein cells giving rise to each aggregate compose a clone whose members are as closely related as possible, and then induced these to form mouse gastruloids, which model symmetry breaking, axial elongation and germ layer specification^12,13^. Leveraging scRNA-seq, we find that monoclonal gastruloids exhibit inter-individual heterogeneity exceeding that of conventional polyclonal gastruloids. To probe the heritability of internal state fluctuations, we engineered DNA Typewriter^28^ into mESCs and reconstructed lineage relationships within a series of monoclonal gastruloids. We observe fluctuations in mESC state that shape descendants’ post-induction fates, arising early in the expansion of founder cells but well before induction. Within a family of mESCs founding ~100 gastruloids, more closely related founders gave rise to more phenotypically similar gastruloids. Finally, profiling nominally homogeneous mESC subclones uncovered cryptic, fate-predictive differences in gene expression and chromatin accessibility, connecting these heritable biases to specific stem cell regulators and regulatory states.

## RESULTS

### A protocol for generating monoclonal gastruloids

To generate monoclonal mouse gastruloids, we plate dissociated mESCs on mouse embryonic fibroblast (MEF) feeders at low density. After five days, mESC colonies are released from the feeder layer using collagenase IV treatment and cultured for a further day on a non-adherent plate to form a spherical aggregate. Each monoclonal aggregate is then induced to form a mouse gastruloid with a 24-hour CHIR99021 (CHIR) pulse^12^, generally yielding an elongated structure resembling a conventional mouse gastruloid (Figure S1A–B; STAR Methods).

To test monoclonality, we constructed a polyclonal mESC line bearing randomly integrated static DNA barcodes. Of 234 aggregates derived from this line by the monoclonal gastruloid protocol, 201 yielded sufficient barcode sequencing coverage, and 187 of these (93%) were dominated by a single barcode (≥90% of reads; Figure S1C,D). Of the remaining 14, 13 had two and 1 had three dominant barcodes, possibly di- or tri-clonal aggregates, or alternatively, monoclonal founders harboring multiple lentiviral integrants. The protocol therefore yields gastruloids that overwhelmingly derive from a single founder mESC.

### Inter-individual heterogeneity among monoclonal gastruloids

To characterize heterogeneity among monoclonal gastruloids, we engineered mESCs by integrating two cassettes: one bearing both DNA Typewriter Tape (DTT) and DTT-targeting enhanced prime editing guide RNAs (epegRNAs), and the other encoding a doxycycline-inducible prime editor (inducible PEmax or iPEmax) (Figure S1E–G). Each DTT included a static DNA barcode (12N; “TapeBC”) upstream of a six-unit monomeric array that serves as substrate for sequential editing by DNA Typewriter. Cells in the resulting polyclonal line were expected to harbor a unique set of TapeBC-tagged DTTs and at least 1 copy of iPEmax.

In this experiment, we used the DTTs for tracking clonality, rather than to reconstruct cell lineage trees. Prior to FACS and single-cell seeding, a 24-hr doxycycline pulse induced PEmax to edit DTT and thereby boost barcode diversity. We collected 144 putatively monoclonal gastruloids on day 12 (equivalent to 144 hours in the conventional protocol), and pooled their nuclei for sci-RNA-seq3^29^, modifying the protocol to boost DTT recovery^30^ (Figure S1H). In parallel, we isolated genomic DNA from cellular debris in residual media from each well, and performed amplicon sequencing of DTT to facilitate “gastruloid-to-well” assignments (“Debris-seq”) (Figure 1A).

After filtering low-quality profiles and potential doublets, we retained 247,064 cells (median 609 UMIs) for cell-type annotation based on gene markers and comparison with other datasets^23,31–35^ (Figure 1B; Figures S2–S5; Tables S1–S4). While many of the cell types observed in 144-hr monoclonal mouse gastruloids aligned with expectation as defined by four *in vitro* studies^23,31,34,35^ (*e.g.* cell types resembling neuromesodermal progenitors (NMPs; *T+*, *Cdx2*+), spinal cord/r7/r8 (*Hoxb4*+; *Hoxc6*+), mesodermal progenitors (*Tbx6*+, *Hes7*+), somites (*Tcf15*+, *Meox1*+), cardiac mesoderm (*Hand1*+; *Tbx20*+), definitive endoderm (*Sox17*+, *Trh*+), and endothelial cells (*Kdr*+, *Cdh5+*)), they also contained hindbrain-like cells (*Egr2*+), a subset of which expressed midbrainhindbrain boundary markers (*En1+*, *En2+*, *Pax5*+), and cells resembling early differentiating neurons (*Neurod1+*, *Ebf2+*, *Ebf3*+) that were transcriptionally contiguous with both spinal cord and hindbrain-like cells (Figure 1B; Figures S2–S5; Tables S1–S2). Additional abundant cell types included undifferentiated PSC-like cells, expressing both epiblast (*Nanog+*, *Utf1+*) and primordial germ cell (*Dppa3*+; *Dppa5a*+) markers, and “transitional” cells (*Bhlhe41*+; *Atp6v0b*+), seemingly intermediate between PSCs and germ layers but failing to express primitive streak markers. Rarer, unexpected cell types resembled the node (*Foxa2*+; *Kcnip4*+), notochord (*T+, Noto+*), floor plate (*Shh+*; *Foxa2*+), anterior mesendoderm (*Eomes*+; *Lhx1*+), and PSC-derived extraembryonic (ExE) endoderm-like cells (*Gata4*+, *Gata6*+). Of note, some of these have correlates in a subset of the conventional gastruloid studies^23,31,34,35^ but at lower proportions (*e.g.* neurons in Rosen *et al.* (2022)^34^), while others are entirely absent (*e.g.* PSC-derived ExE endoderm-like cells).

While collection time and subtle protocol differences could explain the observed differences (especially given clear differences between the conventional gastruloid studies in Figure S3; *e.g.* the paucity of neural lineage cells in Suppinger *et al.* (2023)^23^), another possibility is that outlier states are buffered by other clones in polyclonal gastruloids, thus unlikely to dominate the fate of a given gastruloid. In monoclonal gastruloids, the shared clonal origin of cells comprising the aggregate may eliminate such buffering, leading to unexpected cell types and compositions.

Although scRNA-seq was performed without sample indexing, we leveraged the coincidence of edited DTTs observed in cells (scRNA-seq) and residual media (Debris-seq) to assign each single-cell profile to its well-of-origin (Figure 1A; Figure S6A–B). After excluding 23 wells with no uniquely assigned TapeBCs or too few assigned cells, 148,724 cells were confidently assigned to 121 gastruloids/wells (1,229 ± 953 cells each; 25 ± 16 DTT/epegRNA integrants per founder, range 5-93). Reassuringly, there was strong one-to-one correspondence between the edited DTTs recovered by each method (Figure 1C).

To assess inter-gastruloid heterogeneity, we performed PCA on “pseudo-bulked” transcriptional profiles of monoclonal gastruloids. PC1 (35.7% of variance) separated gastruloids dominated by somite-like, spinal cord-like or hindbrain-like cells, while PC2 (16.5% of variance) correlated with the proportion of cells remaining in an undifferentiated PSC-like state (Figure 1D). Based on their cell type compositions, we clustered the 121 gastruloids into four groups, which were dominated by cells annotated as PSC-like (non-differentiating or ND), somites (MESO), spinal cord/r7/r8 (SC-NEURO), or hindbrain r1-r6 (HB-NEURO) (Figure 1E). Rarer cell types exhibited type-specific enrichments as well, *e.g.* early neurons and floor plate in HB-NEURO (Figure S6C). Furthermore, some cell types were overwhelmingly accounted for by a few gastruloids (Figure S6D). For example, just 3 gastruloids accounted for 59% of anterior mesoderm cells, 45% of mesodermal progenitor cells, and 46% of cardiac mesoderm cells. Recovery, which presumably correlates with the total number of cells in a gastruloid, did not significantly differ across the four groups (Figure S6E). However, from images taken prior to harvesting, composition-based group identity did correlate with gastruloids’ morphological appearances, with MESO and SC-NEURO gastruloids more likely to exhibit elongation (Figure 1F).

### Phylogenetic reconstruction of the cell lineage histories of monoclonal gastruloids

Given that the heterogeneity of monoclonal gastruloids was at least on par with, and possibly exceeded that of conventional polyclonal gastruloids, we sought to establish a framework for reconstructing their cell lineage histories. To this end, we established three monoclonal cell lines (Clone-05, Clone-25, Clone-32) equipped with DNA Typewriter^28^ (Figure S1E–F), and harboring at least 76, 116 and 84 DTT/epegRNA integrants (lower bounds, as some integrants are duplicated by piggyBac excision and re-integration events^30,36^). With six ordered units per DTT, each line harbors at least 400 writable sites (Figure S7).

We used a pool of three lines to generate monoclonal gastruloids with continuous doxycycline induction starting 24 hours before single-cell seeding (Figure 2A). On Day 10, we performed Debris-seq and rapidly analyzed the data in order to select eight gastruloids with high recording rates and elongated morphology to collect on Day 11, which corresponds to the 120-hr time point of the conventional protocol. We then performed scRNA-seq (10x Genomics) with feature enrichment to enhance DTT recovery.

Following conventional filtering, we obtained scRNA-seq profiles for 9,929 cells (median 10,881 UMIs, 3,611 genes), of which 86% were confidently assigned to a gastruloid by Debris-seq (mean 1,068 cells, range 312-2,696; Figure 2B–C; Figure S8A–C). We again observed a continuum of cell states that includes PSC-like cells differentiating and then bifurcating to both mesodermal and neural states of varying maturity. Among eleven annotated cell types, six accounted for >98% of cells (PSC-like, transitional, NMPs, mesodermal progenitors, somites, spinal cord) (Figure 2C; Figure S8D; Table S1). Several cell types observed in the 144-hour experiment were absent here, presumably due to time point, technical and/or sampling differences, while one was only detected here (fibroblasts).

For lineage reconstruction, we focused on 7,816 higher-quality cells, for which we recovered 45 ± 13 edited DTTs/cell (3.0 ± 1.5 edits/edited DTT; mean 138 ± 47 edits/cell; Figure S8E). Interestingly, the cumulative activity of DNA Typewriter varied modestly between cells from different gastruloids, even those derived from the same clone, but was relatively consistent across cell types within a gastruloid (Figure S9). To account for piggyback excision and re-integration events, we split patterns observed at some TapeBCs prior to tree building (Figure S10; STAR Methods). Phylogenetic reconstruction was performed by UPGMA with a custom DTT-based distance metric (Figure 2D; Figures S11–S13; STAR Methods).

Across 100 bootstrap resamples, 48% (Clone-05), 77% (Clone-25) and 60% (Clone-32) of nodes had moderate to strong support (>70%) of transfer bootstrap expectation (TBE)^37^. Poorly supported nodes were overwhelmingly shallow, while deep nodes were strongly supported (Figure S8F). The deepest nodes corresponded to 10 major clades, each of whose membership overwhelmingly (~99%) derived from just one of the eight monoclonal gastruloids (Figure 2D; Figure S8G). These clades bore numerous “gastruloid-defining edits” (Figure S14), which we infer to have occurred in the 24-hr interval between PEmax induction and seeding of single founder cells.

What is the relationship between cell lineage and cell state? In each monoclonal gastruloid, phylogenetic distance correlated positively with transcriptional state distance^38^ (Figure 2E). We assessed this further through terminal-neighbor frequencies (Figure S15A–B) and PATH^39^-based phylogenetic cross-correlation (Figure S15C–D). For six abundant cell types, a PATH-derived state-transition graph was broadly consistent with expectation, except for the directionality of the transition between PSC-like cells and transitional cells (Figure 2F), possibly due to the persistent self-renewal of PSC-like cells refractory to differentiation signals.

### Strong fate biases arise within a few cell divisions of monoclonal stem cell seeding

As with the 121 x 144-hr gastruloids, these 8 x 120-hr gastruloids exhibited substantial heterogeneity. Most strikingly, Clone-05 produced well-balanced (Well14), neurally-biased (Well17) and mesodermally-biased (Well25) gastruloids (Figure S16). However, there was also clear structure within the phylogenetic trees of individual gastruloids (Figure 3A; Figure S15C).

One clear example involves PSC-derived, ExE endoderm-like cells, a cell type atypical for conventional polyclonal gastruloids (Figure S2D; Figure S5). Remarkably, 94% (17/18) of these cells appear in just two homogeneous clades, both from Clone-32 gastruloids, inferred to have derived from the second (Well16 gastruloid) or fourth (Well28 gastruloid) cell division (Figure 3B; Figure 2C). A reasonable inference is that the ancestral mESC spontaneously differentiated into an ExE endoderm-like state^40^ during clonal expansion, before lifting from the feeder layer, and persisted through aggregation and CHIR treatment. Strong phylogenetic structure was also observed for other annotations, *e.g.* cell divisions for which one daughter primarily gave rise to PSC-like cells vs. other cell types (Figure 3C), or mesodermal vs. neural cell types (Figure 3D).

Are these fate biases arising before or after induction? For each class of bias above, at least one example occurs within a few cell divisions of the gastruloid’s founding, at nodes with strong bootstrap support. For example, the fourth division of the Well28 gastruloid separates ExE endoderm-like cells (14/14) from all others (76/76) (q = 1e-15; Figure 3B); the first division of Well16 separates undifferentiated (271/331) from differentiated cell types (80/83) (q = 6e-41; Figure 3C); and the third division of Well25 separates mesodermal (102/127) from neural cells (30/31) (q = 2e-14; Figure 3D). Because phylogenetic reconstruction was blind to annotation, these distributions are highly unlikely to have occurred by chance. And because each founder cell was initially expanded to 400-700 mESCs over seven days, they likely occurred well before CHIR-mediated induction.

### Cell type compositions of monoclonal gastruloids correlate with their founder ancestries

While the divergent fates observed among sister clades might reflect heritable fluctuations in intrinsic stem cell states, spatial segregation of daughter cells could also underlie the observed fate biases^23,26^. As a more rigorous test, we designed a two-epoch “tree-of-trees” experiment (Figure 4A). In the first epoch, a single mESC expands to a colony of 500-1000 cells. In the second epoch, this monoclonal colony is dissociated and replated to generate monoclonal gastruloids. Lineage recording from the first epoch reports the phylogenetic relationships among founders, while the dissociation disrupts their spatial organization; the design also constrains how distantly related any two founders can be.

Implementing this design with Clone-05, we generated 108 monoclonal gastruloids (Figure 4A). We harvested the gastruloids at Day 18 (equivalent to 144 hrs for conventional gastruloids). We then performed subsequent steps as in the prior experiment (*i.e.* scRNA-seq of transcriptomes and DTT, cell-to-well assignment via Debris-seq, phylogenetic reconstruction; STAR Methods).

A phylogenetic tree based on Jaccard similarity between gastruloid-defining edits (Figure 4B, top) was robust to bootstrapping analysis, with 99% (106/107) ancestral nodes showing moderate-to-strong support (TBE>70%) (Figure S17A). Each gastruloid was assigned to one of seven major clades, some of which subjectively exhibited different morphological characteristics. We recovered 66,978 transcriptomes spanning 18 annotated types, resembling earlier experiments but with more PSC-like cells and a few rare types not previously seen (2-cell-state-like ESCs, motor neurons, MHB-like cells) (Figure 4C; Figure S17B).

We then constructed a monophyletic cell lineage tree for 58,283 cells with sufficient DTT recovery, leveraging the same DTT-based metric (Figure 4D; Figure S18A; STAR Methods). Based on coincidence with edited DTTs observed in Debris-seq, seven major clades were labeled as pseudo-ancestors near the root (Figure 4B; Figure S18B–C). This procedure was iterated to label 96 ancestral nodes as pseudo-ancestors of monoclonal gastruloids; collectively, these contained 51,963 (89%) of cells in the tree (541 +/− 704 cells per gastruloid/pseudo-ancestor; range: 2-5,319; Figure S18D–E; STAR Methods). Assigned cell numbers strongly correlated with gastruloid size (Figure 4B, bottom; Figure S18F).

To assess whether the phenotypic variation among gastruloids (second epoch) is informed by the lineage relationships of their founder mESCs (first epoch) (Figure 4A), we used two approaches: First, we simply asked whether the gastruloids of major clades A-G are enriched for specific cell types. Indeed, Clade A is enriched for gastruloids containing a high proportion of PSC-like cells (*p =* 8 x 10^−3^, Wilcoxon test compared to 5,000 permutations), and Clade F for gastruloids containing a high proportion of hindbrain cells (*p* = 2 x 10^−4^) (Figure 4E; Figure S19A). Second, we sought to more systematically define the principal components of phenotypic variation among these gastruloids. Among 88 gastruloids with ≥50 assigned cells, the first three pseudobulk transcriptional PCs explained two-thirds of the variance. PC1 was very strongly correlated with the undifferentiated PSC-like state fraction (35.8% of variance; *r* = −0.92; *p* = 1 x 10^−36^), PC2 with the number of cells (18.3% of variance; *r* = 0.61; *p =* 4 x 10^−10^), and PC3 with the hindbrain-like cell fractions (11.9% of variance; *r* = 0.58; *p* = 4 x 10^−9^) (Figure 4F; Figure S19B).

Are these phenotype-associated PCs correlated with the lineage relationships of founder mESCs? Phylogenetically “nearest neighbor” gastruloids showed modestly strong correlations for both PC1 (*r* = 0.30; *p* = 1 x 10^−3^) and PC3 (*r* = 0.32; *p* = 5 x 10^−4^), but not PC2 (Figure 4G; Figure S19C). Strong contributions came from Clade A (PSC-rich) and Clade F (hindbrain-rich) (Figure 4G). Of particular note, four gastruloids that have among the highest proportion of hindbrain cells and correspondingly the highest PC3 values, P1-B5 (79%), P2-E3 (60%), P2-E1 (38%) and P1-C8 (70%), compose a single subclade on the phylogenetic tree (purple box in Figure 4B & G). The result suggests that the mESC at the root of this subclade was biased to give rise to hindbrain-rich gastruloids, a proclivity that was heritably maintained through final cell divisions of the first epoch, the dissociation and replating of the first epoch mESC aggregate, and the expansion of the second epoch mESC aggregates (Figure 4A).

To more generally test the correlation between founder mESC relatedness and gastruloid phenotype, we devised two metrics of relatedness across all 3,828 possible gastruloid pairs. First, we used the Euclidean distance between a pair of gastruloids in a 10-dimensional space defined by the aforementioned pseudobulked transcriptional PCA and observed a positive correlation (Spearman’s *rho* = 0.052; *p* = 1 x 10^−3^; Figure S19D). Second, we defined a 30-dimensional PCA space from all single cell transcriptional data from these 88 gastruloids and then calculated, for each pair of gastruloids, the average percentage of nearest neighbors (*k* = 15) of cells of one gastruloid that derive from the other gastruloid. Here we observe a negative correlation, *i.e.* phylogenetically close founder mESCs gave rise to gastruloids whose cells were more similar than expected by chance (Spearman’s *rho* = −0.094; *p* = 6 x 10^−9^; Figure S19E). Although these metrics are not entirely independent (Figure S19F), the pattern across a range of phylogenetic distances supports the conclusion that there are aspects of mESC heterogeneity that arise during the first epoch, transmit to the second epoch, and contribute to gastruloid heterogeneity. While lineage reconstruction errors such as gastruloid misassignments could inflate these correlations, trends and significance persisted after excluding nearest-neighbor gastruloid pairs (Figure S19G).

### Linking the molecular state of founder mESCs to gastruloid fate bias

To investigate whether specific molecular factors might shape founder fate bias, we performed a Rewind-inspired^41^ clonal tracking^42–45^ experiment. First, we bottlenecked a polyclonal, statically barcoded mESC population (Figure 1A) to fewer than 50 cells, then expanded these to generate an oligoclonal line, which we subjected to single-cell multiomic profiling. In parallel, we used this line to generate 107 monoclonal and 32 polyclonal gastruloids on which we performed scRNA-seq (Figure 5A; STAR Methods). Relative to the gastruloid founders of the two-epoch experiment (Figure 4A), these oligoclonal founders are expected to be more distantly related, potentially providing greater power to track the consequences of inter-clone heterogeneity. With the resulting data, we set out to ask three questions.

First, what are the characteristic fate biases of each mESC subclone? To answer this, we first used Debris-seq to group the 107 monoclonal gastruloids into clonal clusters. After excluding lower quality barcodes (Figure S20A–B), 100 monoclonal gastruloids grouped into 29 clusters (1-12 gastruloids/cluster; mean 57 barcodes/cluster; range 10-164) (Figure 5B) which we infer correspond to mESC subclones present at the bottleneck. Next, 56,796 annotated single-cell transcriptomes (Figure 5C) were assigned to clusters based on the static clonal barcodes, resulting in 26 clusters with ≥200 assigned cells (Figure 5D, top; mean 1,824; range 227-8,100). Clusters varied widely in cell type compositions; some were mixed (*e.g.* cluster 29), others were strongly fate-biased (*e.g.* cluster 21 → hindbrain; cluster 9 → somites; cluster 8 → PSC-like) (Figure S20C). We grouped the 26 subclones into four fate-bias categories: neuroectodermal (n = 5), mesodermal (n = 4), PSC-like (n = 4), and mixed (n = 13) (Figure 5E–F).

Second, do polyclonal gastruloids exhibit the same diversity and inter-individual heterogeneity as monoclonal gastruloids? They do not. Despite deriving from the same bottlenecked mESC founders, polyclonal gastruloids were characterized by substantially lower cell type diversity. Posterior lineages dominated, as NMPs, spinal cord/r7/r8, and somites together comprised 88% of cells, while PSC-like and hindbrain lineages together accounted for only 5% (Figure 5C; Figure S20D). After CMO demultiplexing, 11,620 polyclonal cells (43% of total) were assigned to individual gastruloids, of which 20 had ≥200 cells (mean 535; range 220-1,291). In contrast with monoclonal gastruloids, polyclonal gastruloids had similar cell type compositions (Figure S20E). Strikingly, 82% of clone-assignable cells from polyclonal gastruloids derived from a single founder, clone 29, and for 14/20 of these, their proportion was >95% (Figure 5G–H). With the caveat that our clone-assignment rate was much lower for cells from polyclonal (14%) than monoclonal (83%) gastruloids, this observation mirrors what we observe in polyclonal mESC-derived embryoid bodies^27^, and suggests that the relative homogeneity of polyclonal gastruloids here may be a consequence of “clonal dominance” by a subclone exhibiting a mixed fate bias.

Third, can the fate biases of mESC subclones be traced to specific molecular factors? To answer this, we examined the multiomic profiles of 63,889 post-bottleneck mESCs that passed filtering on both modalities, of which 25,366 could be assigned via their barcodes to the same 26 monoclonal gastruloid-associated clusters described above (mean 976; range 99-5,526) (Figure 5D, bottom). Cells from fate-biased clusters showed no obvious stratification in UMAPs of either chromatin accessibility or gene expression (Figure 5I). However, gene-wise and motif-wise comparisons of neuroectodermally-, mesodermally-, and PSC-biased subclones against a shared reference of mixed-biased subclones revealed clear and statistically robust differences.

Most strikingly, PSC-biased subclones exhibited markedly reduced *Tet1* expression (log2FC −0.50; adjusted p = 1e-18), downregulation of the maternally-expressed Dlk1-Dio3 imprinted lncRNAs *Meg3* and *Rian* (log2FC −0.50 and −0.58; adjusted p ≤ 4e-6), with the same trend for *Mirg*, and upregulation of the separately imprinted, paternally-expressed gene *Peg10* (log2FC +0.68; adjusted p = 1e-4), with the same trend for *Plagl1* (Figure 5J; Table S5). Silencing of the Dlk1-Dio3 locus is an established marker of compromised developmental potency in mouse PSCs^46,47^, and Tet1, a 5-methylcytosine dioxygenase that protects imprinted loci from aberrant methylation, has been linked to its regulation^48,49^. The leading differential gene expression correlates of other fate biases were *Nedd4* (↓ neuroectodermal; log2FC −0.15; adjusted p = 3e-4) and *Meis2* (↑ mesodermal; log2FC +0.81; adjusted p = 8e-3). At the chromatin level, differences in TF motif accessibility were subtle (most |log2FC| < 0.1) but significant, including Nr5a2 and Esrrg (↓ in both mesodermal and PSC subclones; adjusted p ≤ 3e-6), CTCF (↑ mesodermal; adjusted p = 3e-5), NFYA (↓ neuroectodermal; adjusted p = 1e-3) (Figure 5K; Table S6). Overall, these results identify specific molecular factors and regulatory states of mESCs that are predictive of the fate biases that they will exhibit upon gastruloid induction. We emphasize that this heterogeneity is cryptic, in that it is not evident in unsupervised representations of mESC expression or chromatin state (Figure 5I), and emerged only once each mESC subclone was linked to the cell type compositions of its descendant gastruloids.

## DISCUSSION

Our study had two goals. First, to explore the origins of intra-batch, inter-individual heterogeneity in organoids; and second, to establish the DNA Typewriter lineage tracing system^28^ in mammalian stem cells. To these ends, we developed an *in vitro* system in which a single mESC founds a monoclonal gastruloid.

For the first goal, we were inspired by Luria and Delbrück’s classic fluctuation experiment^50^, *i.e.* whether variation pre-exists a stimulus and is heritable, versus being induced by it or arising stochastically afterward. Our findings suggest that the compositional heterogeneity of gastruloids reflects pre-stimulus factors, including heterogeneity among individual stem cells within a clonal line that can arise at discrete cell divisions. This parallels recent work by Shaffer, Raj, and colleagues on heritable clonal memory in cancer cells^51,52^, where heritable cell-state fluctuations contribute to divergent outcomes under a common stimulus.

Our intra-gastruloid findings (Figure 3) align with recent work that linked fate trajectories in gastruloid-like models to spatial positioning, signaling exposure, and cell rearrangements^23,26,53^. Collectively, these studies suggest that fluctuations in stem cell states, such as Nodal/BMP signaling, drive physical rearrangements that ultimately shape the emergence of the anterior-posterior axis. However, the use of polyclonal aggregates precluded these studies from determining when such fluctuations arise or how heritable they are across cell divisions. Leveraging DNA Typewriter in monoclonal gastruloids, our results suggest that mESC state fluctuations can be both sharp and heritable. For instance, in the Well16 gastruloid (Figure 3C), we infer that the first cell division produced two daughter lineages, one maintaining pluripotency, while the other underwent primitive streak-like differentiation. These differences may persist over multiple generations, influencing fate decisions, although lineage relationships could also reflect their spatial positions.

Our two-epoch experiment addressed this spatial confounding (Figure 4). Although the observed effects were more subtle, the data support the notion that founder mESC states influence gastruloid outcomes. Interestingly, this experiment exhibited less overall heterogeneity than our earlier experiments, likely due to the tight lineage relationships among founder mESCs, which we controlled by limiting the number of cell divisions in the first epoch. Nonetheless, principal components captured the representation of particular cell types (PSC-like cells, hindbrain) and gastruloid size. Gastruloids originating from closely related founder mESCs exhibited correlated cell type compositions, reinforcing the idea that lineage-dependent fate biases emerge long before differentiation cues are introduced.

What molecular differences distinguish founders that diverge? Our Rewind-inspired^41^ clonal tracking^42–45^ experiment points to imprinted, methylation-sensitive loci linked to developmental potency. Specifically, PSC-biased oligoclones showed reduced *Tet1* expression together with silencing of the Dlk1-Dio3 locus (Figure 5). Because this founder-level heterogeneity resembles the fate switches we observe at discrete cell divisions within reconstructed lineages (Figure 3), we speculate that the two reflect the same underlying phenomenon. For example, stochastic switching of *Tet1* expression at a single division may explain why one daughter clade becomes PSC-biased while its sibling does not (Figure 3C). In gastruloids from Wells 01 and 28, we can even infer directionality: a PSC-biased subclade arises within an otherwise diverse gastruloid, implying a stochastic loss of *Tet1* expression against a *Tet1*-expressing background. We are struck that these founders appeared essentially homogeneous by standard analysis (Figure 5I), and that these differences emerged as predictive only once each oligoclone was linked to the fates of its descendants. Latent, fate-relevant heterogeneity may therefore be pervasive but easily missed in conventional single-cell profiling, which is typically sparse and captures only a static snapshot.

Our results support the view that intrinsic cellular heterogeneity is not simply noise, but may be functionally important, such as ensuring efficient gastruloid formation^54^. Strikingly, our monoclonal gastruloids collectively access a much broader range of cell types than conventional polyclonal gastruloids, while our internally controlled comparison suggests that the apparent “buffering” of polyclonal aggregates reflects the dominance of particular clones, which narrows the realized set of fates relative to those latent in the founding population. This offers a potential explanation for inter-gastruloid heterogeneity^34^, *e.g.* mesodermally-biased subclones may stochastically come to dominate in some polyclonal gastruloids, and neuroectodermally-biased subclones in others. Our findings also complement recent molecular recording studies that investigated the fate distributions of NMPs in embryoids^53^, as here we focus on an earlier level, *i.e.* heritable biases among the founder mESCs themselves, prior to induction.

In stem cell biology, shifting from polyclonal to monoclonal stem cell aggregates as the starting point for organoid differentiation protocols may offer distinct advantages for certain applications. For example, in a companion study^27^, we show that cellular bottlenecks in polyclonal embryoid bodies (EBs) drastically reduce clonal complexity, confounding mosaic genetic screens, whereas monoclonal EBs offer the possibility of generating genetically homogeneous “individuals”. The establishment of additional protocols for deriving monoclonal organoids of various kinds could help to mitigate inter-individual heterogeneity or, alternatively, to harness it for specific research goals^27^.

For our second goal, we demonstrate that DNA Typewriter can reconstruct large-scale phylogenies from a single founder to tens of thousands of differentiated cells within a single experiment. Future enhancements, such as ENGRAM-based decorations and improved cell recovery, will refine both resolution and interpretability, and computational tools must evolve to fully leverage large-scale time-calibrated phylogenies at the resolution of millions to billions of cells. Our implementation in monoclonal gastruloids also lays the foundation for extending these approaches to *in vivo* models such as the mouse, a system wherein each individual also begins from a single stem cell^55^. Separately, although used here solely for gastruloid assignment, Debris-seq may carry information about which cell types are shed, and when. Here we find that shed-DTT abundance is structured by lineage (Pagel’s λ = 0.52-0.91 across eight gastruloids, median 0.74; all significant); a single-cell adaptation with serial sampling could relate detached, outcompeted, or apoptotic cells to the lineage tree.

Many questions remain. Is clonal dominance, observed both here and in embryoid bodies^27^, a general feature of polyclonally derived stem-cell models, and if so, does it arise through faster proliferation, active suppression, or both? How does it relate to the stochastic dominance of subclones in *in vivo* settings, *e.g.* mouse chimeras and lineage-traced zebrafish^56^? Might spontaneous heterogeneity among mESCs be amplified into divergent fates, providing a route to cell-type diversification under uniform signaling? We anticipate that fully addressing such questions will require reconstructing the joint lineage and signaling histories of many organoids and embryos, in sufficient numbers to support a statistical treatment of the underlying phenomena^57^.

### Limitations of the Study.

Several limitations of this work merit emphasis. First, the contributions of lineage histories and spatial positions were not fully disentangled in our experiments. Although the two-epoch design addresses this in part, concurrent measurement of both modalities (lineage and spatial) may be necessary for fully resolving their relative contributions to the observed fate biases. Second, DNA Typewriter-based lineage reconstruction is imperfect. Here, deep nodes are well supported but shallow nodes less so as editing saturates, such that the interpretation of fine-scale lineage relationships warrants more caution than the major-clade structure on which our conclusions rest. Third, all of our experiments began from a single starting condition (serum + LIF on MEFs) and a single mESC line (E14TG2a). Because the pluripotent state of mESCs prior to aggregation can itself bias gastruloid composition and variability^24,25^, our chosen condition likely shaped both the character and extent of the heterogeneity we observe. Fourth and relatedly, the engineering required for lineage recording and clonal tracking involved prolonged culture of the parental E14TG2a line, genomic integration of the recorder cassettes, and bottlenecking (to monoclonality for lineage reconstruction, and to an oligoclonal pool for clonal tracking), any of which may have affected developmental potential. Further work is necessary to evaluate whether other starting conditions or mESC lines, including the unmodified parental E14TG2a line, yield similar or different results. Accordingly, our results are best interpreted as establishing heritable, molecularly correlated fate biases within this engineered line, rather than as established properties of unmodified parental mESCs. Fifth and finally, mESC-derived gastruloids recapitulate only certain aspects of embryogenesis, and gastruloid formation is induced by an artificial *in vitro* process that relies on a defined culture medium (NDiff227 with a CHIR pulse) rather than the signaling environment of the embryo. The extent to which these findings generalize to other stem cell models or the *in vivo* setting, where the balance of heritable progenitor state vs. environmental cues may differ, remains to be investigated.

## RESOURCE AVAILABILITY

### Lead Contact

Correspondence and requests for materials should be addressed to Jay Shendure (shendure@uw.edu ).

### Materials Availability

Reagents generated in this study are available from the lead contact.

## STAR METHODS

### EXPERIMENTAL MODEL AND STUDY PARTICIPANT DETAILS

#### Mouse embryonic stem cells (mESCs) and culture

We adopted a standard protocol for culturing mESCs, similar to the protocol described in ref ^58^. Our mESCs (E14TG2a) were a gift from C. Schröter. During regular passaging and expansion, mESCs were initially cultured on a gelatin-coated plate using 2i-LIF medium, which is NDiff227 (commercially available N2B27-like medium; Takara) supplemented with 3 μM CHIR99021 (CHIR, Selleck, S2924), 1 μM PD0325901 (Selleck, S1036), and 1,000 units of ESGRO recombinant mouse LIF protein (Sigma-Aldrich, ESG1107). Gelatin-coated plates were prepared by making 0.2% (w/v) gelatin solution (Sigma, G1393) and autoclaving the bottle, which was applied to each culturing well (1 mL per well within a 6-well culturing plate) and incubated in a tissue culture incubator set to 37 °C with 5% CO2 for at least 30 minutes. After the coating, the gelatin solution was aspirated from the plate immediately before depositing mESCs. Cells are grown in the stem-cell-designated incubator (set to 37 °C with 5% CO2) and biosafety cabinet to avoid cross-contamination. After the initial expansion, mESCs were further adapted to the Serum-based medium, which is GMEM (Gibco) supplemented with 8% Fetal Bovine Serum (FBS, Biowest), 8% KnockOut Serum Replacement (KSR, Gibco), 1X Glutamax (Gibco), 1X MEM non-essential amino acids (Gibco), 1 mM Sodium Pyruvate (Gibco), and 0.1 mM beta-mercaptoethanol (BME, Gibco).

#### Mouse embryonic fibroblast (MEF) feeder cells and culture

Mito-C inactivated MEFs (CF-1 MEFs, Applied StemCell) were cultured in 6-well plates with DMEM medium with high glucose (Gibco), supplemented with 15% FBS (Biowest), 1X Glutamax (Gibco), and 1X MEM non-essential amino acids (Gibco), and used as feeder layers to support mESC colony formation.

### METHOD DETAILS

#### Monoclonal gastruloid induction protocol

To induce monoclonal gastruloids, we perform a two-stage protocol, where the first stage generates a monoclonal mESC cell aggregate (also referred to as spheroid culture), and the second stage closely mirrors the standard gastruloid induction protocol with a 24-hr CHIR pulsing.

The protocol starts by culturing Mito-C inactivated MEFs (CF-1 MEFs, Applied StemCell) in 6-well plates with DMEM medium with high glucose (Gibco), supplemented with 15% FBS (Biowest), 1X Glutamax (Gibco), and 1X MEM non-essential amino acids (Gibco). On the next day (Day 1), we flow-sort mESCs to ensure single-cell resuspension and add them to each 6-well plate at a low cell concentration of around 600 cells per 6-well. Cells are grown in the standard mESC media for an additional five days, where we expect to observe cell colonies of 100- to 150-μm diameter (estimated to be nearly 300 cells to match the number of deposited cells in the standard gastruloid protocol) that are isolated from the other mESC colonies. On Day 6, the MEF layer is selectively dissociated while keeping mESC colonies intact by washing each 6-well with 2 mL of PBS without calcium or magnesium twice, and the addition of 2 mL of Collagenase IV (1 mg/mL, StemCell Technologies) and incubation at 37 °C for 20-30 minutes. Once colonies are successfully lifted off of the MEF layer, they are carefully pooled into a 15-mL tube and allowed to settle by gravity. The remaining Collagenase IV solutions are carefully aspirated, and monoclonal colonies are washed with serum-containing mESC media (to inactivate any remaining Collagenase IV and to wash remaining MEFs away). Colonies are then transferred to a non-adherent 10-cm plate in the differentiation medium (NDiff227, Takara). After 24 hours, 3D spheroids or aggregates are manually picked into 96-well plates (non-adherent, round bottom) and are subsequently used downstream for the gastruloid induction protocol.

For the second stage, after each aggregate is placed into a well in a 96-well plate, they are treated as gastruloids of the 24-hour-after-aggregation time point in the conventional mouse 3D gastruloid induction protocol^58^ (Figure S1A). One day after culturing in the 96-well plate (Day 8, with cell numbers estimated to be 400-700 in each aggregate), culturing media was replaced by NDiff227 supplemented with 3 μM CHIR for the next 24 hours, matching the CHIR treatment between the 48- and 72-hour timepoints in the conventional gastruloid induction protocol. After the 24-hour CHIR pulsing, the culturing medium is replaced with the original NDiff227 medium until the harvesting of gastruloids for single-cell profiling.

#### Transduction of mESCs with lentiviral barcodes for assessing monoclonality

For the experiment reported in Figure S1A, we used lentiviral transduction to stably integrate a degenerate 8-mer barcode (NNNNNNNN) to mESCs. We used ViraPower Lentiviral Expression System (ThermoFisher) to generate lentivirus packaged with our construct encoding GFP-P2A-Puromycin resistance gene (PuroR) with the random 8-mer DNA barcodes positioned in the 3’-UTR of GFP transcript. The resulting lentivirus was concentrated overnight using Peg-it Virus Precipitation Solution (SBI) then aliquoted and flash-frozen using liquid nitrogen. Viral titers were generated using established protocols, where an aliquot of frozen virus was thawed and applied to mESCs at different dilutions (200 μL of diluted lentivirus solution to roughly 500,000 cells per well within a 6-well culturing plate). GFP fluorescence was used as a proxy for MOI efficiency, which was measured with FACS analysis. After the determination of low MOI conditions, cells were selected on puromycin to retain a polyclonal mESC population with cells harboring a unique static 8-mer barcode.

Monoclonal aggregates derived from 8-mer-barcoded mESCs were transferred individually to wells of 96-well plates and cultured through gastruloid induction as described above. Genomic DNA was extracted from each of 234 wells (ARCTURUS PicoPure DNA Extraction Kit, ThermoFisher), and the integrated static barcode amplified in a two-step indexed PCR using primers flanking the lentiviral insert, with the second step adding Illumina adaptors and dual index sequences for demultiplexing. Libraries were pooled, bead-cleaned (AMPure XP, Beckman Coulter), and sequenced on an Illumina NextSeq 2000. Reads were demultiplexed by index, and wells assigned ≥6,000 reads were retained for analysis (201/234; Figure S1C). For each retained well, reads were tabulated by barcode sequence and the well scored as monoclonal when the single most abundant barcode accounted for ≥90% of reads.

#### Generation of polyclonal DNA Typewriter lineage tracing cell line

For experiments reported in Figure 1 onwards, we generated DNA Typewriter lineage-tracing mESC lines via sequential integrations of piggyBac transposons, with each integration followed by selection steps. First, we generated PB-TAPE (Figure S1F), which includes two gene expression cassettes: 1) TAPE-targeting epegRNAs (random NNNGGA insertions) expressed from U6 promoter, and 2) GFP encoding DNA Tape in its 3’-UTR. DNA Tape is structured to include the T7 promoter sequence, a 12-bp Tape-specific degenerate barcode (TapeBC, NNNNNAANNNNN), 6xTAPE capable of six sequential insertions, and 10X Genomics Capture Sequence 1 (CS1) to aid recovery of DNA Tape during scRNA-seq. We also cloned PB-iPEmax-HygroR (Figure S1F), which encodes Doxycycline-inducible PEmax-P2A-mCherry and rtTA3-P2A-HygroR (reverse-tetracycline transactivator 3 and Hygromycin-resistance gene linked by P2A ribosomal skipping peptide sequence).

Next, we transfected PB-TAPE with the Super piggyBac transposase expression vector (SBI) for stable integration. We mixed PB-TAPE, PB-PuroR, and piggyBac transposase plasmids with the 90:5:5 mass ratio, then used Lipofectamine 2000 (ThermoFisher) protocol to transfect mESCs (4 μg per 1 million cells in 6-well plate). Transfected cells were cultured for another 7 days, and placed under puromycin selection (1 μg/mL) for two days. The resulting cells were mostly GFP-positive when imaged under the fluorescent microscope, possibly due to the high copy number of PB-TAPE integrants in each cell.

Finally, we performed another transfection of PB-TAPE, PB-iPEmax-HygroR, and piggyBac transposase, mixed at the 85:10:5 mass ratio. The resulting cells were cultured for another 7 days, selected using hygromycin (150 μg/mL) for four days, and then frozen.

#### sci-RNA-seq3 monoclonal gastruloid induction protocol

Polyclonal DNA Typewriter lineage tracing cells were thawed and passaged for at least a week. Recovered cells were FACS-sorted for GFP-positivity, with the upper 50% retained. Doxycycline (100 ng/mL) was added to the cell culture media a day before the sorting, and omitted in the cell culture after the seeding. Sorted cells were resuspended as single cells, diluted to 600 cells per 2 mL in Serum-medium, and plated on three 6-well plates containing the MEF cells, following the monoclonal gastruloid induction protocol described above. After the monoclonal colony separation step and formation of a spherical cell aggregate each from monoclonal mESC colony, we picked 144 cell aggregates into one-and-a-half 96-well plates. Gastruloids were grown for another three days after CHIR treatment. At the time of gastruloid harvesting, the culturing media from each well containing a gastruloid was collected for the Debris-seq protocol described below.

#### Data generation and processing for the sci-RNA-seq3 experiment

For the application of sci-RNA-seq3 to mouse gastruloids, we used an adaptation of the optimized sci-RNA-seq3 protocol that includes a T7 transcription step^29,30,59^. 144 monoclonal gastruloids collected in three pools (each containing 48 gastruloids) were gently resuspended in 2 mL lysis buffer to free the nuclei and fixed with methanol (80% methanol in a total of 10ml) for storage at −80 °C with at least 2 million nuclei per pool. Samples were thawed on ice and 10 mL of SPBSTM (sucrose, PBS, triton, magnesium chloride) buffer plus 100 μL DEPC (Millipore Sigma) was added to rehydrate the nuclei. Nuclei were centrifuged at 500 xg for 3 minutes at 4 °C and resuspended in 60 μL SPBSTM for the T7 reaction (NEB HiScribe kit #E2050S), adding 75 μL of the NTP/buffer mix and 15 μL of the enzyme mix. The nuclei were incubated at 37 °C for 30 minutes, then the volume was brought up to 1 mL with SPBSTM plus 50 μL 100 mM BS3 to fix the nuclei again, 10 minutes on ice, then centrifuged. Nuclei were resuspended at 2 million per 500 μL, and 56 μL of dNTPs were added and then distributed to a 96-well plate for reverse transcription. A separate sci-RNA-seq3 experiment was conducted for each of the three pools. Two types of indexed reverse transcription primers were used in each well: an oligo-dT primer for the transcriptome, and another for the DTT construct. After reverse transcription, nuclei were pooled and redistributed to a second plate to attach a second index by ligation. Then nuclei were pooled and redistributed again into their final plates (six plates in total, where two plates were prepared from each pool containing 48 gastruloids) for second-strand synthesis. The nuclei were dissolved with protease and then split: 5 μL of 10 mM Tris was added to each well and then immediately removed to a second plate to divide each well into two. One half was taken through the remaining sci-RNA-seq3 protocol with tagmentation and PCR for the transcriptome. The other half was taken directly to a PCR specific to the DTT construct, using the normal sci-RNA-seq3 P5 indexed primer on one side and a custom P7 primer (CAAGCAGAAGACGGCATACGAGATNNNNNNNNNNGGTACCATAGCAGATGATCCATGGTC) on the other side, to specifically enrich for the DTTs. PCRs for the transcriptome and enrichment were sequenced independently on an Illumina Nextseq 2000.

Transcriptome data from each of the six individual plates were processed independently. For each plate, read alignment and gene count matrix generation were performed using the pipeline that we developed for sci-RNA-seq3 (https://github.com/JunyueC/sci-RNA-seq3_pipeline ). Briefly, base calls were converted to FASTQ format using Illumina’s *bcl2fastq*/v2.20 and demultiplexed based on PCR i5 and i7 barcodes using maximum likelihood demultiplexing package *deML*^60^ with default settings. Demultiplexed reads were filtered based on the reverse transcription (RT) index and hairpin ligation adaptor index (Levenshtein edit distance (ED) < 2, including insertions and deletions) and adaptor-clipped using *trim_galore*/v0.6.5 (https://github.com/FelixKrueger/TrimGalore ) with default settings. Trimmed reads were aligned to a custom mouse reference genome (GRCm39 with an additional DTT sequence) using *STAR*/v2.6.1d^61^ with default settings and gene annotations from GENCODE VM31. Mapped reads were extracted, and duplicates were removed using the unique molecular identifier (UMI) sequence, RT index, ligation index, and read 2 end-coordinate (that is, reads with identical UMI, RT index, ligation index, and tagmentation site were considered duplicates). Finally, mapped reads were split into constituent cellular indices by further demultiplexing reads using the RT index and ligation index. To generate digital expression matrices, we calculated the number of strand-specific UMIs for each cell mapping to the exonic and intronic regions of each gene with the *python*/v2.7.13 *HTseq* package^62^. For multi-mapping reads (*i.e.* those mapping to multiple genes), the reads were assigned to the gene for which the distance between the mapped location and the 3’ end of that gene was smallest, except in cases where the read mapped to within 100 bp of the 3’ end of more than one gene, in which case the read was discarded. For most analyses, we included both expected-strand intronic and exonic UMIs in per-gene single-cell expression matrices. After the single-cell gene count matrix was generated, cells with low quality (UMI < 200 or detected genes < 100 or unmatched_rate (proportion of reads not mapping to any exon or intron) ≥ 0.4) were filtered out.

We performed two steps to exhaustively detect and remove potential doublets. Of note, all these analyses were performed separately on data from each plate. First, we applied the *scrublet*/v0.1^63^ pipeline to each dataset with parameters (min_count = 3, min_cells = 3, vscore_percentile = 85, n_pc = 30, expected_doublet_rate = 0.06, sim_doublet_ratio = 2, n_neighbors = 30, scaling_method = ’log’) for doublet score calculation. Cells with doublet scores over 0.2 were annotated as detected doublets. Second, we performed two rounds of clustering and used the doublet annotations to identify subclusters that are enriched in doublets. The clustering was performed based on *Scanpy*/v.1.6.0^64^. Briefly, gene counts mapping to sex chromosomes were removed, and genes with zero counts were filtered out. Each cell was normalized by the total UMI count per cell, and the top 3,000 genes with the highest variance were selected, followed by renormalizing the gene expression matrix. The data was log-transformed after adding a pseudocount and scaled to unit variance and zero mean. The dimensionality of the data was reduced by PCA (30 components), followed by Louvain clustering with default parameters (resolution = 1). For the Louvain clustering, we first computed a neighborhood graph using a local neighborhood number of 50 using *scanpy.pp.neighbors*. We then clustered the cells into sub-groups using the Louvain algorithm implemented by the *scanpy.tl.louvain* function. For each cell cluster, we applied the same strategies to identify subclusters, except that we set resolution = 3 for Louvain clustering. Subclusters with a detected doublet ratio (by *Scrublet*) over 15% were annotated as doublet-derived subclusters. We then removed cells which are either labeled as doublets by *Scrublet* or that were included in doublet-derived subclusters. Altogether, 7.4% to 11.1% of cells in each plate were removed by this procedure.

After removing the potential doublets detected by the above two steps, we further filtered out the potential low-quality cells with the doublet score (by *Scrublet*) greater than 0.15, the percentage of reads mapping to ribosomal chromosomes (Ribo%) > 5, the percentage of reads mapping to exonic regions (exon%) > 85, or the percentage of reads mapping to mitochondrial chromosomes (Mito%) > 10. Subsequently, we determined thresholds for UMI counts per cell used for quality filtering, set at the mean plus 2 standard deviations and mean minus 1.5 standard deviations of log2-scaled values. Cells with fewer than 250 detected UMIs were further excluded. Following this, we merged cells from individual plates to generate the final dataset, composed of 247,064 cells. The median UMI count per cell was 609, with a median gene count detection per cell of 512.

We then applied *Seurat/v3*^65^ to this final dataset, performing conventional single-cell RNA-seq data processing: 1) retaining protein-coding genes, lincRNA, and pseudogenes for each cell and removing gene counts mapping to sex chromosomes; 2) normalizing the UMI counts by the total count per cell followed by log-transformation; 3) selecting the 2,500 most highly variable genes and scaling the expression of each to zero mean and unit variance; 4) applying PCA and then using the top 30 PCs to calculate a neighborhood graph, followed by louvain clustering (resolution = 1); 4) performing UMAP visualization in 2D space (min.dist = 0.3). For cell clustering, we manually adjusted the resolution parameter towards modest over-clustering, and then manually merged adjacent clusters if they had a limited number of differentially expressed genes (DEGs) relative to one another or if they both highly expressed the same literature-nominated marker genes. Subsequently, we annotated individual cell clusters using at least two literature-nominated marker genes per cell type label (Table S1).

DTT data from six individual plates (two plates per a pool of 48 gastruloids) were processed separately using the same pipeline: base calls were converted to FASTQ, demultiplexed, filtered by RT and hairpin ligation indices, trimmed, and aligned to a custom mouse genome (GRCm39 + DTT). Mapped reads were extracted for analysis.

#### Debris-seq data generation and processing

Cell culture medium from each well containing a monoclonal cell aggregate was collected to purify genomic DNA from cell debris (Debris-seq). Each well was carefully pipet-mixed to resuspend any settled cell debris without disturbing the cell aggregates before removing the culturing medium. The culturing medium containing cell debris was transferred to a PCR tube strip containing 8 tubes and spun down using the table-top microfuge for 20 minutes at around 2000 xg (MyFuge, Benchmark). The culturing medium was carefully removed without disturbing the cell-debris pellet. The genomic DNA from each cell-debris pellet was purified using the DNA extraction kit (ARCTURUS PicoPure DNA Extraction Kit, ThermoFisher), where each pellet was resuspended in 10 to 15 μL of DNA extraction buffer. The DNA Tape sequences within the resulting genomic DNA were PCR-amplified, as described previously^28,66^. Briefly, about 2 μL of the genomic DNA solution was added to 50 μL of the PCR reaction (KAPA2G Robust HotStart ReadyMix), where PCR primers (0.4 μM final concentrations) added Illumina sequencing primer binding sequences. PCR reactions were performed for 3 minutes at 95 °C; 15 seconds at 95 °C, 10 seconds at 65 °C and 40 seconds at 72 °C for 30 cycles; and 1 minute at 72 °C. A portion of the resulting PCR reactions (1 μL) is then used for the subsequent PCR reaction (25 μL), which uses primers that add Illumina sequencing adaptors and dual-index sequences for the future demultiplexing of samples from the sequencing run. The same PCR reaction parameters were used, except for the reduction of cycling numbers to 6 cycles in total. The resulting libraries were pooled, cleaned up using magnetic beads (AMPure XP, Beckman Coulter), and sequenced on Illumina Nextseq2000.

The resulting sequencing runs were demultiplexed by their indices, generating a single FASTQ file per aggregate-bearing well. FASTQ files were analyzed using a custom Python script that performed the following three steps: 1) For each sequencing read, it extracted TapeBC and DNA Typewriter Tape Site1-6 insertions (NNNGGA) based on their amplicon positions. The script goes through Site1 to Site6 sequentially, checking for insertions from the first site (“InsertBC(1)”) to the last (sixth) site (“InsertBC(6)”) on Tape. 2) Reads with the same TapeBC and editing patterns on InsertBC(1–6) are tabulated as a unique TapeBC-edit pattern and its frequency. 3) TapeBC and the InsertBC(1) are combined (e.g., TTTTTAAGCAAT-CCTGGA, where the first 12-bp is the NNNNNAANNNNN TapeBC and the second 6-bp is the insertion detected in the first site), and only the most frequent insertion pattern (single Site1–6 insertion pattern per a unique TapeBC-InsertBC(1) detected) is kept in the final output, with an additional threshold of read frequency to remove any combinations that are observed less than 0.2% of the total read. The resulting file includes the well position in the first column, TapeBC-InsertBC(1) in the second column, Site1-Site6 in the following six columns, and the read number in the last column.

#### Lineage tracing monoclonal gastruloid induction protocol (8 gastruloids)

We generated a highly efficient DNA Typewriter lineage recording cell line by characterizing the recording efficiency of each monoclonal cell line. We started from the ongoing culture of the polyclonal DNA Typewriter lineage tracing mESC line (described above in the “Generation of polyclonal DNA Typewriter lineage tracing cell line” section), then used FACS to sort a high GFP positive population (1% GFP brightness). The resulting single-cell sorted cells were plated on the layer of MEF to grow monoclonal colonies, which were picked, individually dissociated, and plated into 36 wells in a 96-well plate coated with 0.1% Gelatin. They were expanded until they reached a confluent well in a 6-well, where each culture was split into three parts: 1) frozen to preserve, 2) cultured for another 2 days in a 96-well, and 3) cultured for 2 days in a 96-well in the presence of 100 ng/mL Doxycycline to induce genomic recording. Cultured cells were harvested after 2 days and lysed to collect genomic DNAs. DNA Tapes from genomic DNA samples were PCR-amplified and sequenced to quantify the editing with or without Doxycycline induction. Among 36 cell lines, we identified three (named Clone-05, Clone-25, and Clone-32) that had the highest editing efficiency with Doxycycline induction and minimal editing without the induction.

We used these three cell lines, mixed at equal numbers, to induce monoclonal cell lines for high-resolution lineage reconstruction. We added 100 ng/mL Doxycycline into the culturing medium throughout the monoclonal gastruloid induction protocol, starting a day before the single-cell sorting step. We diluted 3600 sorted cells to 12 mL of Serum-based medium, then distributed them across wells of a single 6-well plate containing MEF to support monoclonal colony formation. 32 monoclonal cell aggregates were picked into a 96-well plate, and treated with CHIR for 24 hours according to the monoclonal gastruloid induction protocol. A day after the CHIR-treatment step (equivalent to the 96-hr after-aggregation timepoint in the conventional gastruloid induction protocol), we collected cell culture medium from each well and performed Debris-seq to access the editing efficiencies within each cell aggregate. All aggregates showed relatively high editing efficiencies (34 ± 12 % of the third sites within the DNA Typewriter Tapes edited). Debris-seq also provided cell line information of each monoclonal gastruloid, by matching the recovered TapeBCs with previously profiled TapeBCs from each of the three cell lines. We used this information to select 8 gastruloids to profile using 2 lanes of 10X Genomics scRNA-seq kit (Well14, Well17, Well25 into the first reaction lane containing cells from Clone 05, and Well01, Well03, Well21, Well16, and Well28 into the second reaction lane containing cells from Clone 25 and 32), dissociated and processed following the conventional protocols (Cell Preparation guide, CG00053 Rev C, and User Guide for Chromium Next GEM Single Cell 3’ HT Reagent Kits v3.1, Rev D).

#### Data generation and processing for the “eight monoclonal gastruloids” experiment

The single-cell transcriptome data from each 10X Genomics scRNA-seq lane were processed separately using Cell Ranger 7.2.0^67^ with default settings (e.g., --include-introns true) and refdata-cellranger-mm10-3.0.0 as the reference. We extracted the gene-by-cell matrix from the ‘raw_feature_bc_matrix’ folder, filtered out cells with UMI counts below 500 or fewer than 250 detected genes, and retained genes from chromosomes 1-19, X, Y, and MT. We then detected doublets using the Scrublet/v0.1^63^ pipeline and calculated the percentage of reads mapping to either mitochondrial (i.e. MT%) or ribosomal chromosomes (i.e. Ribo%) for individual cells. After manually examining the distribution of UMIs and MT% across cells, we applied the following criteria to filter out potentially low-quality cells:

- Log2 UMI Counts: Excluded cells with counts below 12.5 in lane 1 or below 12 in lane 2, as well as those exceeding the top 0.5% of total cells.
- Doublet Scores: Removed cells with doublet scores above 0.2, as calculated by Scrublet.
- MT%: Excluded cells with MT% over 10% or below 1%.
- Ribo%: Removed cells with Ribo% over 40%.

After combining cells from two lanes, we applied Seurat/v5^68^ to this final dataset, performing conventional single-cell RNA-seq data processing: 1) retaining protein-coding genes and lincRNA for each cell and removing gene counts mapping to sex chromosomes; 2) normalizing the UMI counts by the total count per cell followed by log-transformation; 3) selecting the 2,500 most highly variable genes and scaling the expression of each to zero mean and unit variance; 4) applying PCA and then using the top 30 PCs to calculate a neighborhood graph, followed by louvain clustering; 5) performing UMAP visualization in 2D space (min.dist = 0.3). For cell clustering, we manually adjusted the resolution parameter towards modest over-clustering, and then manually merged adjacent clusters if they had a limited number of differentially expressed genes (DEGs) relative to one another or if they both highly expressed the same literature-nominated marker genes (Table S1). Subsequently, we annotated individual cell clusters using at least two literature-nominated marker genes per cell type label.

Next, we processed the sequencing reads of DTT captured in the same scRNA-seq experiment. We extracted cell-specific barcode (CellBC), UMI added during cDNA synthesis, TapeBC and 6 InsertBC from each DTT sequencing reads, removed reads with less than 2 UMI associated with particular CellBC-TapeBC-6xInsertBC combinations, removed reads without CellBC matching to ones recovered from the single-cell transcriptomic library, removed reads without TapeBC matching to previously characterized ones from three clonal cell lines after correcting a single base-pair mismatch. In our data set, we often observed recovery of multiple 6xInsertBC combinations per CellBC-TapeBC combination, which suggested the existence of multiple DTT integrants with the same TapeBC. This may have occurred due to insufficient complexity of TapeBC in the DTT library, transposase-mediated repeated integration during DNA replication, or duplication of ploidy within the mESC. To allow discernment of unique integration within the genome during “cell-to-well” assignments, we used the first InsertBC (InsertBC(1)) as part of the TapeBC that identified a unique integration. Insertions are identified by their expected position within the amplicon sequencing read and additional 1-nt in its 3’-end, and searching for GGAT sequences in the last three position of NNNGGA insertions and the next base T. In case there are multiple 6xInsertBC combinations assigned to each CellBC-TapeBC-InsertBC(1) combination, we chose the InsertBC(2-6) combination with most UMI and sequencing read counts.

From either Debris-seq or scRNA-seq data, we defined unique DTTs with “TapeBC + InsertBC(1)” and retained only the most abundant InsertBC(2)-(6) edit combination for each DTT within each well or cell. We assigned cells to specific wells/gastruloids by matching the DTTs detected in individual cells with those identified in individual wells using Debris-seq. The assignment strategy was generally consistent with our approach for mapping cells obtained from sci-RNA-seq3 to their gastruloid origins, with adjustments made to account for differences in cell number and sequence coverage:

1. We combined DTTs detected from eight wells across three clones in Debris-seq, retaining those that were either unique to a well or overlapped across multiple wells.
2. In the scRNA-seq dataset, we excluded cells with fewer than two detected DTTs or with a total DTT UMI count of less than 10.
3. For additional assignments, we performed dimensionality reduction on the cells using only the DTT matrix. For unassigned cells, we identified the wells of their top 10 nearest assigned neighbor cells in PCA space (n = 30) and assigned them if the largest overlap exceeded the second largest overlap by at least 50%.
4. A total of 8,545 cells were allocated to 8 gastruloids. On average, each gastruloid contained 1,068 cells, with a median of 809 cells, ranging from 312 to 2,696.

#### “Tree-of-trees” experiment

We performed a two-stage “tree-of-trees” experiment by inducing multiple monoclonal gastruloids from a single starting mESC. From the mESC culture (Clone-05), we used FACS to sort around 3,600 cells, which were plated across a 6-well plate containing MEFs. The serum-based medium contained Doxycycline (100 ng/mL) throughout the experiment, starting from a day before the cell sorting (D0). Five days after the cell plating (D6), nine monoclonal colonies were isolated from MEFs, and cultured in a non-adherent, round-bottom 96-well for each to form a spherical aggregate. On the next day (D7), the culturing medium from each well was collected for Debris-seq, and each cell aggregate was single-cell dissociated using Trypsin and plated into a culturing well with MEFs within a 6-well plate, totaling 9 wells across one-and-a-half 6-well plates. Based on the editing efficiency measured from the Debris-seq result, we selected one well for monoclonal colony isolations (D13). After culturing isolated monoclonal cell aggregates in a non-adherent 10-cm culturing plate to form spherical cell aggregates, we transferred 108 aggregates into two 96-well plates the next day (D14), followed by the CHIR treatment and the rest of the monoclonal gastruloid induction protocols. During the CHIR treatment, we noticed that we lost two gastruloids (second 96-well plate, well B10 and D6, or P2-B10 and P2-D6), possibly removed along with the culturing medium during the medium replacement. We collected culturing medium from each well for Debris-seq on D17 (including the missing well, which may contain cell debris), and harvested gastruloids on D18 (equivalent to a 144-hour after-aggregation time-point in the conventional gastruloid induction protocol) for the 10X Genomics scRNA-seq protocol.

#### Data generation and processing for the “tree-of-trees” experiment

We dissociated all 106 gastruloids and profiled them across eight 10X Genomics lanes (v3.1-HT). The single-cell transcriptome data from each lane were processed separately using Cell Ranger 7.2.0^67^ with default settings (e.g., --include-introns true) and refdata-cellranger-mm10-3.0.0 as the reference. We extracted the gene-by-cell matrix from the ‘raw_feature_bc_matrix’ folder, filtered out cells with UMI counts below 500 or fewer than 250 detected genes, and retained genes from chromosomes 1-19, X, Y, and MT. We then detected doublets using the Scrublet/v0.1^63^ pipeline and calculated the percentage of reads mapping to either mitochondrial (i.e. MT%) or ribosomal chromosomes (i.e. Ribo%) for individual cells. After manually examining the distribution of UMIs and MT% across cells, we applied the following criteria to filter out potentially low-quality cells:

- Log2 UMI Counts: Excluded cells with counts below 10.5 (or 11 in some lanes) or exceeding the top 0.5% of total cells.
- Doublet Scores: Removed cells with doublet scores above 0.2, as calculated by Scrublet.
- MT%: Excluded cells with MT% over 10% or below 1%.
- Ribo%: Removed cells with Ribo% over 40%.

After combining cells from eight lanes, we applied Seurat/v5^68^ to this final dataset, performing conventional single-cell RNA-seq data processing: 1) retaining protein-coding genes and lincRNA for each cell and removing gene counts mapping to sex chromosomes; 2) normalizing the UMI counts using the SCTransform function^69^ implemented in Seurat; 3) applying PCA and then using the top 30 PCs to calculate a neighborhood graph, followed by Louvain clustering; and 4) performing UMAP visualization in 2D space (min.dist = 0.3). For cell clustering, we manually adjusted the resolution parameter towards modest over-clustering, and then manually merged adjacent clusters if they had a limited number of differentially expressed genes (DEGs) relative to one another or if they both highly expressed the same literature-nominated marker genes (Table S1). Subsequently, we annotated individual cell clusters using at least two literature-nominated marker genes per cell type label.

#### Single cell molecular profiling of oligoclonal mESCs and derived gastruloids

We started from the polyclonal mESC line described in Figure 1A, in which each cell bears a unique repertoire of static barcodes on DNA Tapes. This population was bottlenecked to approximately 50 cells, and then expanded to establish an oligoclonal mESC line. Once the oligoclonal line reached several million cells (one confluent well of a 6-well plate), a portion was subjected to single-cell multiome profiling (4 reactions; 10x Genomics Chromium Next GEM Single Cell Multiome ATAC + Gene Expression), while the remainder was used for the induction of both polyclonal (n = 32; 5-day protocol) and monoclonal (n = 107 gastruloids; 11-day protocol) gastruloid induction, as described above. Polyclonal gastruloids were processed for scRNA-seq with Cell Multiplexing Oligo (CMO) labeling (10x Genomics 3′ CellPlex Kit). In brief, each gastruloid was dissociated and labeled with one of 8 CMOs, and batches of 8 CMO-labeled gastruloids were pooled for scRNA-seq (4 lanes; 10x Genomics v3.1 chemistry). Monoclonal gastruloids were harvested to a single pool for scRNA-seq (12 lanes; 10x Genomics v3.1 chemistry). During sample collection of monoclonal gastruloids, the medium from each well was retained and processed via Debris-seq (described above), including recovery of genomic DNA and amplicon sequencing of static barcodes in each well.

#### Multiome profiling and data analysis of oligoclonal mESCs

mESCs were profiled across four lanes using the 10x Multiome kit for concurrent profiling of gene expression and chromatin accessibility. Data from each lane was processed separately using cellranger-arc-2.0.2 with default settings and cellranger-arc-mm10-2020-A as the reference. We retained 63,889 cells passing quality filters on both modalities and performed the same cell-to-clone assignment described above for gastruloids, successfully assigning 25,366 cells to the same 26 clonal clusters identified from monoclonal gastruloids (mean 976 mESCs per cluster; range 99-5,526). TF activity was calculated from the scATAC-seq modality by multiplying the TF motif-by-peak (generated by default in the cellranger-arc pipeline via MOODS scanning of peaks against JASPAR position-weight matrices) and peak-by-cell matrices, yielding a per-cell measure of global TF activity. To identify molecular features associated with gastruloid fate bias, we tested for differential gene expression (16,605 genes) and differential TF motif activity (577 TFs) in founder mESCs, comparing each of the three fate-biased categories (PSC, neuroectodermal, and mesodermal) against the mixed fate-bias category. Gene expression or TF activity values were first normalized by total counts per cell and then natural-log scaled. Cells assigned to each of the three fate-biased categories were then compared against cells assigned to the mixed category using the FindMarkers function (Wilcoxon test) in Seurat/v5. To control the false discovery rate (FDR), the resulting unadjusted p-values were compared against a null distribution generated by randomly reshuffling the fate-bias labels of the 26 clonal clusters 10 times. Results with FDR < 0.05 are reported in Tables S5–S6, along with log2 fold-changes and other metrics.

### QUANTIFICATION AND STATISTICAL ANALYSIS

#### Mapping cells acquired from sci-RNA-seq3 to their gastruloid origins

We assigned each single-cell transcriptional profile to one of the 144 wells in which individual gastruloids were grown, as a proxy for their gastruloid-of-origin. Our heuristic for grouping/assigning cells had the following steps:

1. From the Debris-seq output, we defined unique DTTs using “TapeBC + InsertBC(1)” and retained only the most abundant combination of InsertBC(2)-(6) for each DTT within each well. We focused on 4,995 unique DTTs observed in Debris-seq data as confirmed candidates (*i.e.* “white list”), each exclusively appearing in one of 138 wells. The remaining 6 wells were excluded because they had no unique TapeBCs assigned.
2. In the sci-RNA-seq3 dataset, we excluded cells with fewer than three detected DTTs and then aligned the DTTs to the confirmed list from Debris-seq, allowing for a 1 bp mismatch on the 12-bp TapeBC while requiring exact matches on the first 4 editing sites. To account for potential differences in induction times between Debris-seq and scRNA-seq, we allowed ‘none’ editing on the 2nd, 3rd, and 4th editing sites of DTTs in the scRNA-seq to match any editing pattern in the DTTs from Debris-seq. This resulted in a matrix of DTTs by cells with read numbers as values. We excluded cells with fewer than three distinct DTTs detected, resulting in a final count of 154,988 cells and 4,609 DTTs.
3. We quantified the overlap between DTTs observed in cells vs. wells, and assigned 129,853 cells in cases where the largest overlap exceeded the second-largest overlap by at least 50%.
4. To make additional assignments, we performed principal components analysis (PCA)-based dimensionality reduction of scRNA-seq profiles based solely on DTTs matrix rather than transcriptional profiles. DTTs with less than 10 reads across all cells were filtered out. We applied *Seurat/v5*^68^ to normalize the read counts by the total count per cell, followed by log-transformation and scaling each DTT to have a zero mean and unit variance. Next, PCA was applied, and the top 100 PCs were selected to calculate a neighborhood graph. This was followed by UMAP visualization in a 2D space (min.dist = 0.1). For unassigned cells, we identified the wells of their top 10 nearest already-assigned neighbor cells in PCA space (n = 100), and assigned a further 19,363 cells in cases where the largest overlap exceeded the second largest overlap by at least 50%.
5. After filtering out gastruloids with fewer than 100 cells, a total of 148,724 cells (96% of 154,988 cells) were allocated to 121 gastruloids. On average, each gastruloid contained 1,229 cells, with a median of 1,066 cells. The maximum cell count reached 5,883.
6. We identified the DTTs detected in at least 5% of the assigned cells, and then evaluated Jaccard similarities between the DTTs of cells assigned to each gastruloid and those detected from Debris-seq for each gastruloid.

#### Testing whether the abundance of shed DTT in Debris-seq is structured by lineage

For each of the eight gastruloids, we identified DTTs (defined for our purposes here as the combination of barcode and the first four TAPE editing sites) shared between the Debris-seq and scRNA-seq datasets. We then constructed two matrices: (1) the read counts per DTT in the Debris-seq data, and (2) the DTT-by-cell assignment matrix from the scRNA-seq data, binarized and normalized by the total number of DTTs per cell. Multiplying these two matrices yielded a cell-level Debris-seq abundance score, reflecting the cumulative Debris-seq signal across all DTTs assigned to each cell. We then tested for phylogenetic signals in the cell-level Debris-seq abundances using a likelihood ratio test of Pagel’s λ, as implemented by phylosig(method = “lambda”, test = TRUE) in the *phytools* R package.

#### Inferring DTT integration events and edit patterns

A major challenge in analyzing DTT data is that TapeBCs do not uniquely define genomic loci, as most DTTs are duplicated, likely due to excision/reintegration events that occur before the transposase has diluted out. This duplication complicates lineage reconstruction, making it difficult to compare edit patterns across cells without knowing the originating DTT locus. However, because we simultaneously recorded cell barcode information alongside the expressed Tape sequences, we can detect these duplication events as co-expressed Tapes bearing different patterns of edits. For example, if a given cell has a sequential pattern of edits A–B–C for a particular TapeBC but also expresses the same TapeBC with a pattern D–E–F, that TapeBC must be associated with at least two genomic integrations of DNA Typewriter Tape.

To take advantage of this, we developed a method to infer the most likely number of integration events per TapeBC into the genome as well as the edit patterns that were generated from each integration locus, capturing the true sequence of events that occur from the starting cell at each locus independently. The key principle on which this method was based was the idea that in a given cell, for a given TapeBC, there should exist only one observed pattern of edits. If multiple patterns of edits with the same TapeBC in the same cell are observed, this is evidence for multiple genomic integration sites for that TapeBC and associated DTT.

1. Determining consensus patterns of edits: From the scRNA-seq DTT data, we generated a table where each row represents a unique sequence defined by the CellBC, TapeBC, six editing sites (InsertBC(1) to InsertBC(6)), and the UMI count for that exact sequence. We then filtered this table for a given TapeBC in order to track its patterns of edits across all cells. For each TapeBC across all cells, we computed a pairwise Hamming distance matrix for unique edit site sequences and grouped identical sequences into clusters if they contained more than 100 UMIs. This clustering step grouped unique sequences by exact matches, assigning them to a cluster only if the resulting cluster size exceeded 100 UMIs; this is analogous to a DBSCAN approach with a radius of 0. UMIs not assigned to any cluster were discarded as likely noise from PCR or sequencing errors. The consensus sequence for each cluster was then refined to include only edited regions by identifying the edit key “GGAT” every 7 bases. By pooling information across all cells, this approach searches for patterns of edits that recur throughout the dataset while allowing sequences generated by PCR or sequencing error to be readily identified and discarded.
2. Generating a graph of edit-pattern mutual exclusivity: After identifying consensus edit patterns, we generated a cell-by-pattern matrix with UMI counts, row-normalized, and log-transformed to highlight differences between cells. A binarization threshold was set at the lowest point between distribution modes, producing a more interpretable matrix indicating whether a pattern is present in each cell for a given TapeBC. This binarized matrix provides an intuitive representation of multiple integrations: if a cell has a row sum greater than 1 (i.e., more than one pattern of edits), it carries multiple integrations. Our goal, however, was not to infer per-cell integrations but rather to infer the Tape integration structure across all cells. For example, if pattern 1 and pattern 2 are co-expressed in the same cells, they must have been generated from different loci. We then clustered the binary matrix using DBSCAN, grouping cells with the same sets of expressed patterns. Cells not assigned to a cluster, typically due to dropout, were classified using KNN in normalized log-UMI space. Each cluster’s binary expression vector was then used to compute a mutual exclusivity matrix via cosine distance with itself, forming a graph where nodes represent edit patterns and edges indicate mutual exclusivity (meaning the patterns are not co-expressed in the same cells). The cosine metric was chosen because it draws an edge between two patterns only when a 1 is present for both patterns, rather than a 0 for both. We intersected these graphs across all clusters to create a final graph representing relationships between edit patterns across all cells, assuming that in a monoclonal experiment, co-expressed patterns in any cluster of cells must have originated from distinct loci. That is, if two patterns are co-expressed in at least one cluster, they are treated as mutually exclusive with respect to Tape locus in all cells.
3. Grouping patterns into loci: To group edit patterns into shared loci of origin, we used the mutual exclusivity graph to identify complete subgraphs (cliques) as putative integration events. The intuition is that if the graph were complete (fully connected), no pattern would ever be co-expressed, implying a single locus of origin for all patterns; more generally, a complete subgraph (clique) represents a putative locus, so the task becomes finding a node partition of the graph in which each induced subgraph is a clique. We searched for a clique partition that: 1) minimized the number of inferred integration events; and 2) maximized the sum of edge weights, which reflect shared consecutive edits between patterns- in other words, the most biologically parsimonious solution. Minimizing the number of cliques reflects the most likely explanation (the fewest integration events), while the edge weights, defined as the number of shared consecutive edits between patterns, break ties among otherwise-equivalent solutions by favoring cliques whose patterns are more related and therefore more likely to have arisen from the same locus. To achieve this, we applied graph coloring to the complement graph, using a greedy algorithm to assign nodes to cliques. Because exhaustive search for minimal clique partitions is intractable on large datasets, we inverted the problem: the complement of the graph can be colored with a minimal number of colors using greedy graph-coloring approaches, in which nodes are assigned colors such that no two neighboring nodes share the same color, and the resulting color assignments correspond directly to clique memberships in the original graph. By running this algorithm multiple times with randomized node orders, we selected the solution with the fewest cliques and the highest sum of edge weights. Each inferred clique represents a distinct integration locus for a given TapeBC, grouping edit patterns accordingly. This resulted in a final matrix where rows represent cells and columns correspond to inferred loci, with the detected patterns per cell stored in the columns according to the clique groupings, serving as the basis for calculating pairwise lineage distances between cells and, ultimately, for generating phylogenies with UPGMA.

#### Generating phylogenetic trees from DTT data

To reconstruct the phylogenetic tree from genomic recordings, we first filtered out DTTs found in fewer than 10 cells for each clone (Clone-05, Clone-25, and Clone-32). We then excluded cells with DTT counts outside the range of mean − 1.5 × SD to mean + 2 × SD. To calculate cell-cell distances within each clone, we retained DTTs observed in both cells and computed an edit distance for each DTT (each containing six edit sites) between the two cells. This distance represents the number of steps required to transform the DTT in one cell to match the other, including reversing edits back to unedited and then applying forward edits. For example, the distance between (A C D - - -) and (B - - - - -) is 4. The possible distances range from 0 to 12. The average edit distance across all shared DTTs between pairs of cells was calculated, followed by the generation of a phylogenetic tree using the UPGMA method, implemented in the ape package^70^ in R.

#### Bootstrap analysis

We conducted a bootstrapping analysis to assess the robustness of the trees for individual clones or gastruloids. For each of the 100 bootstraps, we resampled an equal number of DTTs (each containing six edit sites) with replacements to reconstruct a new tree. Clade concordance was evaluated by comparing each bootstrap tree to the original and calculating the transfer bootstrap expectation (TBE)^37^. Specifically, the TBE for each clade in the original tree was determined by finding the minimal transfer distance across all clades in the bootstrap tree, followed by computing the mean values across the 100 bootstraps. A TBE cutoff of 0.7 was used, with values above this threshold indicating moderate to strong support.

#### PATH analysis

PATH (phylogenetic analysis of trait heritability) adapts Moran’s I to quantify either the autocorrelation (within cell types) or the cross-correlations (between cell types) of cell phenotypes across the phylogenetic tree^39^. If closely related cells exhibit greater phenotypic resemblance (i.e. from the same cell types) than random cells, the phenotype is considered autocorrelated. To perform the PATH analysis, we created two data matrices: one representing cell-cell phenotypic distances from the phylogenetic tree and the other as a cell-by-cell type matrix with binarized values indicating whether each cell belongs to a specific cell type. Each cell type was then scaled to have a zero mean and unit variance.

#### Generating the “tree of trees”

The phylogenetic tree of 108 gastruloids was constructed based on gastruloid-gastruloid distances derived from monoclonal gastruloid-specific edits of DTTs captured by Debris-seq. To calculate the distance between two gastruloids, the following steps were performed: For each gastruloid, at each of the six sites across all 76 DTTs, the percentage of reads for each specific edit was determined, and combinations with at least 20% of reads were identified as dominant “TapeBC-Site-Edit” combinations. The Jaccard similarity between the two gastruloids’ sets of dominant combinations was calculated, with (1 - similarity) representing their distance. The phylogenetic tree was generated using the UPGMA method in the ape package^70^ in R.

#### Assigning “founders” to pseudo-ancestors

For the DTT data extracted from scRNA-seq, as described above, we split some TapeBCs to account for piggyBac excision and re-integration events, resulting in a matrix of 65,465 cells × 231 DTTs (74 distinct TapeBCs). We then filtered out DTTs observed in fewer than 10 cells and excluded cells with a number of observed DTTs outside the range of mean − 1.5 × SD to mean + 2 × SD. Cell-cell distances were calculated as described previously by retaining DTTs observed in both cells and computing the edit distance for each DTT (containing six edit sites). This distance reflects the number of steps required to transform one DTT into another, including reversing edits and applying forward edits. The average edit distance across all shared DTTs between cell pairs was then calculated, followed by phylogenetic tree generation using the UPGMA method in the ape package^70^ in R.

To assign each of the seven major clades from the “tree of trees” to a pseudo-ancestor in the single-cell phylogenetic tree, we constructed a cell × “TapeBC-Site-Edit” combination matrix (where 1 indicates the combination was detected in the cell and 0 was not) and normalized it by the sum of each column. For each major clade in the “tree of trees”, we identified its exclusively dominant “TapeBC-Site-Edit” combinations. For each pseudo-ancestor in the single-cell phylogenetic tree, we selected the corresponding subset of cells and calculated the fold change between the mean value of dominant combinations for a given major clade and the mean value of dominant combinations across the other six major clades. This step generated a matrix where each row represents a pseudo-ancestor, each column corresponds to a major clade, and the values indicate fold-change. Next, for each major clade, we selected all pseudo-ancestors where the ratio of log2(fold-change + 1) between the given clade and the highest among the remaining six clades was greater than 2, and the one with the highest number of cells was designated as the representative of the major clade.

For each of the seven major clades, we subsetted the cells from its assigned pseudo-ancestor and the gastruloids within that clade, then repeated the assignment. For each gastruloid, we identified its exclusively dominant “TapeBC-Site-Edit” combinations. Then, for each pseudo-ancestor, we calculated the fold change between the mean value of dominant combinations for a given gastruloid and the mean across other gastruloids. This step generated a matrix where each row represents a pseudo-ancestor, each column corresponds to a gastruloid, and the values indicate fold-change. Next, for each gastruloid, we selected all pseudo-ancestors where the ratio of log2(fold-change + 1) between the given clade and the highest among the remaining six clades was greater than 2, and the one with the highest number of cells was designated as the representative of the gastruloids. Through this heuristic, a total of 96 gastruloids were assigned distinct pseudo-ancestors that we believe to correspond to their founding mESC, with these clades collectively comprising 51,963 cells. One gastruloid (P2-B10) was excluded due to the absence of exclusive dominant combinations, and 11 (P1-B10, P2-C9, P1-A5, P1-A4, P2-C3, P2-D12, P1-D9, P1-D8, P1-D6, P2-A12, and P2-D8) were excluded because they could not be assigned to a distinct pseudo-ancestor.

#### Polyclonal and monoclonal gastruloid scRNA-seq data processing and clonotype assignment

- Identification of clonal clusters from Debris-seq: The Debris-seq table contained read counts for 1,014 barcodes × 107 wells. We first removed barcodes and wells with <1,000 total reads each, which left a 948 barcode × 106 well matrix. Six additional wells were flagged as potentially non-monoclonal (Figure S20B) and excluded. After normalizing read counts by per-well totals, we computed pairwise Pearson correlations among the remaining 100 wells and clustered the resulting correlation matrix into 29 clonal clusters of 1-12 gastruloids each (mean 57 barcodes per cluster; range 10-164).
- Transcriptome analysis of gastruloids: Single-cell transcriptomes from 107 monoclonal and 32 polyclonal gastruloids were profiled across 16 lanes on the 10x Genomics platform (monoclonal: 12 lanes; polyclonal: 4 lanes, with 8 individually CMO-labeled gastruloids per lane). Data from each lane were processed separately with Cell Ranger 9.0.1 (default settings), using refdata-gex-GRCm39-2024-A as the reference transcriptome. After filtering low-quality and doublet cells, we retained 56,796 cells from monoclonal gastruloids and 26,929 cells from polyclonal gastruloids. Cells were annotated to 13 cell types based on canonical marker genes.
- Cell-to-clone assignment for monoclonal gastruloids: We generated a cell × barcode matrix from the scRNA-seq data and retained the barcodes shared with the Debris-seq table. Each matrix was normalized by total read counts (per cell or per well, respectively). Multiplying the two matrices (895 barcodes shared between the 100-well Debris-seq matrix and the 55,411-cell scRNA-seq matrix) produced an assignment score for each cell-well pair. Because wells within a clonal cluster share the same barcode pool, well-level assignment is not feasible; instead, we averaged assignment scores across wells within each cluster to perform cell-to-cluster assignment. A cell was assigned to a unique clonal cluster when its top score exceeded the second-highest by ≥3-fold, and clusters with fewer than 200 assigned cells were excluded. In total, 47,418 cells (83% of monoclonal gastruloid cells) were assigned to 26 clonal clusters (mean 1,824 cells per cluster; range 227-8,100).
- Cell-to-clone assignment for polyclonal gastruloids: After CMO demultiplexing, 11,620 polyclonal cells (43% of total) were confidently assigned to individual gastruloids, of which 20 had at least 200 cells (mean 535; range 220-1,291). Applying the same cell-to-cluster assignment procedure to polyclonal gastruloid cells as described above, only 3,899 cells (14% of total) were confidently assigned, of which 82% mapped to a single cluster (cluster 29).

#### Cell capture efficiency across experiments

Using the reference estimates of ~30,000 cells per gastruloid at 144 hours and ~15,000-20,000 cells per gastruloid at 120 hours, we have calculated the proportion of cells captured by scRNA-seq and phylogeny reconstruction for each experiment (Table S7).

## Supplementary Material

1

2 Table S1. Marker genes used for mouse gastruloids cell type annotation (Related to Figures 1, 2, 4, and 5).

3 Table S2. Top 150 genes most highly expressed in spinal cord/r7/r8, NMPs, somites, hindbrain r1-r6, mesodermal progenitors, and MHB, in sci-RNA-seq3 profiled monoclonal gastruloids (Related to Figure 1).

4 Table S3. Top 300 genes most highly expressed in floor plate, node-like cells, notochord, and definitive endoderm, in sci-RNA-seq3 profiled monoclonal gastruloids (Related to Figure 1).

5 Table S4. Top 100 differentially expressed genes in sci-RNA-seq3 profiled monoclonal gastruloids for three comparisons: PSC-derived, ExE endoderm-like cells vs. all other cell types, PSC-derived, ExE endoderm-like cells vs. definitive endoderm, and definitive endoderm vs. PSC-derived, ExE endoderm-like cells (Related to Figure 1).

6 Table S5. Differentially expressed genes in founder mESCs between each of the three categories (PSC, neuroectodermal, and mesodermal) versus mixed (genes with FDR < 0.05 shown) (Related to Figure 5).

7 Table S6. Differentially active TFs in founder mESCs between each of the three categories (PSC, neuroectodermal, and mesodermal) versus mixed (TFs with FDR < 0.05 shown) (Related to Figure 5).

8 Table S7. The proportion of cells captured by scRNA-seq and used for phylogeny reconstruction in each experiment (Related to STAR Methods).

## Figures and Tables

**Figure 1.**
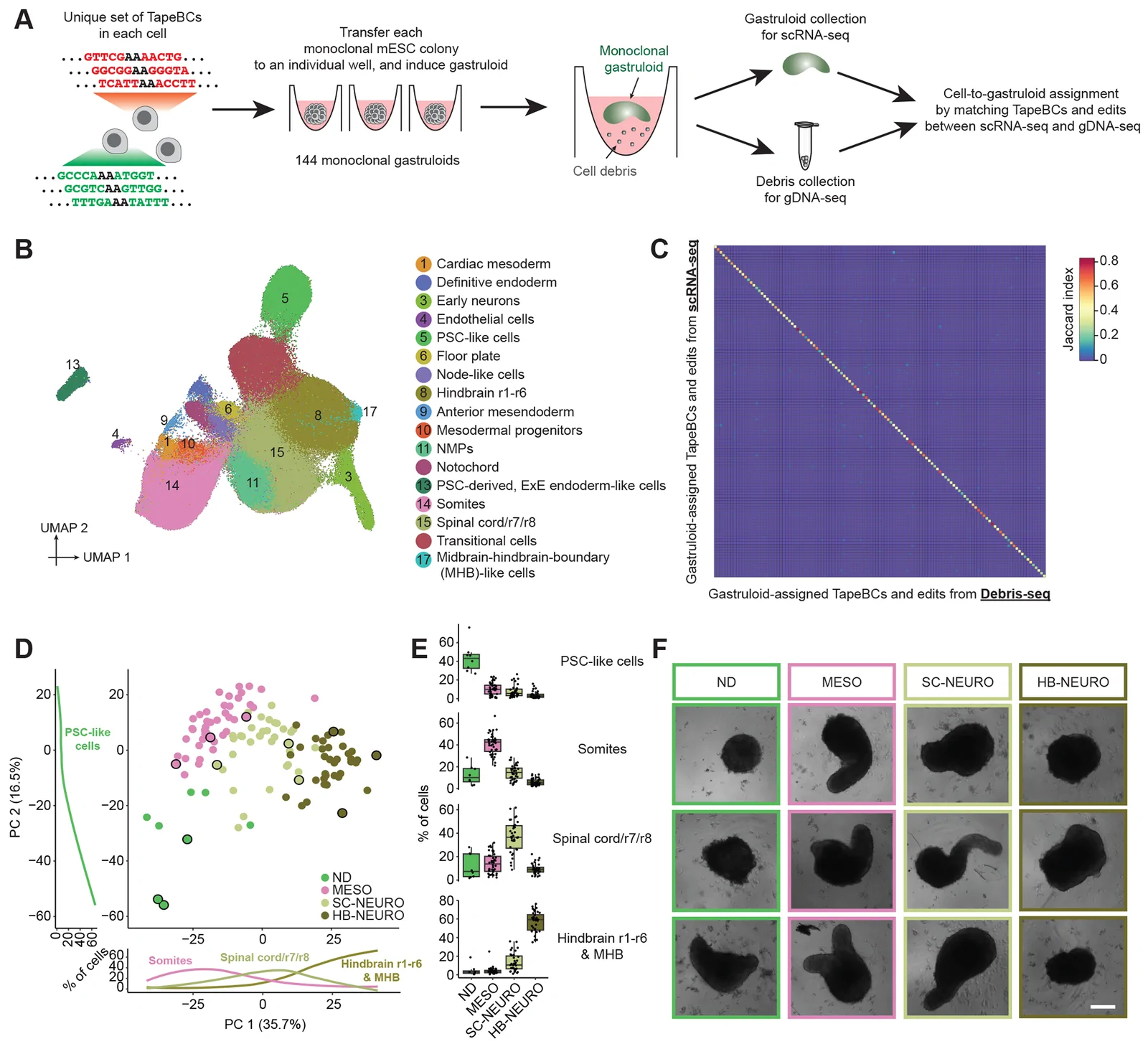
Quantifying phenotypic heterogeneity among mouse gastruloids derived from single cells. **(A)** Cell-to-gastruloid assignment by matching DTTs recovered from scRNA-seq and Debris-seq. **(B)** UMAP of 247,064 cells from 144 gastruloids, colored and numbered to 17 annotations (Table S1). **(C)** Jaccard similarities of DTTs (TapeBCs and edits) assigned to gastruloids by scRNA-seq (rows; ≥5% of assigned cells) or Debris-seq (columns). **(D)** PCA of pseudobulk RNA-seq of 121 gastruloids. Colors indicate 4 groups based on k-means clustering of cell-type compositions: non-differentiating (ND), somite-like (MESO), spinal cord-like (SC-NEURO), or hindbrain-like (HB-NEURO). The percentage of cells belonging to selected cell types is plotted along PC1 (bottom) and PC2 (right). Black circles highlight gastruloids shown in **F**. **(E)** Per-gastruloid proportions of four selected cell types. Boxplots represent IQR (25th, 50th, 75th percentile) with whiskers representing 1.5× IQR. **(F)** Representative images from each group. See also Figure S1–S6; Table S1–S4.

**Figure 2.**
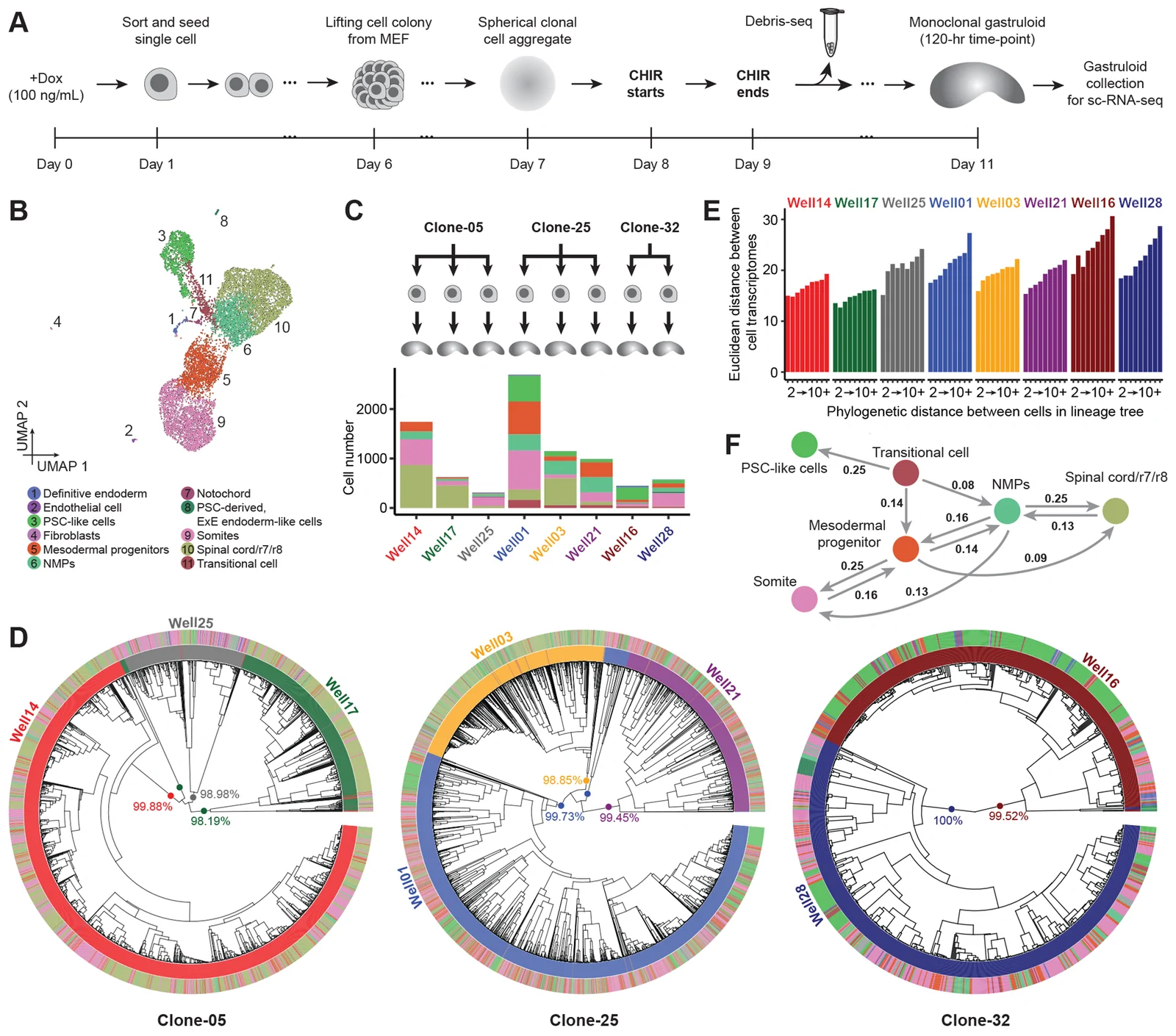
DNA Typewriter-based reconstruction of the cell lineage histories of eight monoclonal gastruloids. **(A)** Schematic of lineage recording experiment. Induction of PEmax was continuous from 24 hours before single-cell seeding through harvest. Eight gastruloids were selected by Debris-seq and profiled on Day 11. **(B)** UMAP of 9,929 cells, colored by cell type. **(C)** Cell-type compositions of 8,545 cells assigned to eight gastruloids. **(D)** Lineage trees of 2,438 (Clone-05), 4,418 (Clone-25), and 960 (Clone-32) gastruloid-assigned cells. Rings from inside out: phylogenetic tree, gastruloid identity (colors in **C**), cell type (colors in **B**). The center of each tree highlights ten major clades, labeled by the % of its cells assigned to a single gastruloid. **(E)** Mean pair-wise transcriptomic distances (Euclidean in 30 PCs) for cells assigned to each gastruloid, grouped by phylogenetic distances (2 to 10+). **(F)** A PATH-inferred^39^ graph of transition probabilities among the six most abundant cell types (>98% of cells, showing transitions with >0.08 probabilities). See also Figure S7–S15; Table S1.

**Figure 3.**
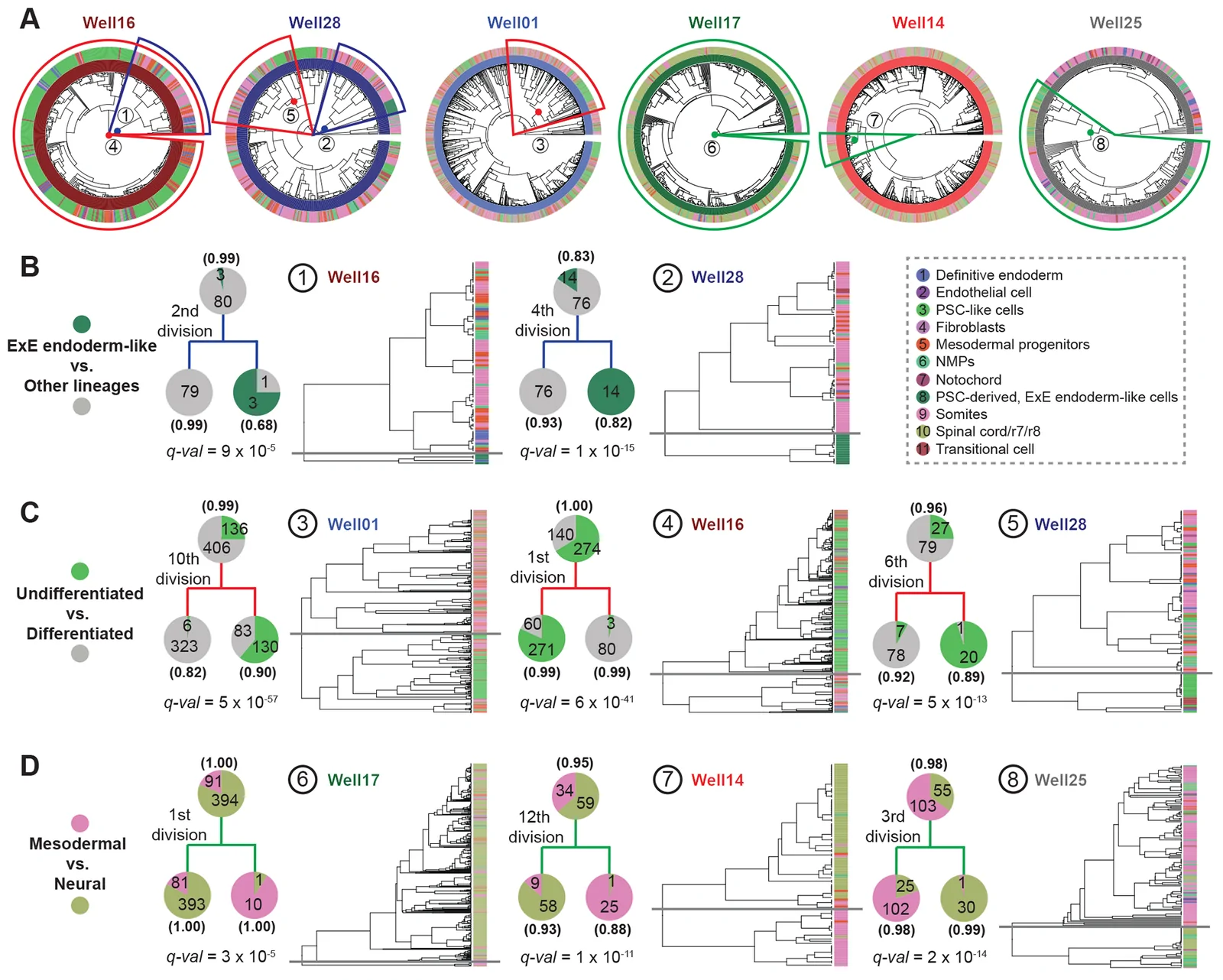
Cell lineage histories suggest fate biases prior to induction. **(A)** Cell lineage trees of individual gastruloids, obtained with DNA Typewriter. Rings as in Figure 2D. Numbered wedges are subclades highlighted in **B-D**. **(B)** Two cell divisions separating ExE endoderm-like cells (dark green) vs. others (gray). **(C)** Three cell divisions separating undifferentiated (PSC-like & transitional; olive green) vs. differentiated (all other; gray). **(D)** Three cell divisions separating mesodermal (somites & mesodermal progenitors; pink) vs. neural (spinal cord & NMPs; light yellow) cell types. In **B-D**: division count relative to a given gastruloid’s root; pie charts include cell numbers by category; TBE^37^ values from 100 bootstraps shown in bold/parentheses; *q*-values obtained by Fisher’s exact test on categorical counts between child nodes, followed by Benjamini-Hochberg (FDR) correction across all ancestor nodes within a given’s gastruloid tree. A subtree view of the clade is shown on the right, with tip cells colored by cell type. The gray horizontal line marks the highlighted cell division. See also Figure S16.

**Figure 4.**
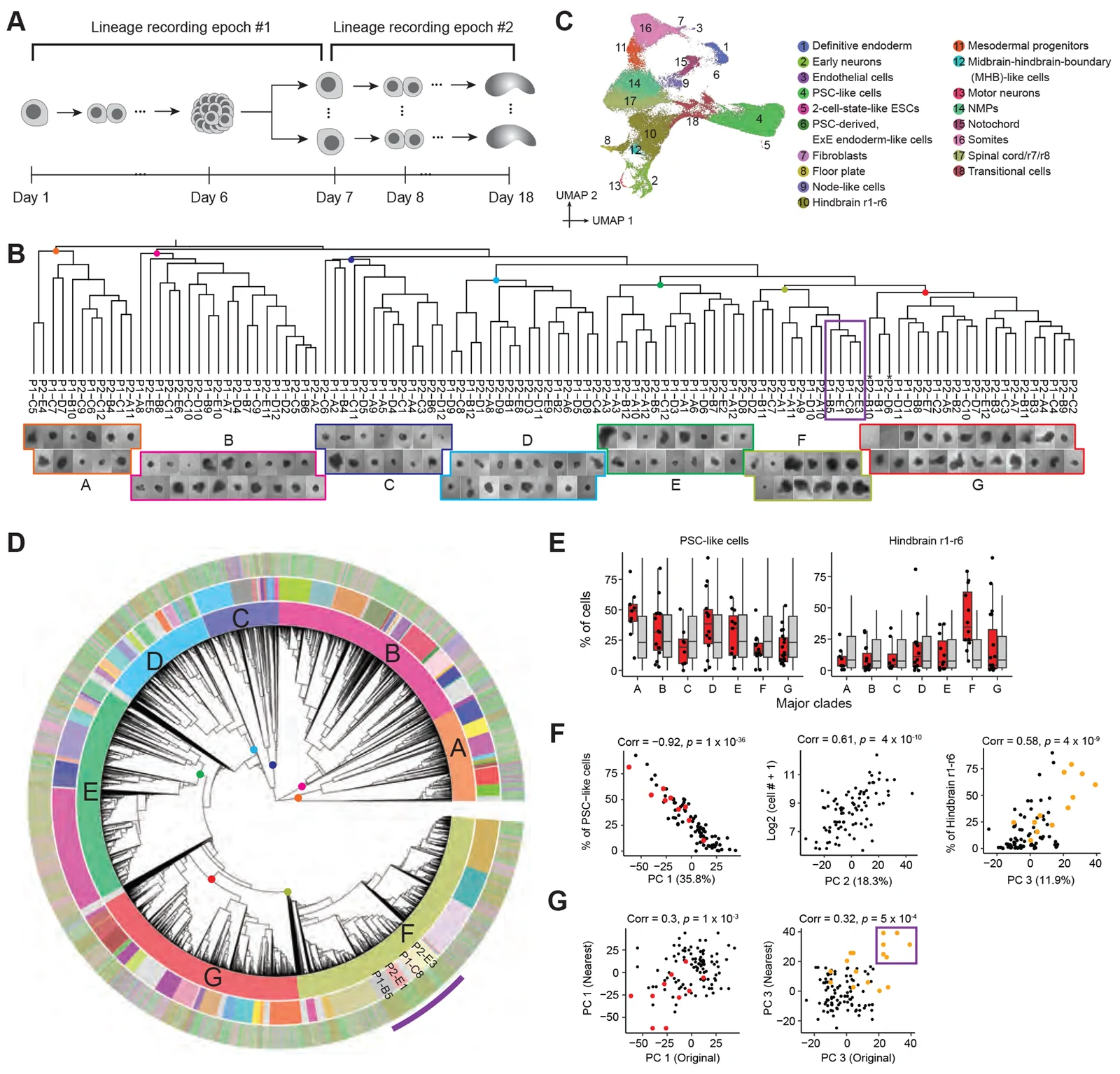
The cell type compositions of monoclonal gastruloids are informed by the relatedness of their founders. **(A)** Two-epoch experiment. A single “founder of founders” mESC was expanded to a colony of 500-1000 cells in a first epoch, which was then isolated, dissociated to single mESCs, and used to induce 108 monoclonal gastruloids in a second epoch. **(B)** A gastruloid tree based on Jaccard distance between dominant edits (STAR Methods), with images at the bottom for individual gastruloids corresponding to seven major clades (A-G). Four clade F gastruloids which have a high percentage of hindbrain cells are highlighted by a purple rectangle (see text and purple arc in **D**). Two gastruloids (P2-D6 & P2-B10, names marked with asterisks, both in clade G) were lost during transfer. **(C)** UMAP of 66,978 cells, colored by cell type. **(D)** Lineage tree of 58,283 cells. Inner ring: seven major clades (colors in **B**). Middle ring: 96 gastruloids. Outer ring: cell type (colors in **C**). Grey represents unassigned cells. The purple arc marks the four hindbrain-rich gastruloids in **B**. **(E)** The proportions of the PSC-like cells (left) and hindbrain cells (right) for 88 individual gastruloids (≥50 cells assigned), grouped by clades. Boxplots, showing 88 replicates from real observations (red) and 5,000 × 88 replicates from permutation (grey) in each subpanel, represent the interquartile range (25th, 50th, 75th percentiles), with whiskers extending to 1.5× the IQR. Black dots represent replicates from the real observations. **(F)** Pseudobulk transcriptional PCA for the same 88 gastruloids: PSC-like proportion versus PC1 (left), log2-transformed cell number versus PC2 (middle), and hindbrain proportion versus PC3 (right). **(G)** PC1 and PC3 values for gastruloids versus their phylogenetic nearest neighbors. The purple rectangle marks six pairs among the four hindbrain-rich gastruloids. In **F** & **G**, clades A and F are colored red and orange, respectively, and Pearson correlations and p-values are shown. See also Figure S17–S19; Table S1.

**Figure 5.**
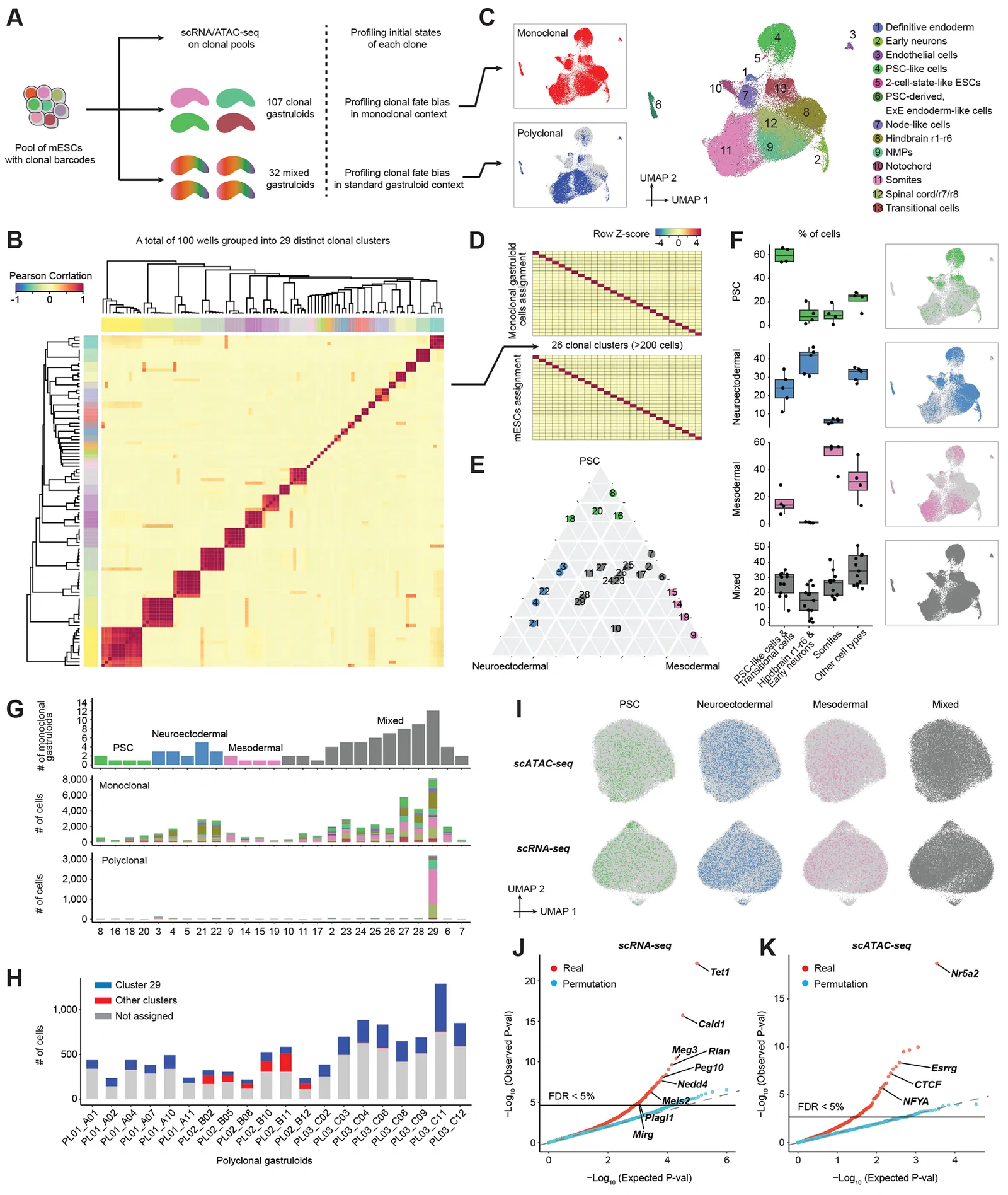
Cryptic differences within nominally homogeneous mESCs are predictive of their gastruloid fate biases. **(A)** Statically barcoded mESCs were bottlenecked to ~50 cells and expanded for multiome profiling and generation of monoclonal gastruloids (scRNA-seq and Debris-seq) and polyclonal gastruloids^12^ (CMO-labeled scRNA-seq; STAR Methods). **(B)** Pairwise Pearson correlations of Debris-seq barcodes from 100 wells, grouped into 29 clusters. **(C)** UMAP of 83,725 cells from 107 monoclonal and 32 polyclonal gastruloids, colored by sample and cell type. **(D)** Average barcode-based assignment scores (STAR Methods) for 26 clusters with >200 cells, for monoclonal gastruloid (top) and for mESCs (bottom). **(E)** Ternary plot of clonal cell-type compositions towards neuroectodermal (hindbrain & early neurons), mesodermal (somites), PSC (PSC-like & transitional cells) or mixed (balanced) fates. **(F)** Cell-type proportions (left) and highlighted UMAP (right) across four groups. Boxplots represent IQR (25th, 50th, 75th percentile) with whiskers representing 1.5× IQR. **(G)** For each of the 26 mESC clonal clusters: the number of monoclonal gastruloids (top), monoclonal gastruloid cell counts by cell type (middle), and polyclonal gastruloid cell counts by cell type (bottom). Cell type colors are the same as in **C**. **(H)** Cells assigned to cluster 29 (blue), other clusters (red), and unassigned (grey) in 20 polyclonal gastruloids with >200 recovered cells. **(I)** UMAPs of 63,889 mESC multiome profiles, colored by fate-bias groups (chromatin accessibility on top and transcriptome on bottom). **(J,K)** Observed vs. expected p-values for differential expression across: 16,605 genes (**J**) and accessibility of 577 TF motifs (**K**). Empirical FDR <5% identified 61 gene-label and 58 motif-label associations (Tables S5–S6; STAR Methods). See also Figure S20; Table S1, S5–S6.

**Table:** Key resources table

REAGENT or RESOURCE	SOURCE	IDENTIFIER
Bacterial and virus strains		
NEB Stable Competent E. coli	New England Biolabs	Cat#C3040H
Chemicals, peptides, and recombinant proteins		
CHIR99021 25mg	Millipore Sigma	Cat#SML1046-25MG
PD0325901	Stemcell Technologies	Cat#72184
ESGRO recombinant mouse LIF protein	Millipore Sigma	Cat#ESG1107
Gelatin Solution	Millipore Sigma	Cat#G1393
NDiff227 medium	Takara	Cat#Y40002
GMEM (Glasgow’s MEM)	Gibco	Cat#11710035
HyClone Characterized FBS, US Origin, 500 mL, Heat-inactivated	Cytiva	Cat#SH30071.03HI
KnockOut Serum Replacement (KSR)	Gibco	Cat#10828028
GlutaMAX Supplement (100X)	Gibco	Cat#35050061
MEM non-essential amino acids (100X)	Gibco	Cat#11140050
Sodium pyruvate (100 mM)	Gibco	Cat#11360070
2-Mercaptoethanol (BME)	Gibco	Cat#21985023
DMEM, high glucose	Thermo Fisher Scientific	Cat#11965118
Collagenase IV	StemCell Technologies	Cat#07909
Doxycycline hyclate	Sigma-Aldrich	Cat#D9891
Puromycin Dihydrochloride	VWR	Cat#A1113803
Hygromycin B (50 mg/mL)	Thermo Fisher Scientific	Cat#10687010
Lipofectamine 2000	Thermo Fisher Scientific	Cat#11668027
Pierce^™^ Premium Grade BS3 (bis[sulfosuccinimidyl]suberate)	Thermo Fisher Scientific	Cat#PG82083
DEPC	Millipore Sigma	Cat#D5758-25ML
Critical commercial assays		
Chromium Next GEM Single Cell 3’ HT Reagent Kit v3.1	10x Genomics	Cat#PN-1000370 (8 rxn); Cat#PN-1000348 (48 rxn)
Chromium Next GEM Single Cell Multiome ATAC + Gene Expression Reagent Bundle	10x Genomics	Cat#PN-1000283 (16 rxn)
Chromium 3’ CellPlex Kit Set A (Cell Multiplexing Oligo)	10x Genomics	Cat#PN-1000261 (48 rxn)
KAPA2G Robust HotStart ReadyMix	Roche	Cat#KK5705
ViraPower Lentiviral Packaging Mix	Thermo Fisher Scientific	Cat#K497500
PEG-it Virus Precipitation Solution (5X)	System Biosciences (SBI)	Cat#LV810A-1
Agencourt AMPure XP - 450 ml	Beckman Coulter	Cat#A63882
HiScribe T7 High Yield RNA Synthesis Kit	New England Biolabs	Cat#E2050S
ARCTURUS PicoPure DNA Extraction Kit	Thermo Fisher Scientific	Cat#KIT0103
Deposited data		
Raw and processed single-cell sequencing data	This paper; NCBI GEO	GEO: GSE291244; https://www.ncbi.nlm.nih.gov/geo/query/acc.cgi?acc=GSE291244
Gastruloid scRNA-seq data from Suppinger et al.	NCBI GEO	GEO: GSE229513; https://www.ncbi.nlm.nih.gov/geo/query/acc.cgi?acc=GSE229513 Publication DOI: https://doi.org/10.1016/j.stem.2023.04.018
Trunk-like structure scRNA-seq data from Veenvliet et al.	NCBI GEO	GEO: GSE141175; https://www.ncbi.nlm.nih.gov/geo/query/acc.cgi?acc=GSE141175 Publication DOI: https://doi.org/10.1126/science.aba4937
Mouse prenatal data from Qiu et al.	NCBI GEO	GEO: GSE186069; GEO: GSE228590; https://www.ncbi.nlm.nih.gov/geo/query/acc.cgi?acc=GSE186069 https://www.ncbi.nlm.nih.gov/geo/query/acc.cgi?acc=GSE228590 Publication DOI: https://doi.org/10.1038/s41586-024-07069-w
Mouse gastrulation data from Pijuan-Sala et al.	ArrayExpress / BioStudies	ArrayExpress: E-MTAB-6967; https://www.ebi.ac.uk/biostudies/arrayexpress/studies/E-MTAB-6967 Publication DOI: https://doi.org/10.1038/s41586-019-0933-9
Gastruloid scRNA-seq data from Rosen et al.	NCBI GEO	GEO: GSE212050; https://www.ncbi.nlm.nih.gov/geo/query/acc.cgi?acc=GSE212050 Publication DOI: https://doi.org/10.1101/2022.09.27.509783
Gastruloid scRNA-seq data from van den Brink et al.	NCBI GEO	GEO: GSE123187; https://www.ncbi.nlm.nih.gov/geo/query/acc.cgi?acc=GSE123187 Publication DOI: https://doi.org/10.1038/s41586-020-2024-3
Custom mouse reference genome (GRCm39 + DTT sequence)	This paper	N/A
Experimental models: Cell lines		
Mouse: E14TG2a embryonic stem cells	Gift from C. Schroter	RRID: CVCL_9108
Mouse: CF-1 mouse embryonic fibroblasts, P3, mitomycin-C treated	Applied StemCell	Cat#ASF-1223
Mouse: E14TG2a polyclonal DNA Typewriter lineage-tracing mESC line	This paper	N/A
Mouse: DNA Typewriter lineage-recording clones (Clone-05, Clone-25, Clone-32)	This paper	N/A
Oligonucleotides		
Genomic DNA DTT enrichment forward primer: GCGTCAGATGTGTATAAGAGACAGGGTACCATAGCAGATGATCCATGGTC	This paper	N/A
Genomic DNA DTT enrichment reverse primer: TTCAGACGTGTGCTCTTCCGATCTttttgtaatccagaggttgattgtcgacttac	This paper	N/A
cDNA DTT enrichment reverse primer: TTCAGACGTGTGCTCTTCCGATCTGGTACCATAGCAGATGATCCATGGTC	This paper	N/A
Recombinant DNA		
Lenti-TAPE (lentiviral vector including 8N static barcodes)	This paper	N/A
PB-TAPE (U6-epegRNA and GFP-DNA Tape cassettes)	This paper	N/A
PB-iPEmax-HygroR (Dox-inducible PEmax-P2A-mCherry; rtTA3-P2A-HygroR)	This paper	N/A
PB-PuroR (piggyBac puromycin-resistance)	This paper	N/A
Super PiggyBac Transposase Expression Vector	System Biosciences (SBI)	Cat#PB210PA-1
Software and algorithms		
sci-RNA-seq3 processing pipeline	Cao et al.^59^	https://github.com/JunyueC/sci-RNA-seq3_pipeline
bcl2fastq v2.20	Illumina	https://support.illumina.com/
deML	Renaud et al.^60^	https://github.com/grenaud/deML
Trim Galore v0.6.5	Krueger	https://github.com/FelixKrueger/TrimGalore
STAR v2.6.1d	Dobin et al. ^61^	https://github.com/alexdobin/STAR
HTSeq	Anders et al.^62^	https://htseq.readthedocs.io/en/latest/
Scrublet v0.1	Wolock et al.^63^	https://github.com/swolock/scrublet
Scanpy v1.6.0	Wolf et al.^64^	https://scanpy.scverse.org/en/stable/
Seurat v3 and v5	Stuart et al.^65^; Hao et al.^68^	https://satijalab.org/seurat/
Cell Ranger 7.2.0 and 9.0.1	10x Genomics	https://www.10xgenomics.com/support/software/cell-ranger/lates t
cellranger-arc 2.0.2	10x Genomics	https://www.10xgenomics.com/support/software/cell-ranger/latest
ape 5.0	Paradis & Schliep^70^	https://emmanuelparadis.github.io/
phytools (phylosig, Pagel’s lambda)	Revell	https://cran.r-project.org/web/packages/phytools/index.html
PATH (phylogenetic analysis of trait heritability)	Schiffman et al.^39^	https://github.com/landau-lab/PATH
SCTransform	Hafemeister & Satija^69^	https://satijalab.org/seurat/articles/sctransform_vignette
DBSCAN (scikit-learn)	Pedregosa et al.	https://scikit-learn.org/stable/
JASPAR position weight matrices; MOODS scanning	JASPAR	https://jaspar.elixir.no/
Custom analysis code for this study	This paper	https://github.com/shendurelab/DTT
DNA Typewriter MOI inference code	This paper	https://github.com/SanjayKottapalli/DNA-Typewriter-MOI-Inference/
Other		
Illumina NextSeq 2000 sequencer	Illumina	N/A

## Data Availability

The data generated in this study can be downloaded in raw and processed forms from the NCBI Gene Expression Omnibus under accession number GSE291244. The code used for all data processing and analysis is available at https://github.com/shendurelab/DTT and archived at Zenodo (https://doi.org/10.5281/zenodo.22756081) except for the code specific to inferring DNA Typewriter MOI, which is available at https://github.com/SanjayKottapalli/DNA-Typewriter-MOI-Inference/ and archived at Zenodo (https://doi.org/10.5281/zenodo.22818360).

## HIGHLIGHTS

1. Gastruloids grown from single mESCs vary widely in cell type composition
2. DNA Typewriter resolves lineage trees from one mESC to ~50,000 descendant cells
3. Fate biases are heritable and arise well before gastruloid induction
4. Cryptic *Tet1* and imprinted locus states in mESCs predict gastruloid composition
