# Supplementary material for: A fluctuation test for gastruloid heterogeneity
Source: Cell. Author manuscript; Available in PMC 2026 Nov 9. (PMC13746981; doi:10.1016/j.cell.2026.09.057)
Supplement: Supplementary file 1 [file NIHMS2216708-supplement-1.pdf]

Supplemental figures

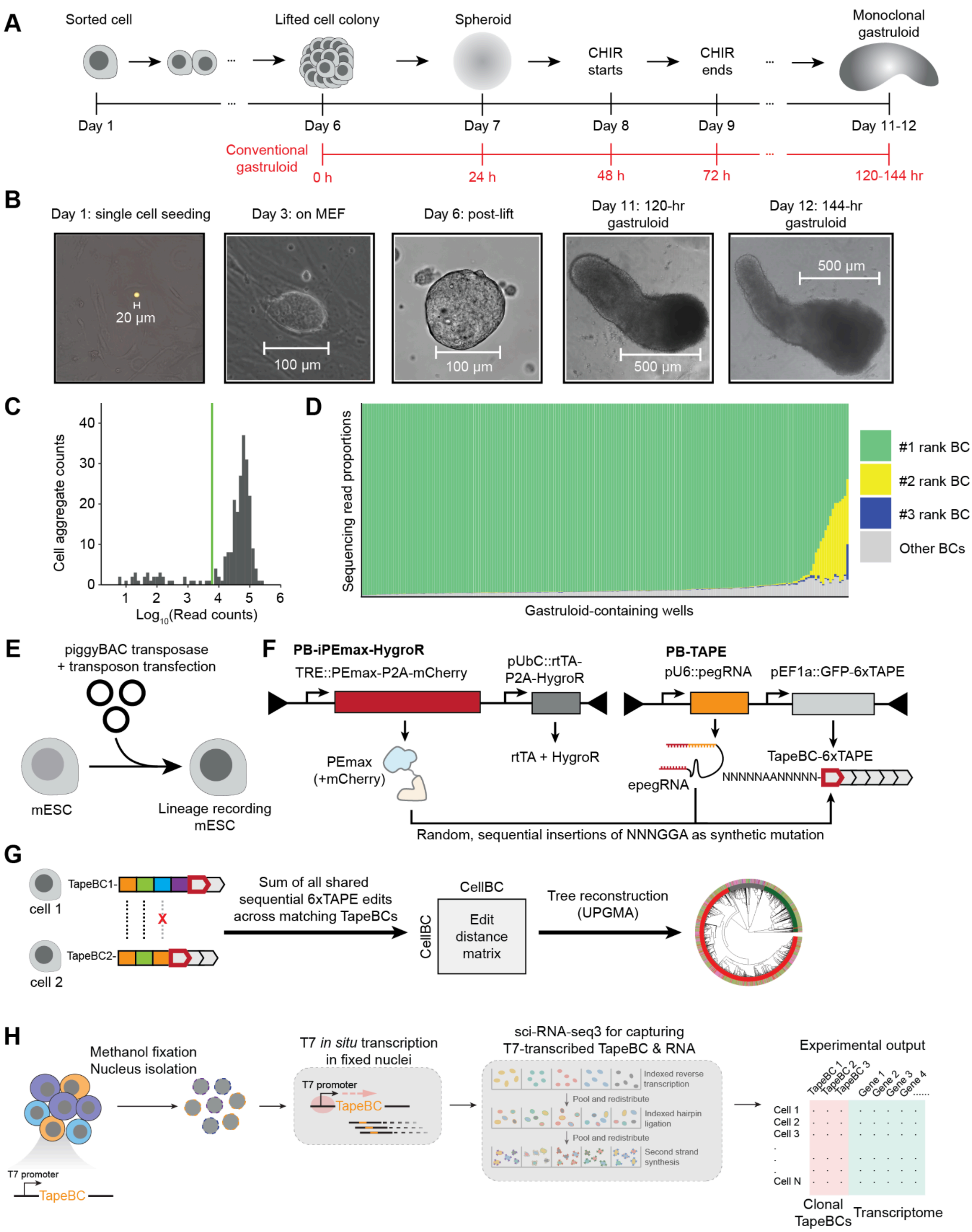

Figure S1. A protocol for generating monoclonal gastruloids (Related to Figure 1).

(A) Schematic of protocol for generating monoclonal mouse gastruloids. Dissociated mESCs are FACS-sorted followed by density controlled seeding onto a layer of MEF feeder cells. After single cells expand into monoclonal colonies, they are lifted off intact through collagenase IV treatment and gentle agitation, and transferred to a nonadherent plate where they give rise to spherical aggregates. The aggregates are then transferred again to an individual well where they undergo induction via 24-hr exposure to CHIR, a Wnt agonist. Timeline from the conventional mouse gastruloid protocol[1] is displayed below in red text for a comparison. **(B)** Bright-field images of representative gastruloids from days 1, 3, 6, 11, and 12 are presented. For the day 1 image, GFP (green) and mCherry (red) fluorescence, also shown, were used to locate a GFP+/mCherry+ single cell on the MEF feeder cell layer. **(C)** Sequencing of static 8N DNA barcodes. Histogram of log-scaled read counts assigned to each of 234 wells. 201/234 wells were assigned ≥6,000 reads (green vertical line). **(D)** For each of the 201 wells with ≥6,000 assigned reads (x-axis), stacked bar plots of the proportions of reads corresponding to the top three most abundant DNA barcodes observed in that well (y-axis). In 93% of these wells, the top-ranked barcode accounted for ≥90% of the observed barcodes. **(E)** Schematic of transfecting mESCs with three different kinds of DNA plasmids: transposon bearing DNA Typewriter Tape (PB-TAPE), transposon bearing Dox-inducible Prime Editor expression cassette (PB-iPEmax-HygroR), and plasmid expressing piggyBac transposase. **(F)** Architecture of two transposons: PB-TAPE contains a human U6 promoter driving epegRNA expression (pU6::epegRNA, where epegRNA inserts one of 64 possible NNNGGA sequences to DNA Typewriter Tape targets) and human EF1a promoter driving GFP-6xTAPE expression (pEF1a::GFP-6xTAPE), where 12-bp (NNNNNAANNNNN) TapeBC and 6xTAPE (DNA Typewriter Tape with 6 sequential editing sites) embedded in 3'-UTR of eGFP-encoding transcript. PB-iPEmax-HygroR contains a Dox-inducible promoter driving expression of PEmax-P2A-mCherry (TRE::PEmax-P2A-mCherry) and a human ubiquitin C promoter driving rtTA-P2A-HygroR expression. Dox-induction of modified cells result in expression of PEmax, which complexes with the epegRNAs to program the sequential editing of 6xTAPE with NNNGGA insertions. This results in a heritable DNA barcode used for lineage tracing and lineage tree reconstruction. **(G)** Schematic of algorithmic procedures for DTT-based cell lineage tree reconstruction. For each cell pair, all the recovered TapeBC-6xTAPE insertions are compared to generate a cell-by-cell edit distance matrix, where each entry is the count of matching sequential edits on the 6xTAPE sharing the same TapeBC. A lineage tree is reconstructed based on this matrix using the Unweighted Pair Group Method with Arithmetic Mean (UPGMA) method. **(H)** Schematic of experimental procedures for T7-assisted sci-RNA-seq3[2]. In brief, nuclei from cells containing DTTs are fixed with methanol, isolated, and subjected to T7 polymerase-mediated *in situ* transcription. T7-transcribed DTTs are then captured through the sci-RNA-seq3 protocol along with other RNAs in each nucleus. The sci-RNA-seq3 method is an exponentially scalable single-cell RNA-seq profiling method based on three levels of split-pool combinatorial indexing[3].

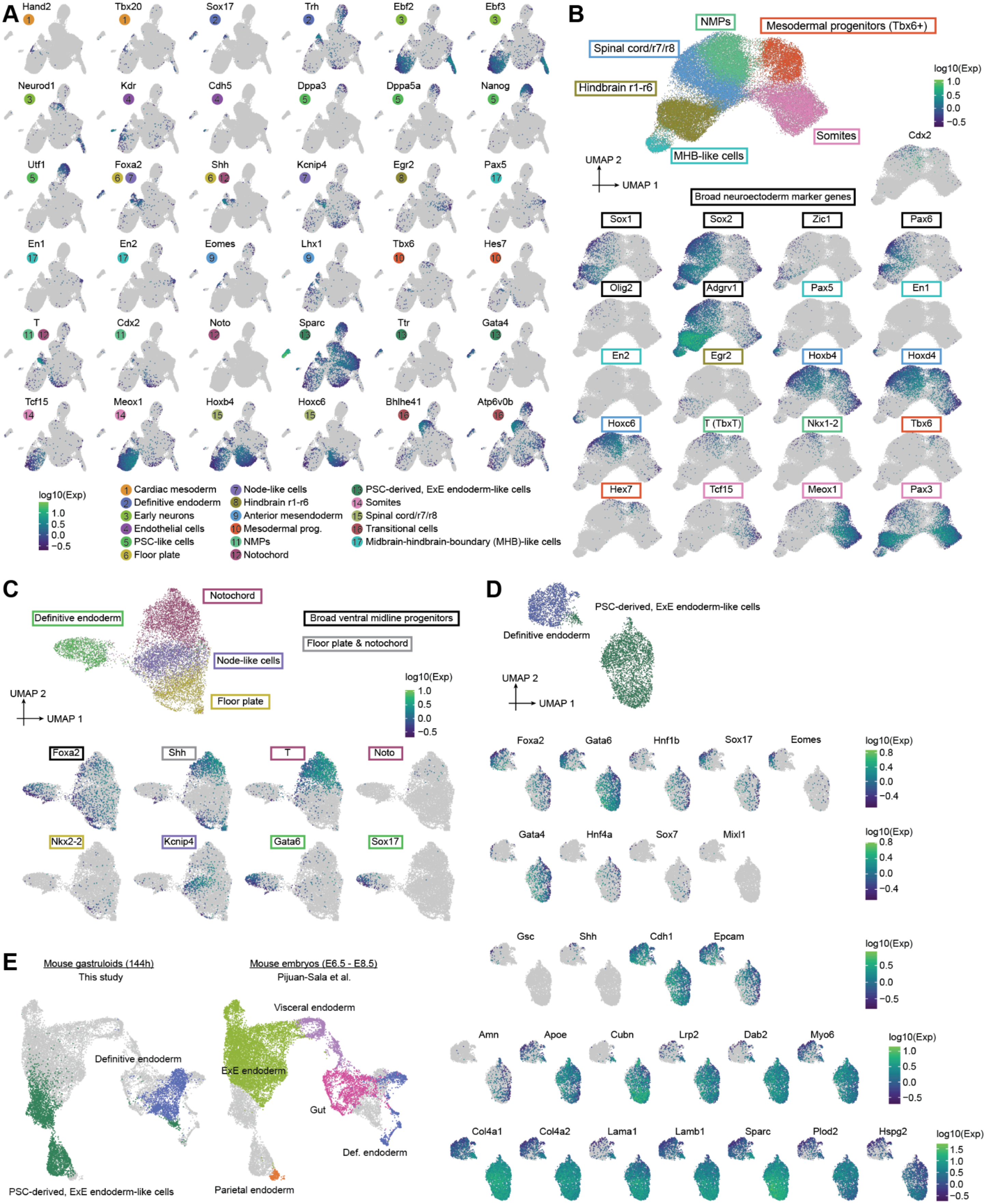
A
Hand2
Tbx20
Sox17
Trh
Ebf2
Ebf3
Neurod1
Kdr
Cdh5
Dppa3
Dppa5a
Nanog
Utf1
Foxa2
Shh
Kcnip4
Egr2
Pax5
En1
En2
Eomes
Lhx1
Tbx6
Hes7
T
Cdx2
Noto
Sparc
Ttr
Gata4
Tcf15
Meox1
Hoxb4
Hoxc6
Bhlhe41
Atp6v0b
log10(Exp)
1.0
0.5
0.0
−0.5
1 Cardiac mesoderm
2 Definitive endoderm
3 Early neurons
4 Endothelial cells
5 PSC-like cells
6 Floor plate
7 Node-like cells
8 Hindbrain r1-r6
9 Anterior mesendoderm
10 Mesodermal prog.
11 NMPs
12 Notochord
13 PSC-derived, ExE endoderm-like cells
14 Somites
15 Spinal cord/r7/r8
16 Transitional cells
17 Midbrain-hindbrain-boundary (MHB)-like cells
B
NMPs
Mesodermal progenitors (Tbx6+)
Spinal cord/r7/r8
Hindbrain r1-r6
Somites
MHB-like cells
UMAP 2
UMAP 1
log10(Exp)
1.0
0.5
0.0
−0.5
Cdx2
Broad neuroectoderm marker genes
Sox1
Sox2
Zic1
Pax6
Olig2
Adgrv1
Pax5
En1
En2
Egr2
Hoxb4
Hoxd4
Hoxc6
T (TbxT)
Nkx1-2
Tbx6
Hex7
Tcf15
Meox1
Pax3
C
Notochord
Definitive endoderm
Broad ventral midline progenitors
Floor plate & notochord
Node-like cells
Floor plate
UMAP 2
UMAP 1
log10(Exp)
1.0
0.5
0.0
−0.5
Foxa2
Shh
T
Noto
Nkx2-2
Kcnip4
Gata6
Sox17
D
PSC-derived, ExE endoderm-like cells
Definitive endoderm
UMAP 2
UMAP 1
Foxa2
Gata6
Hnf1b
Sox17
Eomes
log10(Exp)
0.8
0.4
0.0
−0.4
Gata4
Hnf4a
Sox7
Mixl1
log10(Exp)
0.8
0.4
0.0
−0.4
Gsc
Shh
Cdh1
Epcam
log10(Exp)
0.5
0.0
−0.5
Amn
Apoe
Cubn
Lrp2
Dab2
Myo6
log10(Exp)
1.0
0.5
0.0
−0.5
Col4a1
Col4a2
Lama1
Lamb1
Sparc
Plod2
Hspg2
log10(Exp)
1.5
1.0
0.5
0.0
−0.5
E
Mouse gastruloids (144h)
This study
Mouse embryos (E6.5 - E8.5)
Pijuan-Sala et al.
Definitive endoderm
PSC-derived, ExE endoderm-like cells
Visceral endoderm
ExE endoderm
Gut
Def. endoderm
Parietal endoderm

Figure S2. Transcriptional heterogeneity in mouse gastruloids (Related to Figure 1).

(A) The same UMAP as in **Figure 1B**, colored by gene expression of marker genes for each cell cluster. References for marker genes are provided in **Table S1**. **(B)** 2D UMAP visualization of 46,705 cells from selected cell types in monoclonal gastruloids profiled by sci-RNA-seq3, with each cell type randomly downsampled to roughly equal numbers. The same UMAP is shown multiple times below, colored by the expression of marker genes for each cell cluster. **(C)** 2D UMAP visualization of 11,566 cells from selected cell types in monoclonal gastruloids profiled by sci-RNA-seq3. The same UMAP is shown multiple times below, colored by the expression of marker genes for each cell cluster. **(D)** 2D UMAP visualization of 5,787 cells from selected cell types in monoclonal gastruloids profiled by sci-RNA-seq3. The same UMAP is shown multiple times below, each colored by the expression of genes relevant to our annotation of the two clusters as definitive endoderm and PSC-derived ExE endoderm-like cells, including: (i) endoderm-related transcription factors (*Foxa2, Gata6, Hnf1b, Sox17, Eomes, Gata4, Hnf4a, Sox7, Mixl1, Gsc*) or other genes (*Shh, Cdh1, Epcam*) (ii) nutrient uptake genes characteristic of yolk sac endoderm (*Amn, Apoe, Cubn, Lrp2, Dab2, Myo6*), and (iii) genes associated with basement membrane and extracellular matrix (*Col4a1, Col4a2, Lama1, Lamb1, Sparc, Plod2, Hspg2*). **(E)** UMAP visualization of 18,817 co-embedded endoderm-lineage cells from two datasets, including the monoclonal gastruloids profiled in this study (left) and mouse embryos during gastrulation (E6.5–E8.5)[4] (right), after batch correction[5].

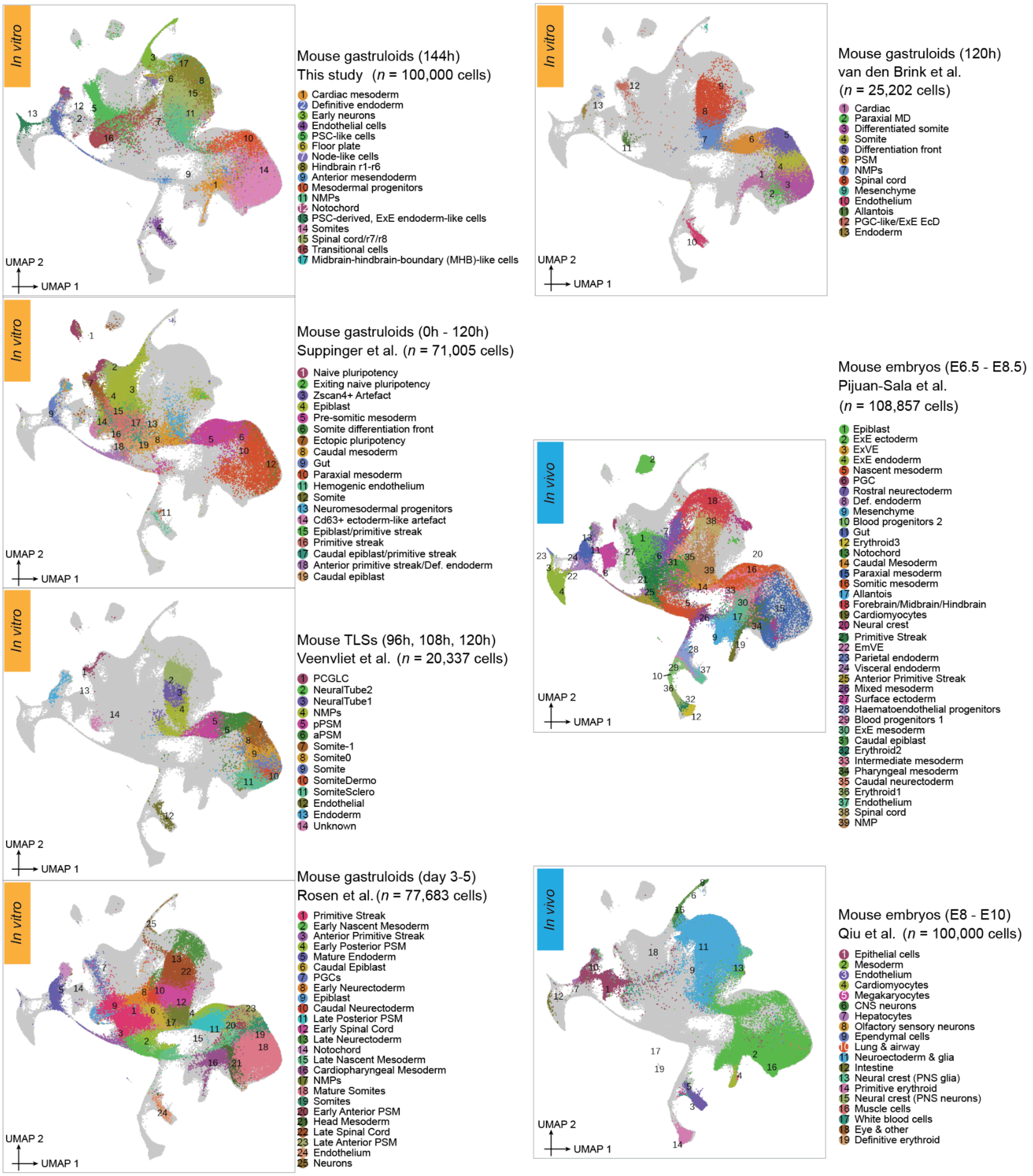

Figure S3. Integrating & co-embedding cells from seven studies of mouse gastruloids or embryos (Related to Figure 1).

UMAP visualization of 503,084 co-embedded cells from seven datasets, including mouse gastruloids from this study, four published mouse gastruloid studies[6–9], and two studies of mouse embryos during gastrulation (E6.5-E8.5)[4] or early somitogenesis (E8-E10)[10], after batch correction[5]. The same UMAP is shown seven times, with colors highlighting cells from each dataset. To align with the cell counts in the other datasets, sci-RNA-seq3 profiles from this study (top left) and mouse embryos early somitogenesis[10] (bottom right) were randomly subsampled to 100,000 cells each.

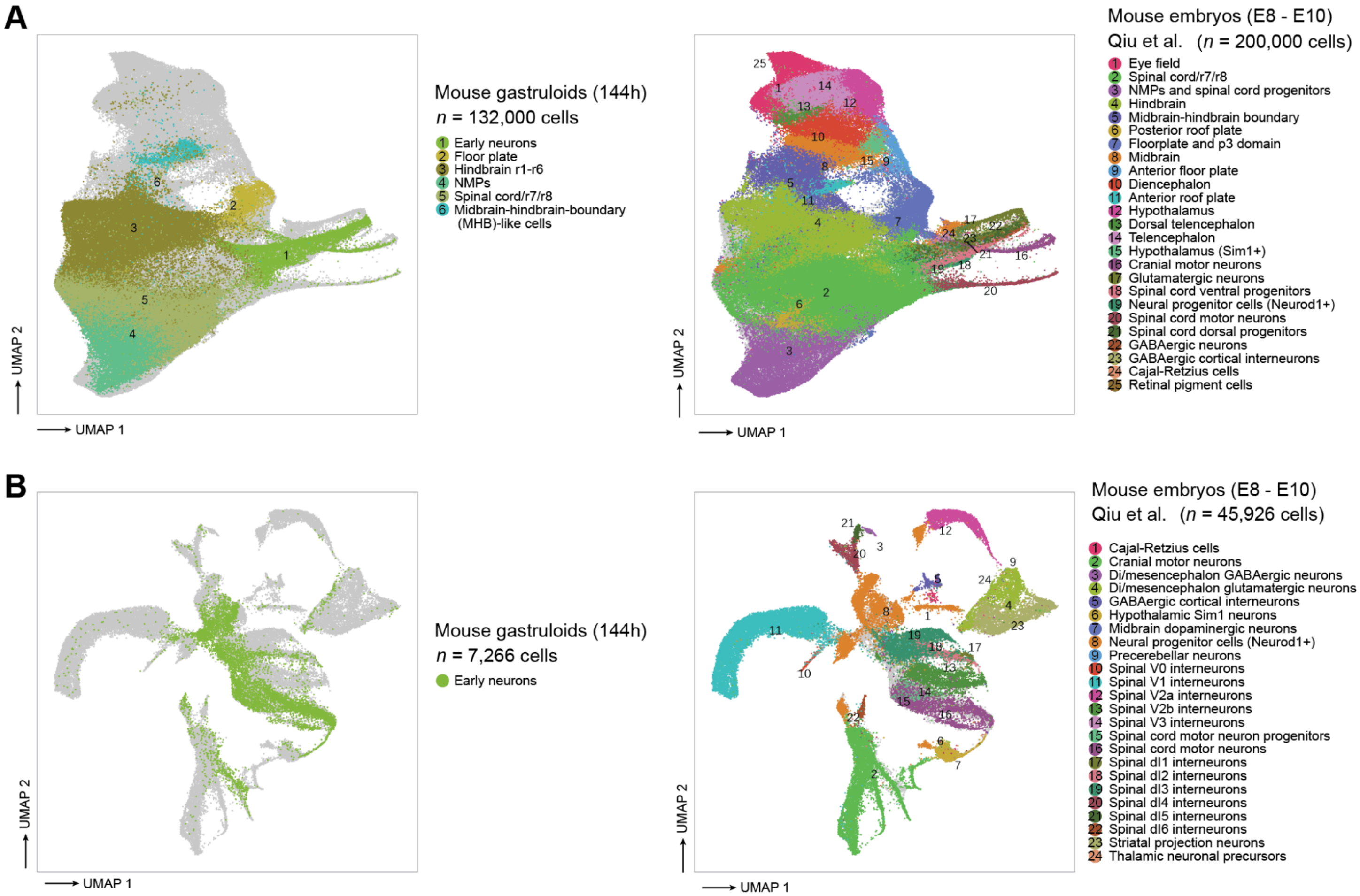

Figure S4. Integrating & co-embedding patterned neuroectodermal and neuronal cells from mouse gastruloids and embryos (Related to Figure 1).

(A) UMAP visualization of single cell transcriptional profiles of 332,000 nuclei annotated as patterned neuroectodermal cell types from either 144 hr mouse gastruloids (left; this study) or mouse embryos during early somitogenesis (E8-E10)[10] (right), after batch correction[5]. The same UMAP is shown twice, with colors highlighting cells from each dataset. Cells from the mouse embryo study were randomly subsampled to 200,000. **(B)** Same as **A**, but for 53,192 nuclei annotated as neuronal cell types in the same two studies.

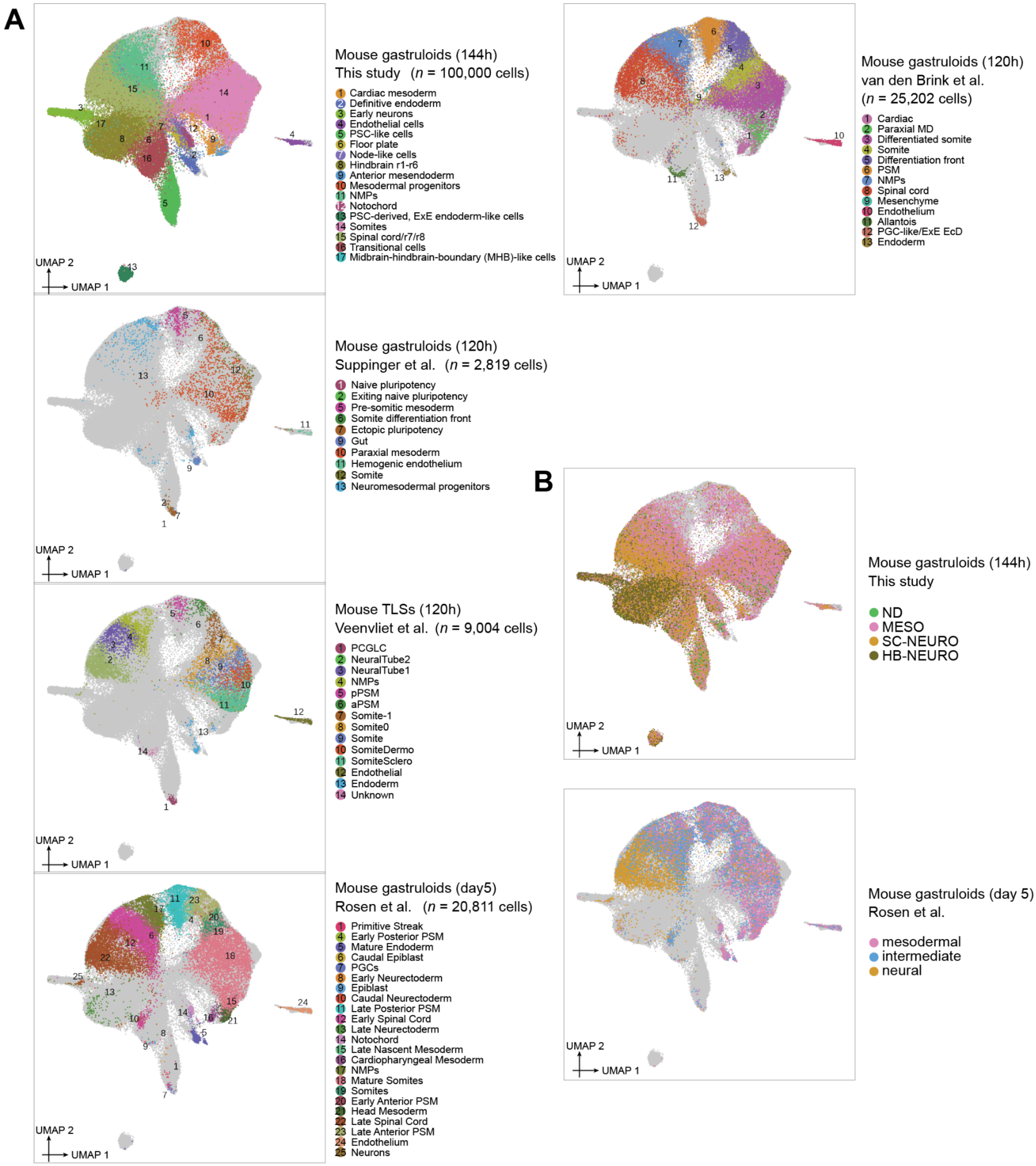

Figure S5. Integrating & co-embedding cells from mouse gastruloids from this study (144 hrs) and four other studies (120 hr time point only; Related to Figure 1).

(A) UMAP visualization of 157,836 co-embedded cells from five datasets, including mouse gastruloids from this study (144 hours), and four other published mouse gastruloid studies (120 hours)[6–9], after batch correction[5]. The same UMAP is shown five times, with colors highlighting cells from each dataset. To align with the cell counts in the other datasets, cells from the current study (top left) were randomly subsampled to 100,000 cells each. **(B)** The same UMAP as shown in **A** is displayed twice, with colors highlighting cells from the four gastruloid groups delineated in this study (top), or from the clusters delineated by Rosen et al. (2022)[6].

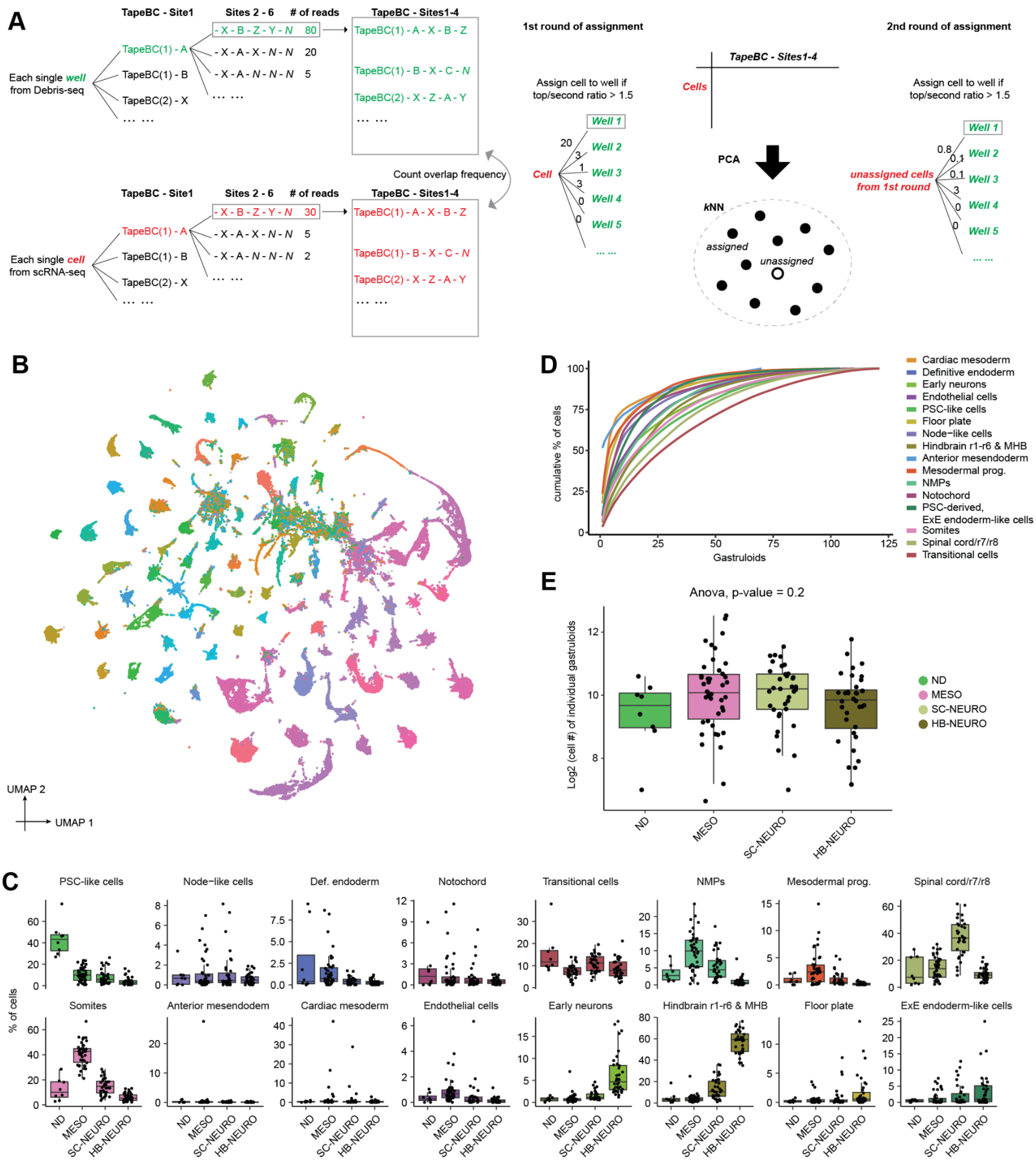

Figure S6. Assigning cells & assessing heterogeneity across 121 monoclonal mouse gastruloids (Related to Figure 1).

(A) Schematic of the cell-to-gastruloid assignment pipeline (**Figure 1**). DTTs recovered by scRNA-seq are matched to a Debris-seq-derived whitelist of well-specific DTTs. A cell is assigned to a well when its top cell–well DTT overlap exceeds the second-best by >50%, either directly or, for initially unassigned cells, via nearest neighbors in DTT-based PCA space. **(B)** 2D UMAP visualization of 154,988 cells (with ≥3 TapeBCs detected) derived from 121 monoclonal mouse gastruloids. Dimensionality reduction was performed on a TapeBC × cell matrix with read counts as values, using PCA followed by

UMAP purely for visualization purposes. Cells are colored according to the gastruloids to which they were assigned. Unassigned cells (4%) are depicted in gray. **(C)** The proportions of sixteen cell types (with hindbrain r1-r6 and MHB combined) for individual gastruloids were calculated, and their distributions within each of the four groups (ND, MESO, SC-NEURO, HB-NEURO) are presented. Boxplots represent IQR (25th, 50th, 75th percentile) with whiskers representing 1.5× IQR. **(D)** For each cell type, the cumulative percentage of cells was plotted across 121 gastruloids, ranked by the abundance of that cell type from highest to lowest. **(E)** Log2 number of cells assigned to individual gastruloids (n = 121) for each of the four groups. Boxplots represent IQR (25th, 50th, 75th percentile) with whiskers representing 1.5× IQR. ND: non-differentiating, MESO: somite-like, SC-NEURO: spinal cord-like, or HB-NEURO: hindbrain-like.

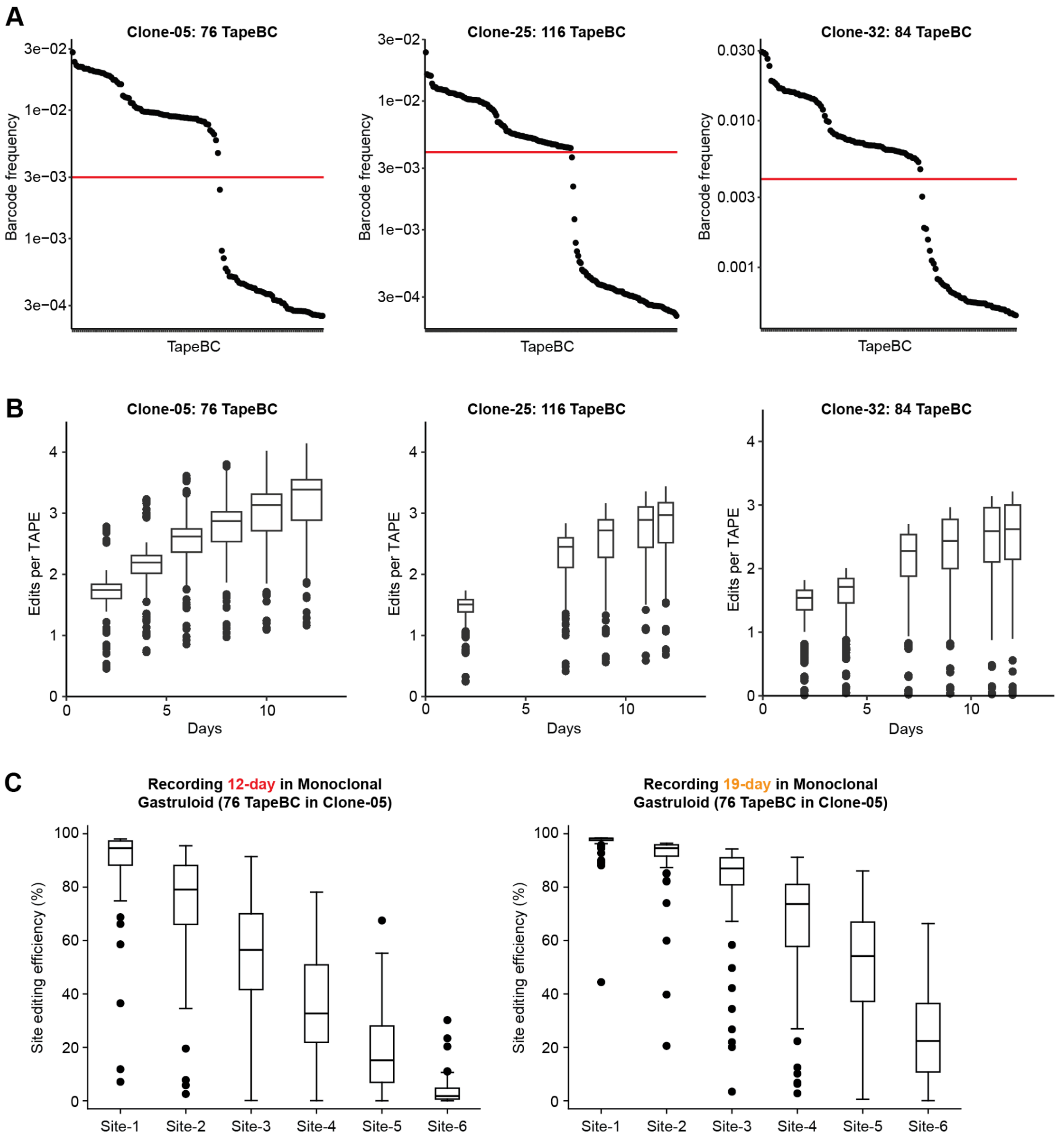

Figure S7. Characterization of three monoclonal mESC cell lines for recording capacity for DNA Typewriter-based lineage recording (Related to Figure 2).

(A) TapeBC barcodes from each of three monoclonal mESC cell lines were PCR amplified and sequenced from genomic DNA. Frequency thresholds (red line) to define bona fide TapeBC barcodes were set as shown, resulting in estimates of 76, 116, and 84 DTT/epegRNA integrations for Clone-05, Clone-25, and Clone-32, respectively. Note that these are lower bounds, as some integrants are duplicated by piggyBac excision and re-integration events[2,11]**. (B)** The accumulation of editing at DTT in each monoclonal mESC cell line with continuous doxycycline (Dox) treatment to induce PEmax

expression. 100 ng/mL Dox was added to cells at Day 0, and cell lines were collected at multiple time points of 2-3 days in interval, depending on the availability of cells at the time of the passaging and collection. The experiment was terminated at Day 12, which is equivalent to the last time point in the monoclonal gastruloid induction protocol. **(C)** Editing efficiency across the six sequential editing sites of DTT (Site-1 to Site-6) during the "eight monoclonal gastruloid" experiment (12-day recording; **Figure 2**; left) and the "tree-of-trees" experiment (19-day recording; **Figure 4**; right). Individual dots represent the site editing efficiency of each DTT (76 TapeBC in Clone-05). Boxplot represents IQR (25th, 50th, 75th percentile) with whiskers representing 1.5× IQR.

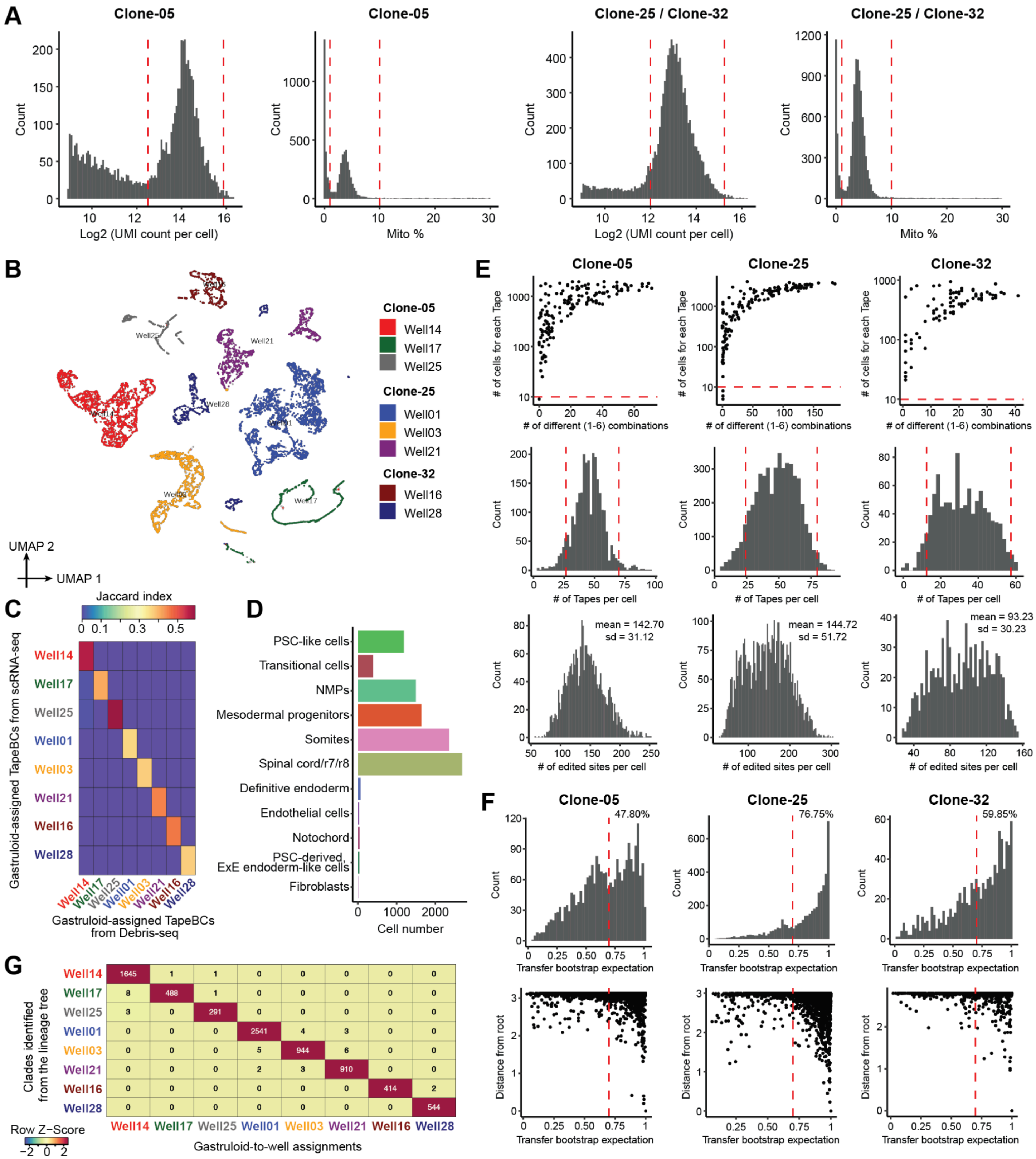

Figure S8. Quality control for scRNA-seq and cell-to-gastruloid assignments (Related to Figure 2).
(A) Histograms of log2(UMI count) per cell and the proportion of reads mapping to mitochondrial chromosomes (Mito%) per cell, derived from scRNA-seq data (10X Genomics) from Lane 1 (left, Clone-05) and Lane 2 (right, Clones-25 & 32). Cells with log2(UMI counts) <12.5 in Lane 1 or <12 in Lane 2 were excluded, as were those in the top 0.5% of total UMI counts (red vertical lines) and those with Mito% >10% or <1% (red vertical lines). **(B)** 2D UMAP visualization of 8,547 cells, with ≥2 TapeBCs detected and TapeBC UMIs ≥10. Dimensionality reduction was performed with PCA on a TapeBC × cell matrix with read counts as values, followed by UMAP solely for visualization. Cells are colored by their assignment

to one of eight monoclonal gastruloids. Unassigned cells (2%) are depicted in light gray. **(C)** Jaccard similarities between the TapeBCs detected in cells assigned to each gastruloid (row; detected in ≥5% of the cells within that gastruloid) vs. TapeBCs detected by Debris-seq of each well (column). Only TapeBCs detected in both scRNA-seq and Debris-seq were included in these calculations. **(D)** Frequency of each cell type across 9,929 cells. **(E)** Filtering based on DTTs detected from Clone-05 (left), Clone-25 (middle), and Clone-32 (right): Top: The number of cells detected for each DTT (y-axis) is plotted against the number of different InsertBC(1-6) combinations (x-axis). DTTs with a cell count >10 (red horizontal lines) were retained. Middle: Histograms of the numbers of post-filtering DTTs detected per cell. Cells outside the range of mean - 1.5 × SD to mean + 2 × SD were excluded (red vertical lines). Bottom: Post-filtering histograms of the total number of edited sites per cell, with the mean and standard deviation indicated. **(F)** We performed a bootstrapping analysis to evaluate the robustness of the trees shown in **Figure 2D**, composed of 2,438 tips & 2,437 clades in Clone-05, 4,418 tips & 4,417 clades in Clone-25, and 960 tips & 959 clades in Clone-32. For each of 100 bootstraps, an equal number of DTTs (each containing six edit sites) was resampled with replacement to reconstruct a new tree. Clade concordance was determined by comparing each bootstrap tree to the original and calculating transfer bootstrap expectation (TBE)[12]. Briefly, the TBE of each clade in the original tree was calculated by determining the minimal transfer distance across all clades in the bootstrap tree, followed by computing the mean values over the 100 bootstraps. Here we show a histogram of the TBEs of clades (top), and a scatter plot of TBE against the distance from the root for each clade (bottom). Dashed red vertical lines indicate a TBE cutoff of 0.7, with values greater than that corresponding to moderate to strong support[12]. **(G)** The numbers of cells in each of ten major clades (with two clades combined for Well17 and Well01, respectively) of the cell lineage tree shown in **Figure 2D** plotted against the well assignments of those same cells.

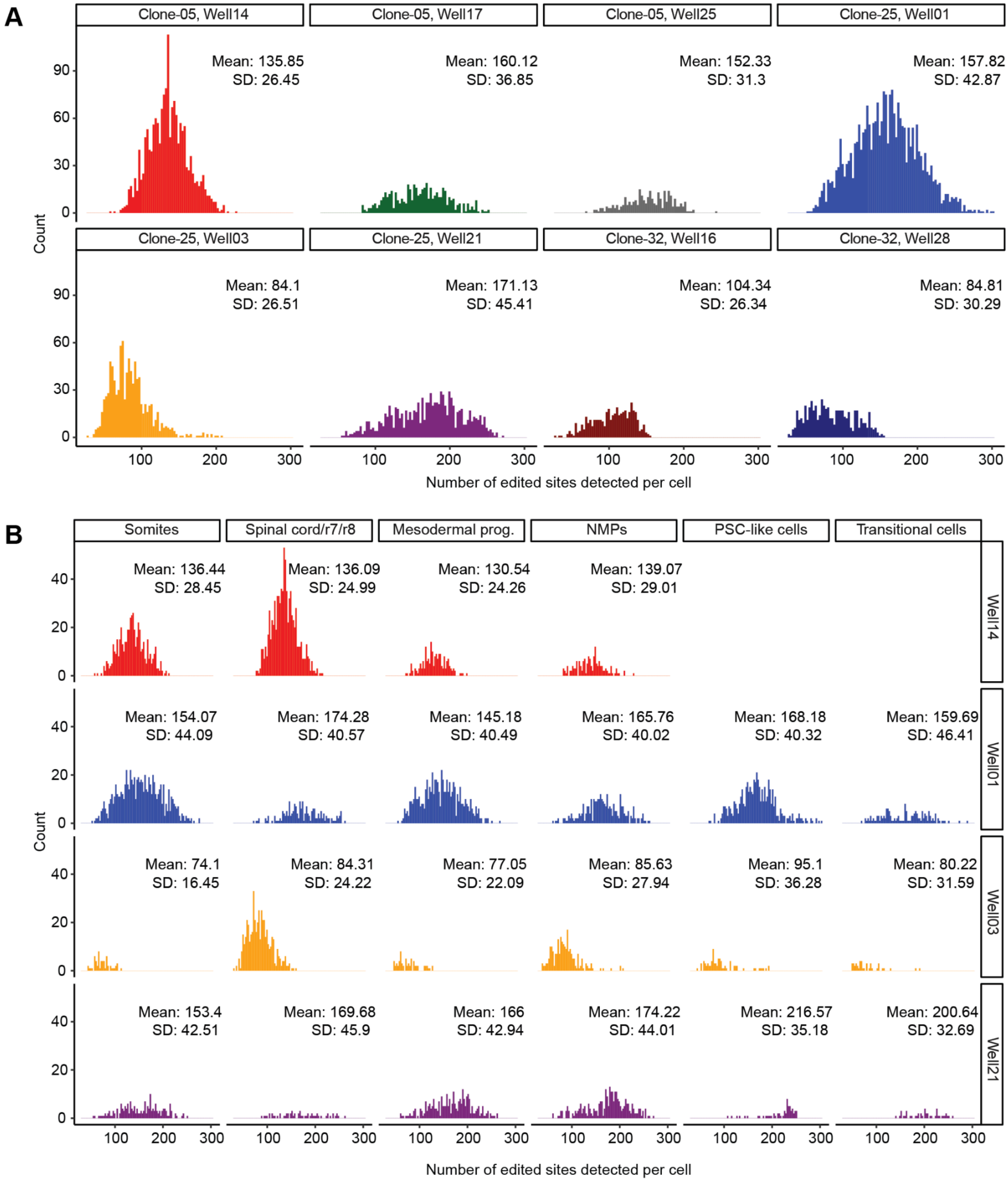

Figure S9. Numbers of edited sites in DTT detected per cell, across gastruloids and cell types (Related to Figure 2).

(A) To systematically compare the overall activity of PEmax between gastruloids and cell lines, for each DTT observed in each of the 7,816 cells used to reconstruct the lineage tree, we counted the total number of edited sites observed in each cell. Histograms of these counts are shown, together with the mean and standard deviation, for each of the eight gastruloids. **(B)** Same as **A**, but for the four gastruloids with the most sampled cells, and further splitting these out by the six most abundant cell types.

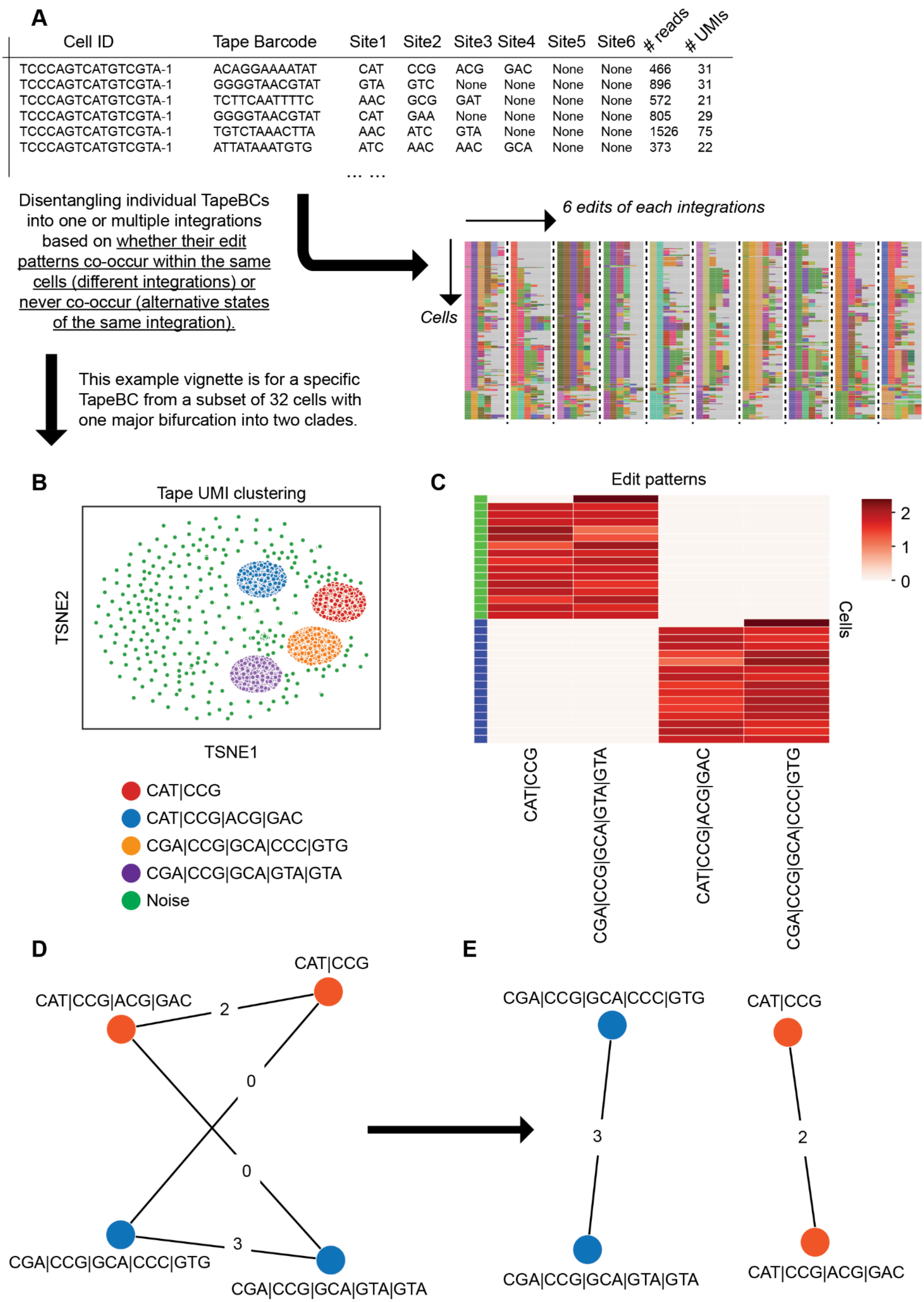
A
Cell ID
Tape Barcode
Site1
Site2
Site3
Site4
Site5
Site6
reads
UMIs
TCCCAGTCATGTCGTA-1 ACAGGAAAATAT CAT CCG ACG GAC None None 466 31
TCCCAGTCATGTCGTA-1 GGGGTAACGTAT GTA GTC None None None None 896 31
TCCCAGTCATGTCGTA-1 TCTTCAATTTTC AAC GCG GAT None None None 572 21
TCCCAGTCATGTCGTA-1 GGGGTAACGTAT CAT GAA None None None None 805 29
TCCCAGTCATGTCGTA-1 TGTCTAAACTTA AAC ATC GTA None None None 1526 75
TCCCAGTCATGTCGTA-1 ATTATAAATGTG ATC AAC AAC GCA None None 373 22
... ...
Disentangling individual TapeBCs into one or multiple integrations based on whether their edit patterns co-occur within the same cells (different integrations) or never co-occur (alternative states of the same integration).
6 edits of each integrations
Cells
This example vignette is for a specific TapeBC from a subset of 32 cells with one major bifurcation into two clades.
B
Tape UMI clustering
TSNE2
TSNE1
CAT|CCG
CAT|CCG|ACG|GAC
CGA|CCG|GCA|CCC|GTG
CGA|CCG|GCA|GTA|GTA
Noise
C
Edit patterns
2
1
0
Cells
CAT|CCG
CGA|CCG|GCA|GTA|GTA
CAT|CCG|ACG|GAC
CGA|CCG|GCA|CCC|GTG
D
CAT|CCG
CAT|CCG|ACG|GAC
2
0
0
3
CGA|CCG|GCA|CCC|GTG
CGA|CCG|GCA|GTA|GTA
E
CGA|CCG|GCA|CCC|GTG
CAT|CCG
3
2
CGA|CCG|GCA|GTA|GTA
CAT|CCG|ACG|GAC

Figure S10. Disentangling TapeBCs that are integrated at multiple genomic locations (Related to Figure 2). We relied on piggyBac transposition at a high MOI in order to obtain a large number of DTTs in each monoclonal cell line. Each DTT is associated with a degenerate TapeBC that facilitates the assignment of DTT-derived sequencing reads to specific integrations (**Figure S1F**). However, a major challenge is that due to piggyBac excision and re-integration events[2,11] during the establishment of the monoclonal cell line, a given TapeBC might be associated with two or more independent integrations, confounding lineage reconstruction. Here we present an example vignette of our strategy for disentangling such duplicated TapeBCs by relying on the patterns in which their associated edits either do (consistent with deriving from separate integrations) or don't (consistent with deriving from the same integration) appear in the same cells. Further details on our algorithmic strategy are presented in the **STAR Methods**. This example vignette is for a specific TapeBC from a subset of 32 cells with one major bifurcation into two clades. **A)** Schematic of the raw DTT data table from scRNA-seq (top left), which is processed by splitting each TapeBC into one or more integrations based on edit-pattern co-occurrence across cells, yielding the cell-by-integration matrix used for tree construction (bottom right). **(B)** t-SNE depicting clusters of DTT UMIs across all 32 cells. The labels for each cluster represent a unique pattern of edits. Each 3N edit is separated by a "|". UMIs not assigned to any cluster were discarded as likely noise from PCR or sequencing errors. **(C)** After identifying consensus edit patterns, we generated a cell-by-pattern matrix with UMI counts, row-normalized and log-transformed to highlight differences between cells. This matrix was then binarized (not shown) and the rows were clustered into two groups of cells. **(D)** The representative mutual exclusivity graph for this dataset of patterns is shown. An edge is drawn between two patterns of edits if and only if those two patterns of edits are never co-expressed in any cluster of cells in the previous matrix. Edge weights indicate the number of shared sequential edits between mutually exclusive patterns. **(E)** Graph coloring on the complement of the mutual exclusivity graph reveals the two most likely groups of mutually exclusive patterns. These groupings correspond to unique DTT integration loci of the same TapeBC.

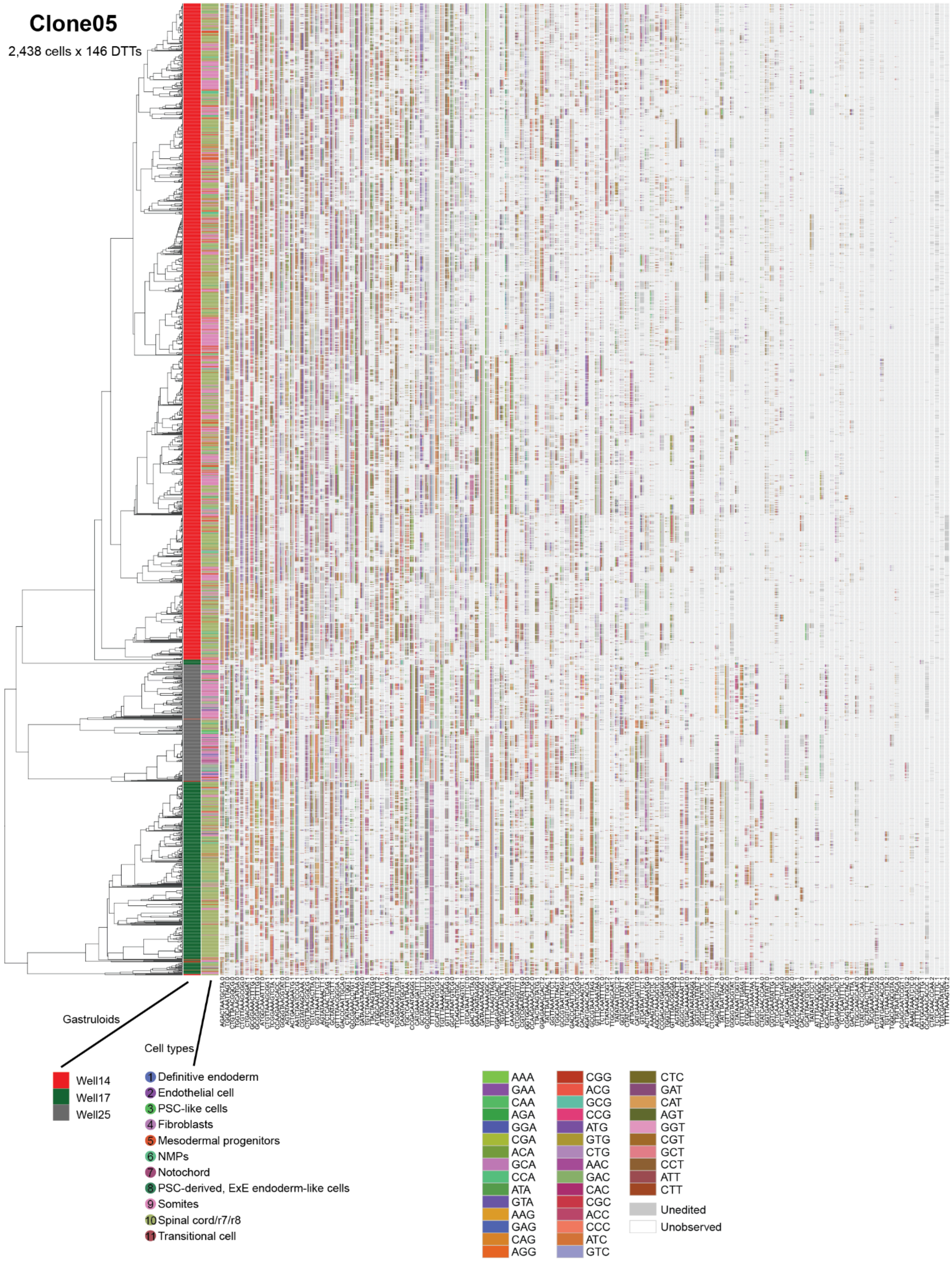

Figure S11. A cell-by-DTT matrix of Clone-05 (Related to Figure 2).

Left: The left dendrogram shows the phylogenetic tree of 2,438 cells from Clone-05 based on cell-cell distances, the colors of the middle bar correspond to 3 gastruloids to which each cell was assigned, and the colors of the right bar correspond to the 11 annotated cell types. **Right:** Edits at each site across 146 DTTs, ordered by total number of edited sites from high (left) to low (right). Observed, unedited sites are colored grey, while unobserved DTTs are colored white.

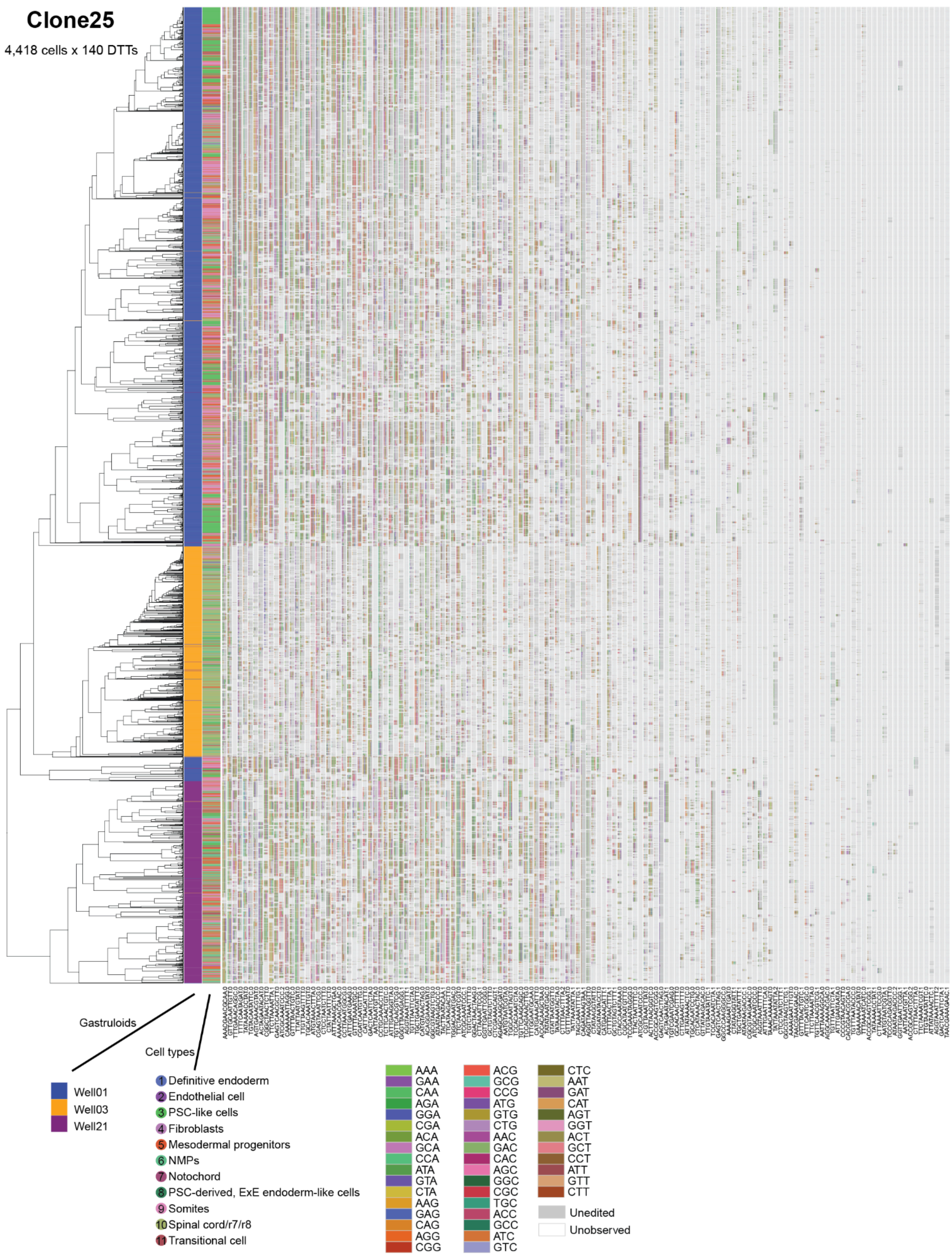

Figure S12. A cell-by-DTT matrix of Clone-25 (Related to Figure 2).
Left: The left dendrogram shows the phylogenetic tree of 4,418 cells from Clone-25 based on cell-cell distances, the colors of the middle bar correspond to 3 gastruloids to which each cell was assigned, and the colors of the right bar correspond to the 11 annotated cell types. **Right:** Edits at each site across 140 DTTs, ordered by total number of edited sites from high (left) to low (right). Observed, unedited sites are colored grey, while unobserved DTTs are colored white.

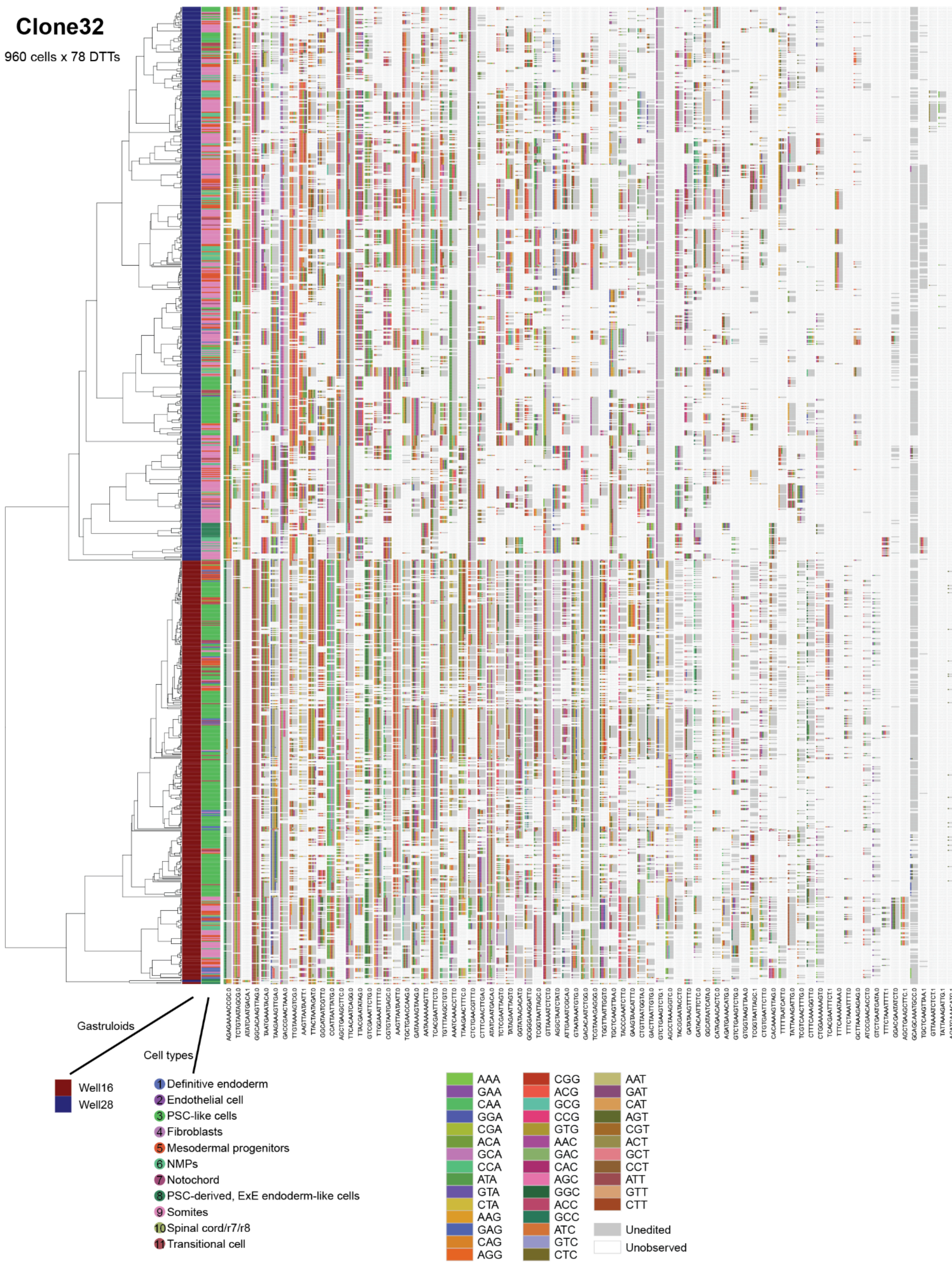

Figure S13. A cell-by-DTT matrix of Clone-32 (Related to Figure 2).
Left: The left dendrogram shows the phylogenetic tree of 960 cells from Clone-32 based on cell-cell distances, the colors of the middle bar correspond to two gastruloids to which each cell was assigned, and the colors of the right bar correspond to the 11 annotated cell types. **Right:** Edits at each site across 78 DTTs, ordered by total number of edited sites from high (left) to low (right). Observed, unedited sites are colored grey, while unobserved DTTs are colored white.

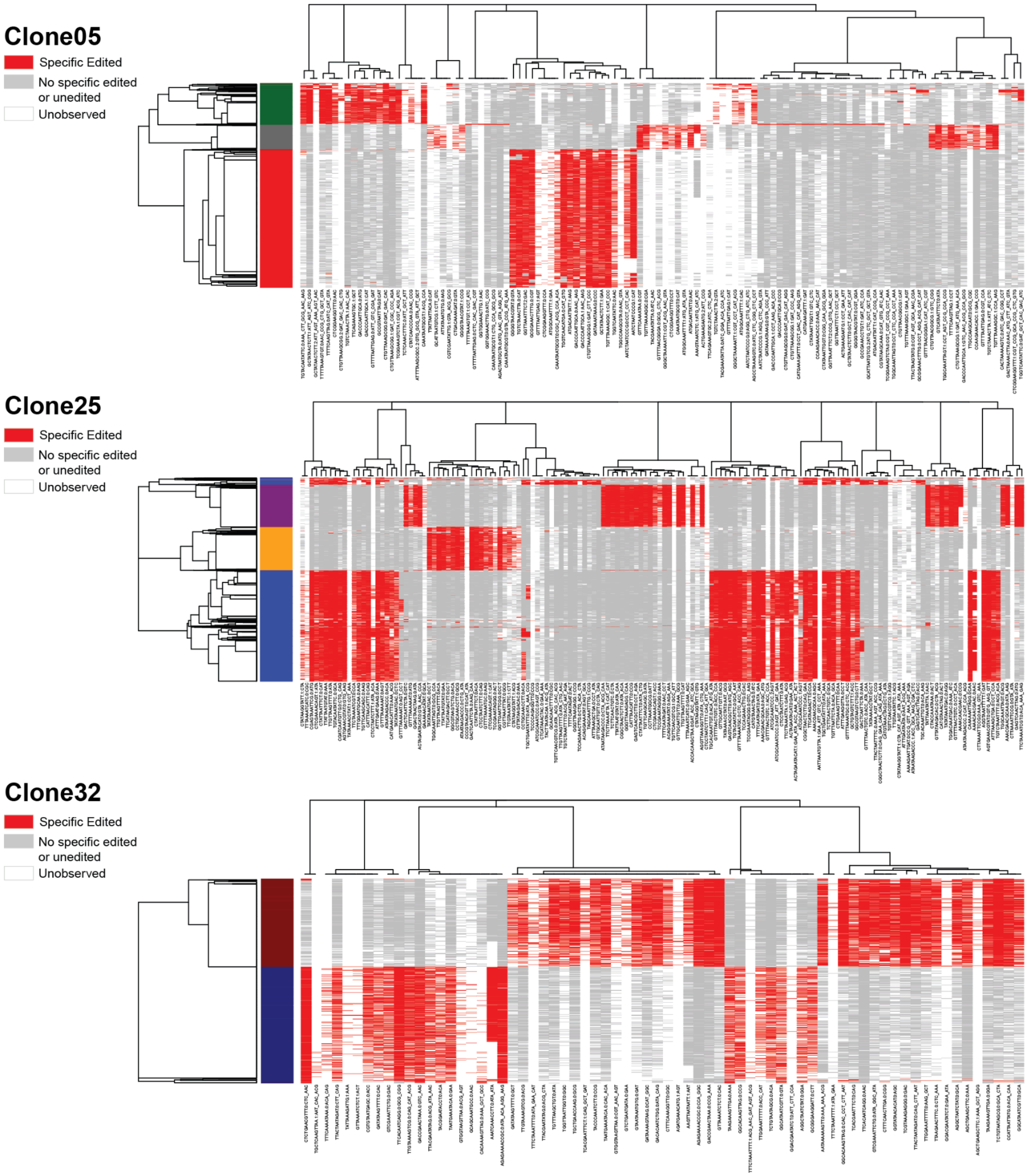

Figure S14. Each monoclonal gastruloid is defined by edits that likely occurred prior to the seeding of its founding mESC (Related to Figure 2).

As shown in **Figure 2A**, we induced PEmax by applying 100 ng/mL doxycycline beginning 24 hrs prior to sorting and seeding of single cells to individual wells. Here we show a biclustered heatmap of 114 (Clone-05), 154 (Clone-25), and 70 (Clone-32) TapeBC-Site-SpecificEdit combinations (columns), after excluding unobserved DTTs, that were detected in >95% of cells assigned to one of the ten major clades shown in **Figure 2D**, and <5% of cells of clades dominated by a different gastruloid from the same clone (rows). We detect 20 to 57 such TapeBC-Site-SpecificEdit combinations per major clade, which we infer to be editing events that occurred during the 24 hr window during which PEmax was induced but prior to seeding of single cells to individual wells.

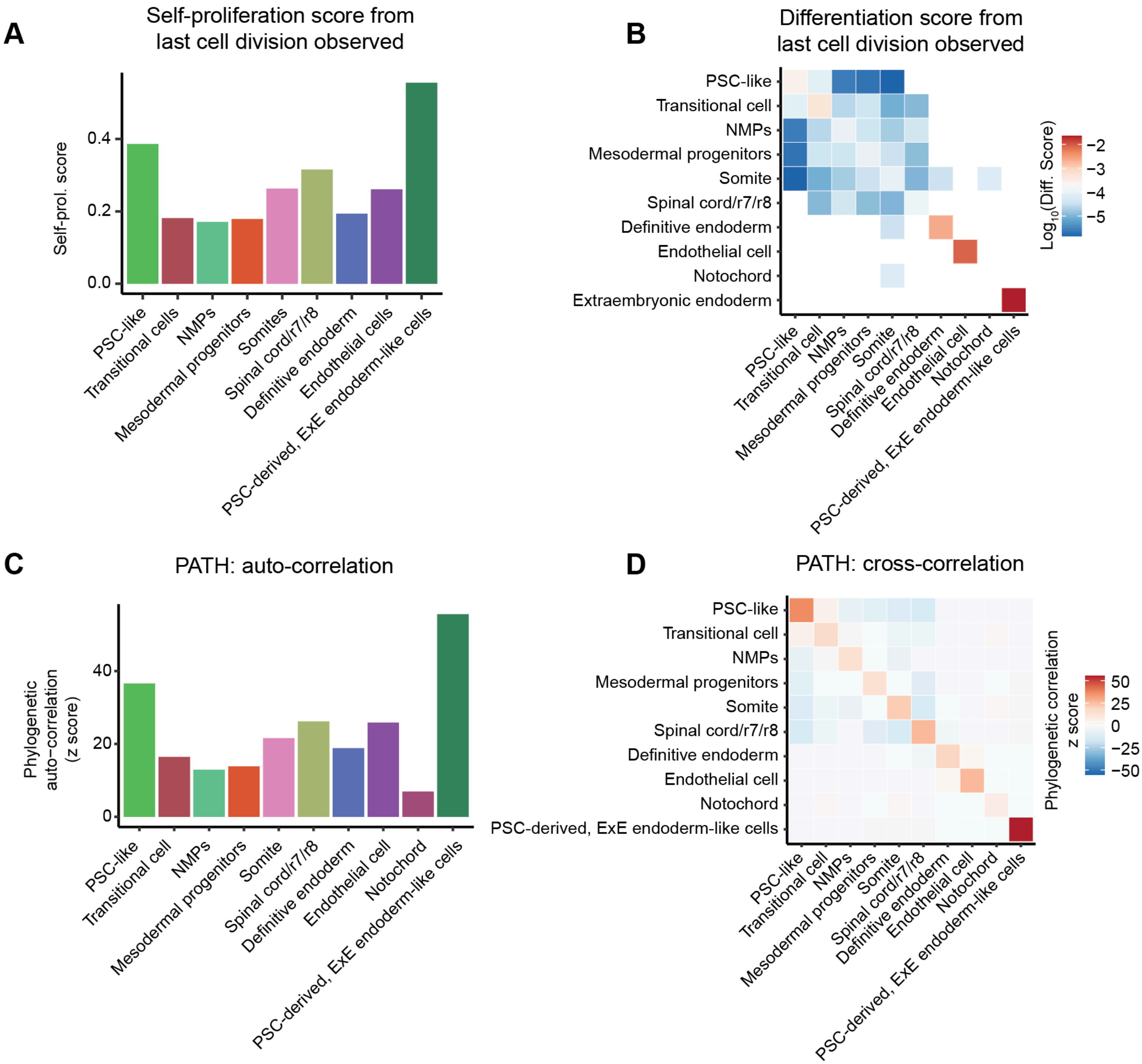

Figure S15. Quantitative assessment of cell-type self-proliferation, differentiation, and correlation in developmental lineages (Related to Figure 2).

The cell-cell distance matrices for the three clones were first combined by manually assigning the maximum distance between cells from different clones. **(A)** The self-proliferation score for each cell type was calculated as the ratio of the frequency of both cells from the last observed cell division event belonging to that cell type to the total number of cells of that type. **(B)** The differentiation score for each possible cell-type-pair was calculated as the ratio of the frequency of the pair detected from the last observed cell division event to the total frequency of all possible cell-cell combinations in the phylogenetic tree. **(C)** The auto-correlation z-score of each cell-type was calculated using PATH[13]. **(D)** The cross-correlation z-score of each possible cell-type-pair was calculated using PATH[13].

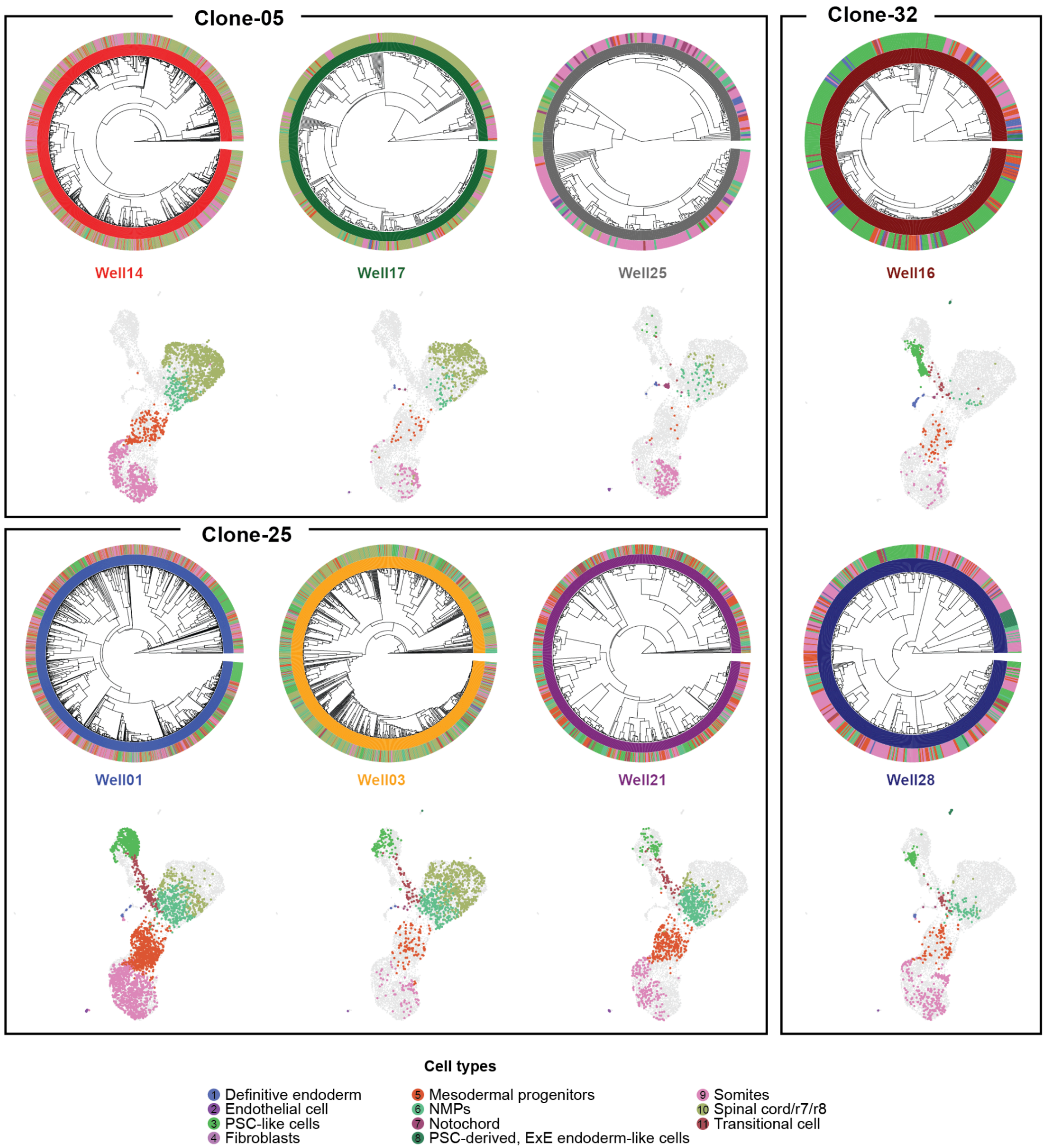

Figure S16. Cell lineage reconstructions of individual monoclonal gastruloids (Related to Figure 3).
For each monoclonal gastruloid: Top: Phylogenetic reconstruction of cell lineage relationships among higher-quality, gastruloid-assigned cells for each gastruloid. The inner circle shows the phylogenetic tree based on cell-cell edit distances, the colors of the middle circle correspond to 8 gastruloids to which each cell was assigned, and the colors of the outer circle correspond to the 11 annotated cell types. Bottom: 2D UMAP, reproduced from **Figure 2B**, showing cells assigned to individual gastruloids, colored by cell types. Gastruloids are grouped by their originating clonal cell line (*i.e.* Clone-05, Clone-25, Clone-32).

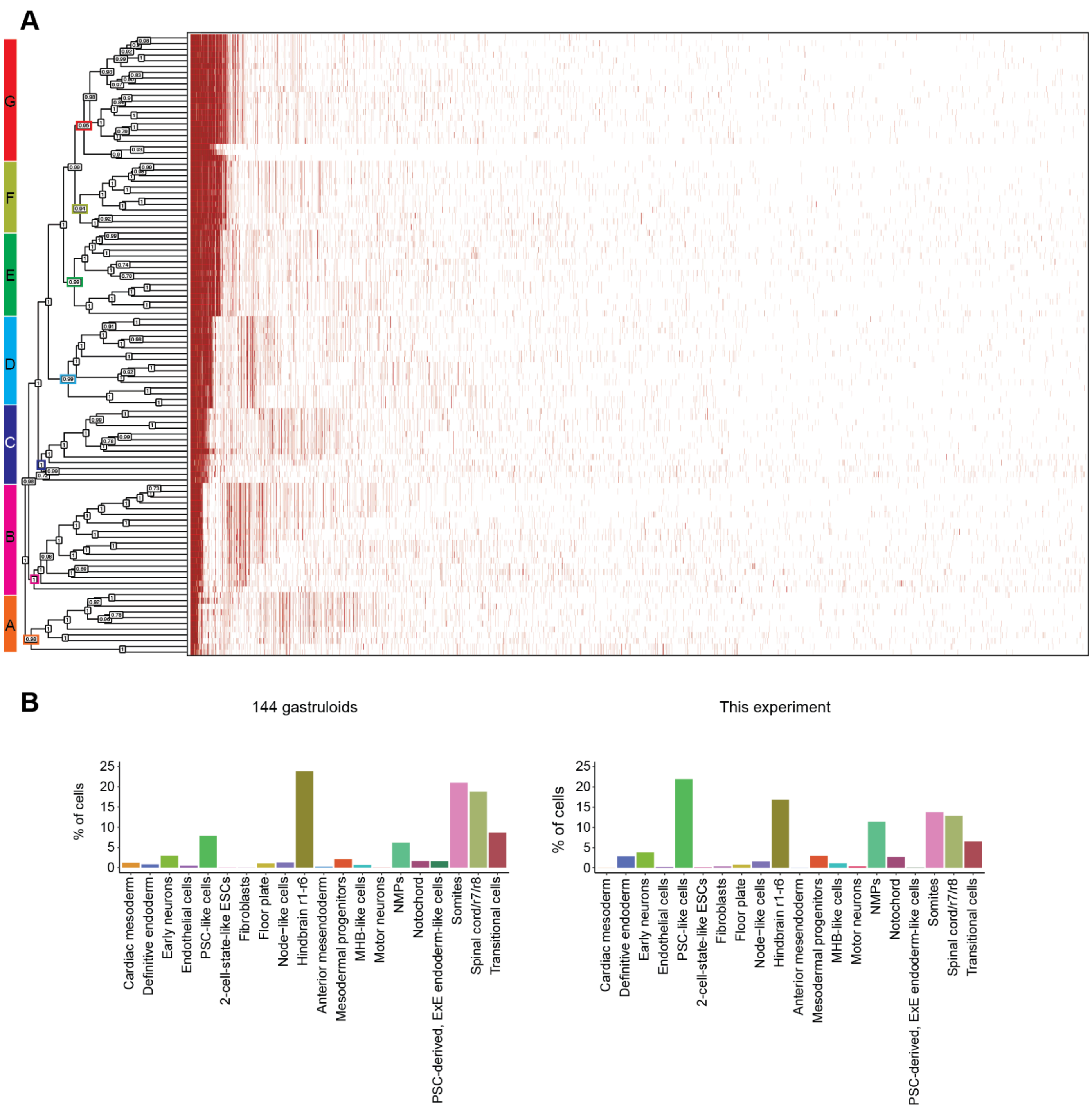

Figure S17. Phylogenetic tree of 108 gastruloids constructed using Debris-seq data (Related to Figure 4).
(A) For each gastruloid, the percentage of reads corresponding to specific edits at six sites across 76 DTTs was determined. Combinations with at least 20% of reads were identified as dominant "TapeBC-Site-Edit" combinations. The Jaccard similarity between the sets of dominant combinations was calculated, and (1 - similarity) was used as the distance metric across gastruloids. The left phylogenetic tree displays the relationships among the 108 gastruloids, based on 7,146 "TapeBC-Site-Edit" combinations shown on the right (red indicates detection in the corresponding gastruloid). The tree also includes the TBE for each ancestral node, calculated from 100 bootstraps. 106 of the 107 ancestor nodes (99%) showed moderate to strong support, with a TBE greater than 70%. The seven major clades (A-G) are highlighted on the left. **(B)** Comparison of the cell type compositions from our earlier experiment involving 144 gastruloids (247,064 cells; **Figure 1B**) and this experiment with 108 gastruloids (66,978 cells; **Figure 4C**).

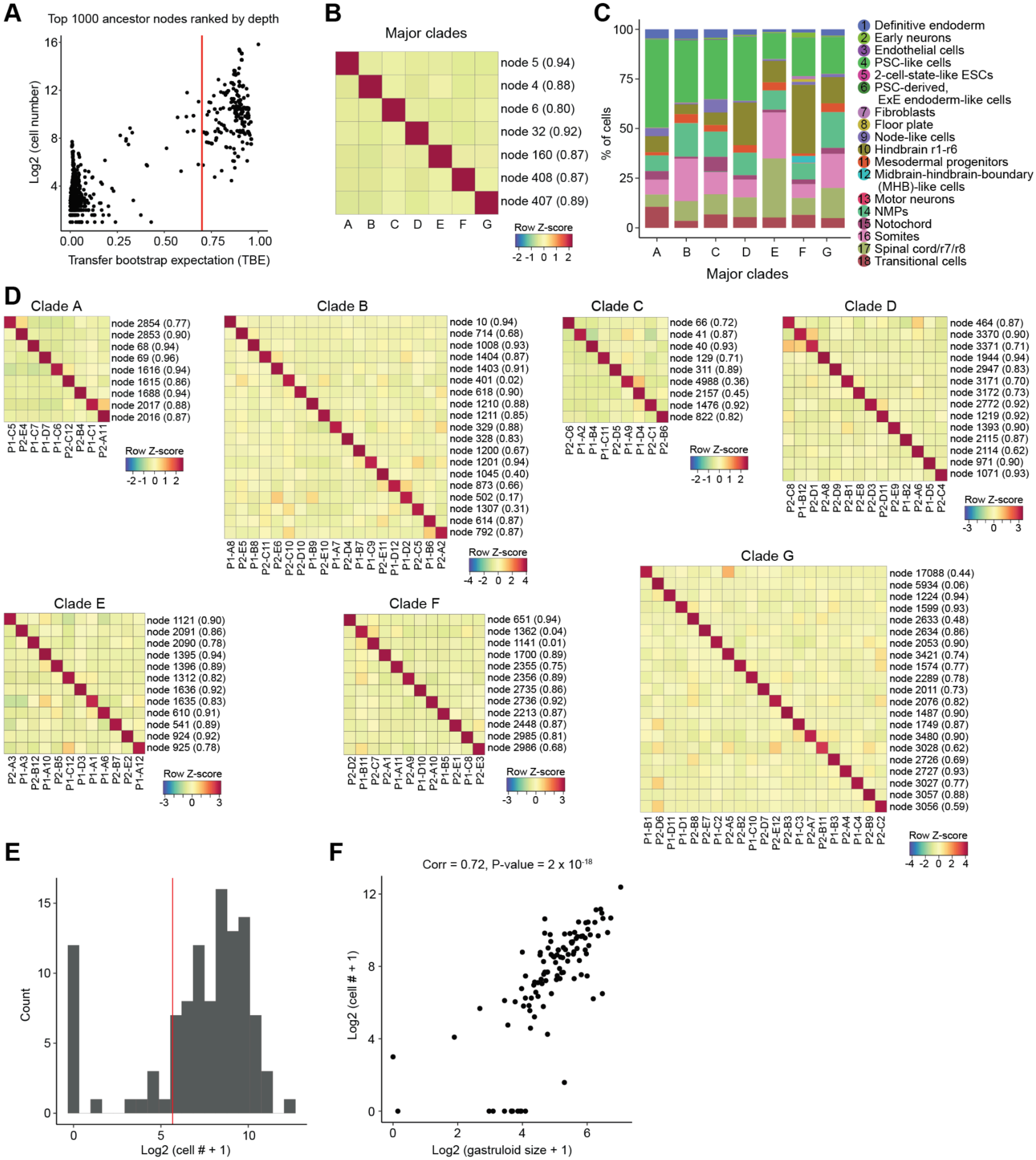

Figure S18. Identifying gastruloid founders among pseudo-ancestors (Related to Figure 4).

(A) We performed a bootstrapping analysis to evaluate the robustness of the top 1,000 clades (ranked by their distance to the root) in the tree shown in **Figure 4D**. For each of 100 bootstraps, an equal number of DTTs (each containing six edit sites) was resampled with replacement to reconstruct a new tree. Clade concordance was determined by comparing each bootstrap tree to the original and calculating transfer bootstrap expectation (TBE)[12]. Here we show a scatter plot of TBE against the number of cells for each clade. Dashed red vertical line indicates a TBE cutoff of 0.7, with 45% of clades having a TBE >0.7. **(B)** To assign each of the seven major clades from the “tree of trees” (**Figure 4B**) to a

pseudo-ancestor in the single-cell phylogenetic tree (**Figure 4D**), we constructed a cell × “TapeBC-Site-Edit” combination matrix (where 1 indicates the combination was detected in the cell and 0 was not) and normalized it by the sum of each column. For each major clade in the “tree of trees”, we identified its exclusively dominant “TapeBC-Site-Edit” combinations. For each pseudo-ancestor in the single-cell phylogenetic tree, we selected the corresponding subset of cells and calculated the fold-change between the mean value of dominant combinations for a given major clade and the mean value of dominant combinations across the other six major clades. For each major clade, we selected all pseudo-ancestors with a ratio of log2(fold-change + 1) between the given clade and the top one among the left six clades >2, and the one with the highest number of cells was designated as the representative of the major clade. The heatmap represents the log2(fold-change + 1) for each of the seven major clades and their assigned pseudo-ancestors, with the TBE for each node based on 100 bootstraps indicated in the brackets. **(C)** After assignment, cell type composition for each major clade. **(D)** For each of the seven major clades, we subset the cells from its assigned pseudo-ancestor and the gastruloids within that clade, then repeated the assignment. For each gastruloid, we identified its exclusively dominant “TapeBC-Site-Edit” combinations. Then, for each pseudo-ancestor, we calculated the fold-change between the mean value of dominant combinations for a given gastruloid and the mean across other gastruloids. For each gastruloid, we selected all pseudo-ancestors with a ratio of log2(fold-change + 1) between the given gastruloid and the top one among the left gastruloids >2, and the one with the highest number of cells was designated as the representative of the gastruloids. During this process, a total of 96 gastruloids were assigned distinct pseudo-ancestors, comprising 51,963 cells. One gastruloid was excluded due to the absence of exclusive dominant combinations, and 11 were excluded because they could not be assigned to a distinct pseudo-ancestor. The heatmap represents the log2 (fold-change + 1) for each gastruloid and their assigned pseudo-ancestors, with the TBE for each node based on 100 bootstraps indicated in the brackets. **(E)** A total of 51,963 cells were assigned to 108 wells/gastruloids, with 88 of the 108 wells/gastruloids having at least 50 assigned cells (indicated by the red vertical line). **(F)** The correlation between the number of cells assigned to individual wells/gastruloids (y-axis) and gastruloid size (x-axis) across all 108 wells/gastruloids. Pearson correlation coefficients and p-values are indicated above the plot. Sizes of individual gastruloids were measured in units of pixels^2 from their brightfield images. A threshold was applied to each image to distinguish the gastruloid from the background.

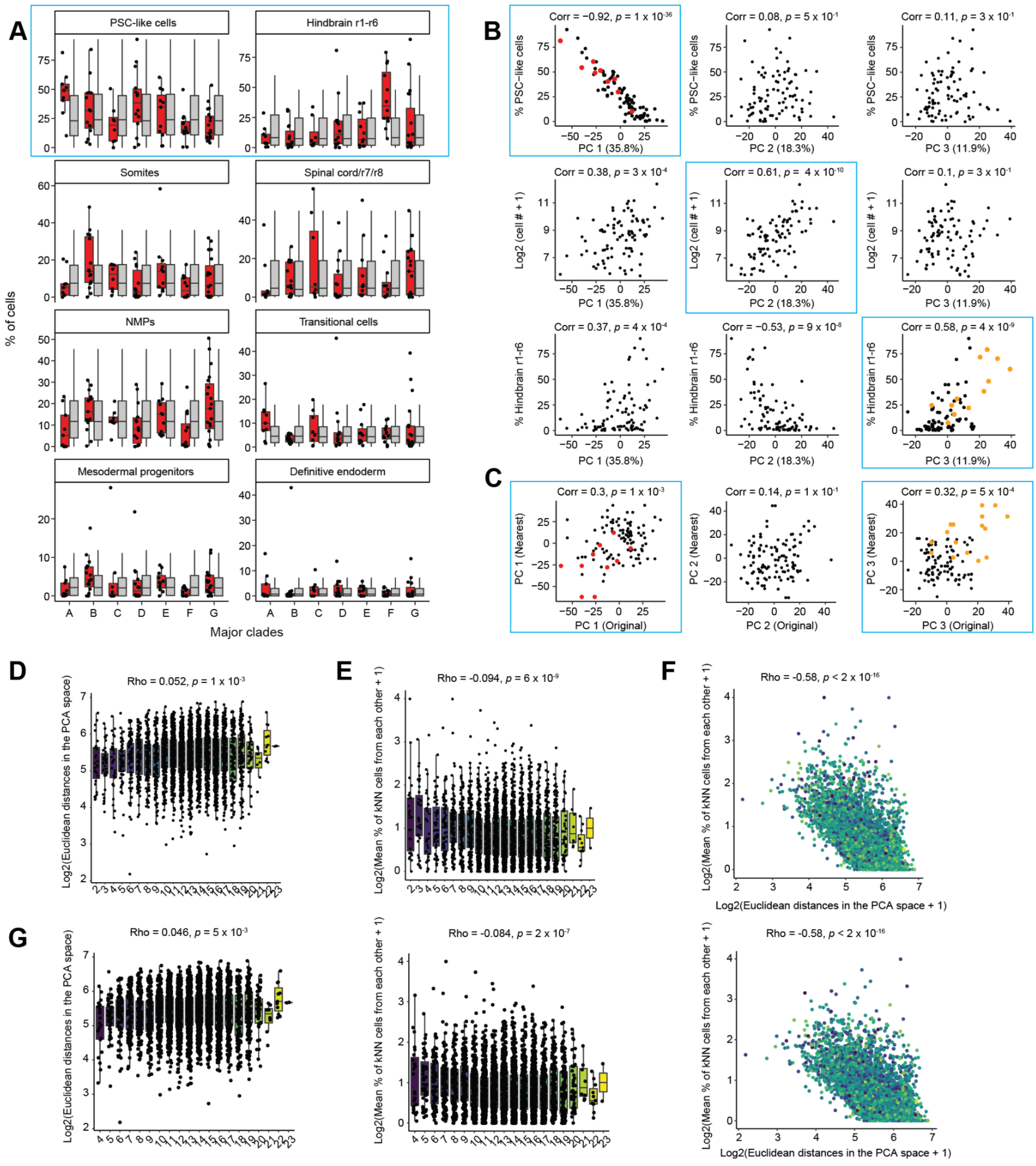

Figure S19. Cell type proportions, PCA analysis, pairwise distances, and neighboring cell relationships of monoclonal gastruloids (Related to Figure 4).

(A) The proportions of the eight most frequent cell types for 88 individual gastruloids (≥50 cells assigned) were calculated, categorized into seven groups corresponding to the major clades, as shown in **Figure 4B**. The group labels for each gastruloid were permuted 5,000 times, and cell-type proportions were recalculated each time. Boxplots, showing 88 replicates from real observations (red box) and 5,000 × 88 replicates from permutation (grey box) in each subpanel,

represent the interquartile range (25th, 50th, 75th percentiles), with whiskers extending to 1.5× the IQR. Black dots represent replicates from real observations. **(B)** Embeddings of pseudo-bulk RNA-seq profiles of 88 monoclonal gastruloids (≥50 cells assigned) were generated by aggregating single-nucleus data and performing PCA for dimensionality reduction. The percentage of cells corresponding to selected cell types (1st and 3rd rows), or the cell number (2nd row) is plotted along PC1 (left column), PC2 (middle column), and PC3 (right column). In the top left and bottom right subpanels, gastruloids from major clade A (red) and clade F (orange) were highlighted. **(C)** For each of the 88 gastruloids, their nearest neighbors were identified from the phylogenetic lineage tree, resulting in 112 pairs. Of note, some gastruloids had more than one nearest neighbor, and some pairs were redundant (both A vs. B and B vs. A were retained). For each of the top three PCs, the values for each gastruloid (x-axis) are plotted against those of their nearest neighbors (y-axis). Pearson correlation coefficients and p-values are indicated above each plot. Gastruloids from major clade A (red) and clade F (orange) are colored. Of note, subpanels highlighted with a light blue rectangle are shown in **Figure 4E-G** as well. **(D)** Pairwise Euclidean distances among 88 monoclonal gastruloids (≥50 cells assigned), calculated in 10-dimensional PCA space, are plotted against the “first epoch” lineage distances of their founder mESCs. Boxplots, encompassing a total of 3,828 pairs, represent IQR (25th, 50th, 75th percentile) with whiskers representing 1.5× IQR. Spearman correlation coefficient and p-value are indicated above. **(E)** For each gastruloid, the top 15 nearest neighboring cells for its cells were identified based on the 30-dimensional PCA space of the single-cell transcriptome. These neighboring cells were categorized by their gastruloid of origin, normalized by the total number of neighboring cells from that gastruloid, across all gastruloids. For each pair of gastruloids (e.g., A and B), the percentages of neighboring cells of A originating from B and those of B originating from A were averaged. Pairwise percentages of neighboring cells from each other among 88 gastruloids, are plotted against their lineage distances. Boxplots, encompassing a total of 3,828 pairs, represent IQR (25th, 50th, 75th percentile) with whiskers representing 1.5× IQR. Spearman correlation coefficient and p-value are indicated above. **(F)** The log2 pairwise Euclidean distances are plotted against the log2 pairwise percentages of neighboring cells for all 3,828 pairs. Spearman correlation coefficient and p-value are indicated above. **(G)** For each of the 88 gastruloids, the nearest neighbors were identified from the phylogenetic lineage tree, yielding 112 pairs. After removing redundancies (e.g., retaining only one of A vs. B and B vs. A), 76 pairs remained. These 76 pairs were excluded, and the analyses shown and described in **D-F** were repeated with the remaining 3,752 pairs.

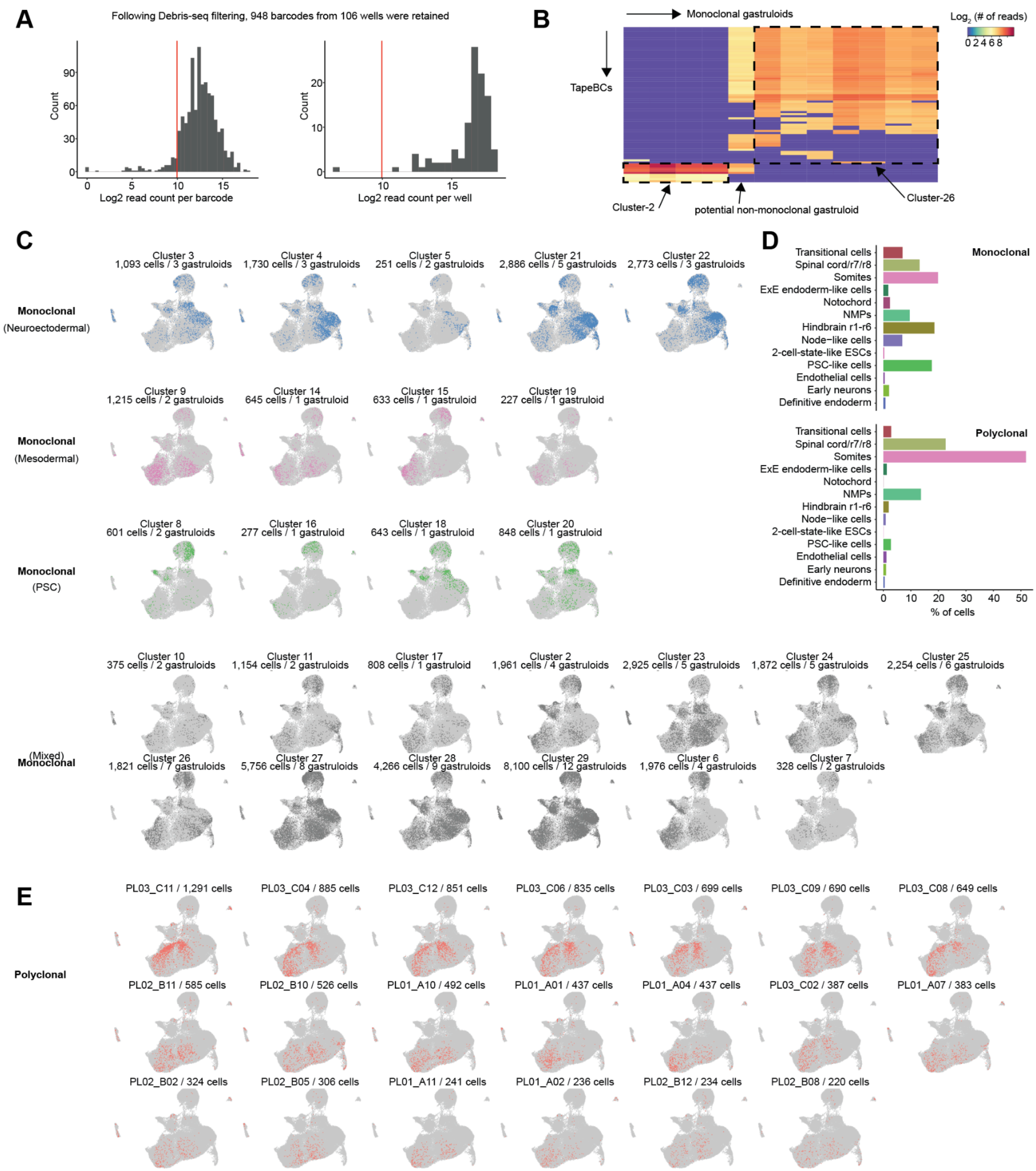

Figure S20. Assignment of cells from monoclonal and polyclonal gastruloids to clonotypes and fate-bias categories (Related to Figure 5).

(A) Histograms of log2 read counts per barcode (left) or per well (right) from Debris-seq data, comprising 1,014 barcodes across 107 wells. Barcodes and wells with fewer than 1,000 total reads were excluded (red vertical line in each plot). **(B)** A representative example of a well from Debris-seq containing barcode sets mapping to two clonal clusters, flagged as a

potential non-monoclonal gastruloid. Heatmap shows log2 read count per DTT barcode (rows) per well (columns). **(C)** The same UMAP as in **Figure 5C** is shown multiple times, with cells from a different clonal cluster (columns) of each fate-bias category (rows) highlighted. Numbers of cells and number of monoclonal gastruloids (*i.e.* wells) per clonotype are listed. **(D)** Histogram of aggregate cell type compositions of monoclonal (107 wells, 56,796 cells) and polyclonal (32 wells, 26,929 cells) gastruloids. **(E)** Same as **C**, but highlighting cells from each of 20 wells bearing polyclonal gastruloids with >200 profiled cells. Numbers of cells per polyclonal gastruloid are listed.

Supplemental references

1. van den Brink, S.C., Baillie-Johnson, P., Balayo, T., Hadjantonakis, A.-K., Nowotschin, S., Turner, D.A., and Martinez Arias, A. (2014). Symmetry breaking, germ layer specification and axial organisation in aggregates of mouse embryonic stem cells. Development *141*, 4231–4242.

2. Li, X., Chen, W., Martin, B.K., Calderon, D., Lee, C., Choi, J., Chardon, F.M., McDiarmid, T.A., Daza, R.M., Kim, H., et al. (2024). Chromatin context-dependent regulation and epigenetic manipulation of prime editing. Cell *187*, 2411–2427.e25.

3. Martin, B.K., Qiu, C., Nichols, E., Phung, M., Green-Gladden, R., Srivatsan, S., Blecher-Gonen, R., Beliveau, B.J., Trapnell, C., Cao, J., et al. (2022). Optimized single-nucleus transcriptional profiling by combinatorial indexing. Nat. Protoc. https://doi.org/10.1038/s41596-022-00752-0.

4. Pijuan-Sala, B., Griffiths, J.A., Guibentif, C., Hiscock, T.W., Jawaid, W., Calero-Nieto, F.J., Mulas, C., Ibarra-Soria, X., Tyser, R.C.V., Ho, D.L.L., et al. (2019). A single-cell molecular map of mouse gastrulation and early organogenesis. Nature *566*, 490–495.

5. Stuart, T., Butler, A., Hoffman, P., Hafemeister, C., Papalexi, E., Mauck, W.M., 3rd, Hao, Y., Stoeckius, M., Smibert, P., and Satija, R. (2019). Comprehensive Integration of Single-Cell Data. Cell *177*, 1888–1902.e21.

6. Rosen, L.U., Carine Stapel, L., Argelaguet, R., Barker, C.G., Yang, A., Reik, W., and Marioni, J.C. (2022). Inter-gastruloid heterogeneity revealed by single cell transcriptomics time course: implications for organoid based perturbation studies. bioRxiv, 2022.09.27.509783. https://doi.org/10.1101/2022.09.27.509783.

7. van den Brink, S.C., Alemany, A., van Batenburg, V., Moris, N., Blotenburg, M., Vivié, J., Baillie-Johnson, P., Nichols, J., Sonnen, K.F., Martinez Arias, A., et al. (2020). Single-cell and spatial transcriptomics reveal somitogenesis in gastruloids. Nature *582*, 405–409.

8. Multimodal characterization of murine gastruloid development (2023). Cell Stem Cell *30*, 867–884.e11.

9. Veenvliet, J.V., Bolondi, A., Kretzmer, H., Haut, L., Scholze-Wittler, M., Schifferl, D., Koch, F., Guignard, L., Kumar, A.S., Pustet, M., et al. (2020). Mouse embryonic stem cells self-organize into trunk-like structures with neural tube and somites. Science. https://doi.org/10.1126/science.aba4937.

10. Qiu, C., Martin, B.K., Welsh, I.C., Daza, R.M., Le, T.-M., Huang, X., Nichols, E.K., Taylor, M.L., Fulton, O., O'Day, D.R., et al. (2024). A single-cell time-lapse of mouse prenatal development from gastrula to birth. Nature *626*, 1084–1093.

11. Wilson, M.H., Coates, C.J., and George, A.L., Jr (2007). PiggyBac transposon-mediated gene transfer in human cells. Mol. Ther. *15*, 139–145.

12. Lemoine, F., Domelevo Entfellner, J.-B., Wilkinson, E., Correia, D., Dávila Felipe, M., De Oliveira, T., and Gascuel, O. (2018). Renewing Felsenstein's phylogenetic bootstrap in the era of big data. Nature *556*, 452–456.

13. Schiffman, J.S., D'Avino, A.R., Prieto, T., Pang, Y., Fan, Y., Rajagopalan, S., Potenski, C., Hara, T., Suvà, M.L., Gawad, C., et al. (2024). Defining heritability, plasticity, and transition dynamics of cellular phenotypes in somatic evolution. Nat. Genet. https://doi.org/10.1038/s41588-024-01920-6.
